# Robust HLA-B-restricted CD8+ T-cell responses in chronic HBV infection^☆^

**DOI:** 10.1016/j.jhepr.2026.101868

**Published:** 2026-04-24

**Authors:** Julia Lang-Meli, Anna-Lena Denecke, Johannes Ptok, Philipp Ehrenmann, Elahe Salimi Alizei, Hendrik Luxenburger, Michelle Maas, Muthamia Kiraithe, Felix Jacobi, Giuseppe Rusignuolo, Isabel Schulien, Emma Gostick, Sian Llewellyn-Lacey, Florian Emmerich, Bertram Bengsch, Tobias Boettler, David A. Price, Andreas Walker, Jörg Timm, Robert Thimme, Maike Hofmann, Christoph Neumann-Haefelin

**Affiliations:** 1 Department of Medicine II (Gastroenterology, Hepatology, Endocrinology and Infectious Diseases), Freiburg University Medical Center, Faculty of Medicine, University of Freiburg, Freiburg, Germany; 2 Department of Gastroenterology and Hepatology, University Hospital Cologne, Faculty of Medicine, University of Cologne, Cologne, Germany; 3 Center for Molecular Medicine Cologne (CMMC), University of Cologne, Cologne, Germany; 4 Faculty of Biology, University of Freiburg, Freiburg, Germany; 5 Institute of Virology, Heinrich-Heine-University, University Hospital, Duesseldorf, Germany; 6 Department of Dermatology and Venereology, Freiburg University Medical Center, Faculty of Medicine, University of Freiburg, Freiburg, Germany; 7 Division of Infection and Immunity, Cardiff University School of Medicine, University Hospital of Wales, Cardiff, UK; 8 Institute for Transfusion Medicine and Gene Therapy, Freiburg University Medical Center, Faculty of Medicine, University of Freiburg, Freiburg, Germany; 9 Systems Immunity Research Institute, Cardiff University School of Medicine, University Hospital of Wales, Cardiff, UK

**Keywords:** Hepatitis B virus (HBV), T cell, Human leukocyte antigen (HLA), Major histocompatibility complex (MHC), Viral escape

## Abstract

**Background & Aims:**

The function and quantity of CD8+ T-cell responses is superior in acute *vs.* chronic HBV infection. However, whether different HBV-derived epitopes are targeted in these distinct courses of HBV infection remains unclear.

**Methods:**

We screened peripheral blood mononuclear cells from 56 patients with chronic HBV infection genotype D and 32 patients with acute/resolved HBV infection to identify responses to overlapping peptides (18mers) covering the full HBV genotype D proteome. We then performed experimental fine-mapping of the minimal optimal epitopes.

**Results:**

Patients with acute HBV infection showed a broad HBV-specific CD8+ T-cell epitope repertoire. After spontaneous resolution, the strength of HBV-specific CD8 T-cell responses decreased, whereas the broad epitope landscape was preserved. In chronic HBV infection, the specificity of HBV-specific CD8+ T-cell responses to HBV antigens was shifted, with a lack of functional HBsAg-specific CD8+ T-cell responses (*p* = 0.0007), as recently described. Interestingly, patients with chronic HBV infection showed significantly more HLA-B-restricted HBV-specific CD8+ T-cell responses than HLA-A-restricted responses (33 *vs.* 19), whereas this distribution was balanced in patients with acute/resolved HBV infection (16 *vs.* 27; *p* = 0.0364). Most (79.6%) of the detected responses showed conserved autologous viral sequences. This observation was confirmed in a broader sequence dataset with no evidence for HLA-B-driven CD8+ T-cell selection pressure.

**Conclusions:**

In contrast to acute/resolved infection, conserved HLA-B-restricted epitopes are dominant in chronic HBV infection, making them interesting candidates for immunotherapeutic approaches towards functional cure of chronic HBV infection.

**Impact and implications:**

To date, there is no treatment to achieve functional cure of chronic HBV infection. Immunotherapy boosting dysfunctional HBV-specific CD8+ T cells is an interesting approach, but relies on selection of optimal target epitopes. Using an unbiased approach, we found that the HBV-specific CD8+ T-cell epitope repertoire in chronic HBV infection is not well covered by previously described epitopes. We characterized 28 novel, primarily HLA-B-restricted epitopes that are dominantly targeted in chronic HBV infection. These might be used as a “toolbox” for immunological studies and immunotherapeutic approaches.

## Highlights

• “Toolbox” of 28 novel HBV-specific CD8+ T-cell epitopes primarily targeted in chronic HBV infection.
• Increased targeting of HBV pol- and X-derived CD8+ T-cell epitopes in chronic *vs.* acute/resolved HBV infection.
• Dominance of functional HLA-B-restricted epitopes in chronic *vs.* acute/resolved HBV infection.

## Graphical abstract

**Figure:**
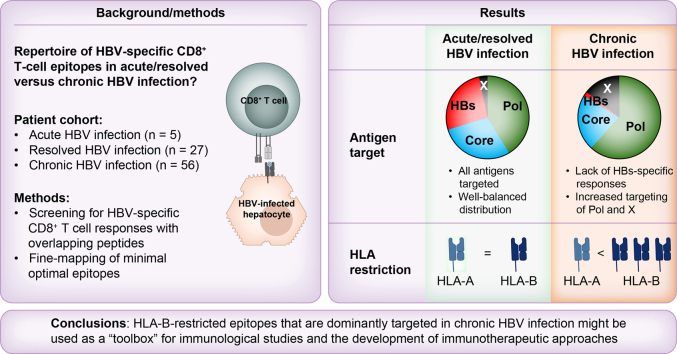

## Introduction

Chronic HBV infection is a major global health burden accounting for >750,000 deaths annually. Most of these result from HBV-associated complications such as liver cirrhosis and hepatocellular carcinoma. Despite the availability of a safe and effective vaccine, the incidence of HBV infection remains high in the African and South East Asian region.^1^^,^^2^ For patients with chronic HBV infection, treatment achieving functional cure is still lacking. One promising strategy is to enhance the HBV-specific CD8+ T-cell response by immunotherapeutic approaches like therapeutic vaccination.^3^ The development of such therapies requires a detailed understanding of the HBV-specific CD8+ T-cell response. The choice of the ideal target antigen/epitopes is crucial in this context.^4^ However, which epitopes are targeted in acute/resolved *vs.* chronic HBV infection remains incompletely understood. This open aspect in HBV immunobiology is of special importance, as most HBV-specific CD8+ T-cell epitopes have been identified in acute/resolved HBV infection, indicating a substantial bias when these epitopes are used to analyze HBV-specific CD8+ T-cell immunity in chronic HBV infection. More importantly, targeting these epitopes by immunotherapeutic approaches in chronic HBV infection may be of limited benefit. We thus addressed this knowledge gap with a detailed unbiased analysis of the HBV-specific CD8+ T-cell landscape in age- and HLA-matched patients with HBV infection of different courses.

## Materials and methods

### Study cohort

In this study, 56 patients with chronic HBV infection (all HBeAg negative and without evidence for liver cirrhosis, nucleos(t)ide analogs-treated if clinically indicated), five patients with acute HBV infection, and 27 patients with serological evidence for resolved HBV infection were included. All patients were recruited at the Freiburg University Medical Center, Freiburg, Germany. HLA typing was performed using next-generation sequencing. Donor characteristics are summarized in Table S1.

### Ethics

Written informed consent was obtained from all participants before inclusion. The study was conducted according to federal guidelines and local ethics committee regulations (Albert-Ludwigs-Universität, Freiburg, Germany; vote: 383/19, 322/20, 21-1135, and 315/20) and the Declaration of Helsinki (1975).

### Isolation of PBMCs

Peripheral blood mononuclear cells (PBMCs) were isolated, stored, and thawed as described previously.^5^

### Peptides

We synthesized 225 overlapping peptides (OLPs) spanning the whole HBV proteome genotype D (*ayw* subtype, GenBank accession number: X02496) as 18mers overlapped by 11 amino acids and containing free amine NH2 and COOH termini. These peptides were produced by Genaxxon Bioscience (Ulm, Germany) with a purity >70%.

### *In vitro* expansion and intracellular IFN-γ staining with OLPs

We conducted *in vitro* expansion of PBMCs as described previously.^6^ On Day 10, we screened for OLP-specific interferon gamma (IFN-γ) production via enzyme-linked immunospot (ELISpot) assay after 24-h stimulation with peptide pools in a 45 × 10 matrix setup. Each OLP was included in two peptide pools of the matrix (5 μM, 5 × 10^4^ cell/well) with two positive wells defining one positive individual OLP. ELISpot was performed following manufacturer’s instructions (BD Biosciences, Franklin Lakes, NJ, USA; 551951, 551873, 557630, and 551958), with the following concentrations: 0.05 μl/ml capture antibody, 0.04 μl/ml detection antibody, 0.1 μl/ml streptavidin-horseradish peroxidase, 0.02 μl/ml AEC chromogen. Positive wells in ELISpot were defined with >2× mean spot forming units of the negative controls (triplicate). The identified individual IFN-y-producing OLPs were subsequently validated on Days 12–14 by intracellular cytokine staining (ICCS) and flow cytometry. In particular, cells were restimulated with individual OLPs (5 μM), dimethyl sulfoxide as negative control or phorbol 12-myristate 13-acetate and ionomycin as positive control in the presence of brefeldin A and IL-2. After 5 h of incubation at 37 °C, cells were stained for surface markers (CD8+, CD4+; 7-amino-actinomycin D) and intracellular markers (IFN-γ). To test for specificity of the HBV OLP approach, four healthy donors were tested as described above without any detection of CD8+ T-cell responses.

### “Fine-mapping” of minimal optimal epitopes

We used the Immune Epitope Database (IEDB) to screen viral amino acid sequences of positive individual OLPs in ICCS for previously described minimal epitopes matching the patient’s HLA type. If no previously described epitope was found, we predicted candidates using two prediction algorithms ANN 4.0 and NetMHCpan EL 4.123 for 8-mer, 9-mer, and 10-mer peptides with half-maximal inhibitory concentration (IC_50_) of <500 nM. These were subsequently tested in the respective patient by ICCS.

### Experimental determination of HLA restriction

Four partially HLA-matched EBV-transformed B-lymphoblastoid cell lines (B-LCLs), sharing only one HLA class I allele with the HBV-specific effector CD8 T cell along with three irrelevant HLA types, were selected. B-LCLs were pulsed with the epitope peptide overnight, then washed six times, and used for stimulation (ICCS) of the epitope-specific T-cell line from the respective patient.

### Flow cytometry for T-cell analysis

Analyses were performed on FACSCanto II™ with FACSDiva software version 10.6.2 (BD Biosciences) or CytoFLEX (Beckman Coulter, Brea, CA, USA) with CytExpert software version 2.3.0.84 (Beckman Coulter Inc, Brea, CA, USA) after fixation of cells in 2% paraformaldehyde (Sigma). Data were then analyzed with FlowJo 10.7.1 (Tree Star Inc., Ashland, OR, USA). The gating strategy is displayed in Fig. S4.

### Amplification and sequence analysis of the HBV genome

Autologous viral sequences of patients with CD8+ T-cell responses were obtained by Sanger sequencing (Eurofins Scientific SE, Luxemburg, Luxemburg) after purification of viral DNA from patient plasma using the QIAamp DNA Blood Mini Kit (Qiagen) and amplification via nested PCR and primers (Table S4).

### Detection of HLA-associated mutations in CD8+ T-cell epitopes with HAMdetector

We analyzed a set of 239 previously published^7^ sequences of patients with known HLA alleles (all HBV genotype D; all HBeAg negative) using HAMdetector^8^ for HLA-associated viral sequence polymorphisms within the targeted HBV-specific CD8+ T-cell epitopes. The program is implemented as a Julia package for identifying HLA-associated substitutions based on aligned viral protein sequences paired to host HLA class I data. It integrates information from epitope prediction via MHCflurry 2.0 and genome phylogeny (based on RAxML-NG). The complete source code and documentation is available on GitHub online platform.^9^

### MEME

Sites in the epitopes that evolved under positive selection were detected with the maximum likelihood approach *mixed effects model of evolution* (MEME) on the Datamonkey server with default parameters.^10^^,^^11^

### SeqFeatR

In addition to HAMdetector analysis, HLA-associated viral sequence polymorphisms of all HBV proteins were also tested with the R package SeqFeatRas previously described (R Foundation for Statistical Computing, Vienna, Austria).^12^

### Statistical analysis

We performed statistical analysis using non-parametric tests with Prism 9 (GraphPad Software, San Diego, CA, USA). A *p* value <0.05 was considered as statistically significant.

## Results

### Differential T-cell response in acute/resolved *vs.* chronic infection

**Fig. 1:**
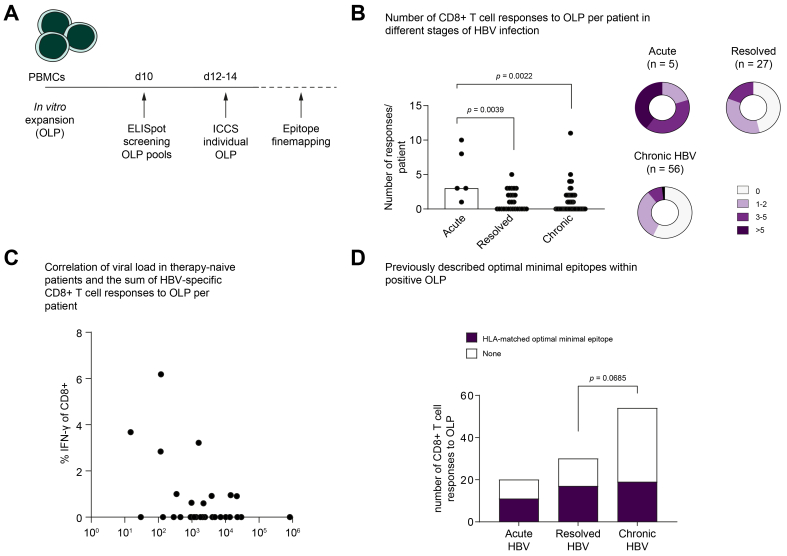
Comprehensive fine-mapping of the CD8+ T-cell epitope repertoire in chronic *vs.* acute/resolved HBV infection. (A) Experimental setup. (B, left panel) Number of HBV-specific CD8+ T-cell responses to OLPs in patients with acute, resolved, or chronic HBV infection. Median is indicated and statistical analysis was performed with Kruskal-Wallis test with false-discovery rate correction using a step-up procedure of Benjamini, Krieger, and Yekutieli. (B, right panel) Proportion of CD8+ T-cell responses to OLPs in acute, resolved, or chronic HBV infection targeting different HBV proteins. (C) Correlation between the sum of HBV-specific CD8+ T-cell responses per patient and viral load in patients with chronic HBV infection. Statistical analysis was performed with Spearman correlation. (D) Percentage of positive OLPs with previously described optimal minimal epitope matching the respective patients’ HLA type. Statistical analysis was performed with two-tailed Fisher’s exact test. HLA, human leukocyte antigen; OLPs, overlapping peptides.

To map the HBV-specific CD8+ T-cell response in detail, we tested patients with acute (n = 5), resolved (n = 27), or age- and HLA-matched chronic HBV infection (n = 56, all HBeAg negative; Table S1; Fig. S1A and B) for responses to pools of OLPs spanning the whole HBV proteome using ELISpot assay. Positive pools were evaluated for the triggering individual OLPs by flow cytometry after intracellular IFN-γ staining (ICCS) (Fig. 1A). In line with previous reports,^13^ we found fewer CD8+ T-cell responses to OLPs per patient in patients with chronic compared with acute HBV infection (Fig. 1B). Patients with resolved HBV infection also showed fewer CD8+ T-cell responses to OLPs compared with patients with acute HBV infection. Furthermore, the number of detected T-cell responses per patient was not different in resolved versus chronic HBV infection (Fig. 1B). All patients with acute HBV infection showed at least one HBV-specific CD8+ T-cell response to OLPs; however, we did not find HBV-specific CD8+ T-cell responses to any OLPs in about half of patients with resolved or chronic HBV infection (Fig. 1B). In therapy-naïve patients with chronic HBV infection, the sum of HBV-specific IFN-γ CD8+ T-cell responses per patients was not significantly correlated with viral load (Fig. 1C).

For each positive individual OLP, we searched the IEDB website (https://www.iedb.org) for previously described minimal optimal epitopes matching the patient’s HLA type (Fig. 1D). Minimal optimal epitopes were identified more frequently with respect to T-cell responses from patients with acute (55%) or resolved HBV infection (56%) compared with chronic HBV infection (32.6%). As databases such as IEDB rely on previously identified epitopes, this suggests that the HBV-specific CD8+ T-cell epitope repertoire in chronic HBV infection remains understudied. This is inadequately represented by currently described HBV-specific CD8+ T-cell epitopes, which have primarily been identified in acute/resolved HBV infection.

### Comprehensive fine-mapping of the CD8+ T-cell epitope repertoire in chronic *vs* acute/resolved HBV infection

**Fig. 2:**
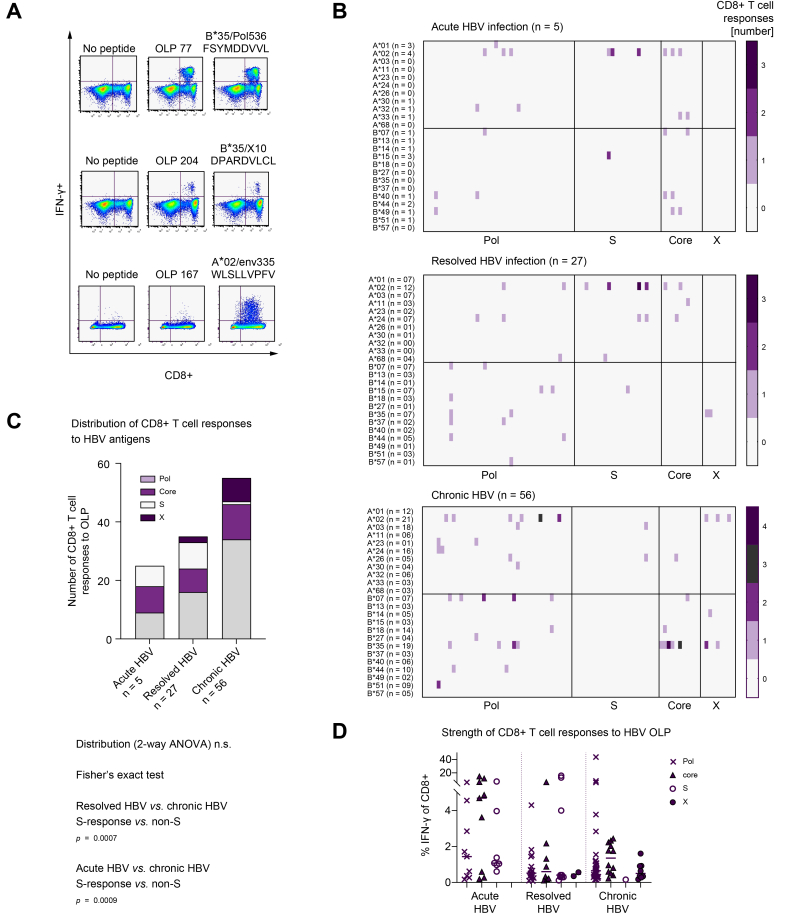
Shift of antigen target of HBV-specific CD8+ T-cell responses in chronic *vs.* acute/resolved HBV infection. (A) Representative staining of experimental fine-mapping of minimal optimal epitope within positive OLPs. (B) Number, location, and HLA restriction of HBV-specific CD8+ T-cell responses to OLPs spanning the whole HBV proteome in patients with acute, resolved, or chronic HBV infection. (C) Percentage of HBV-specific CD8+ T-cell responses targeting different HBV proteins in acute/resolved *vs.* chronic HBV infection. (D) Strength of HBV-specific CD8+ T-cell responses to HBV OLPs targeting different HBV proteins. (E) Statistical analysis was performed with two-way ANOVA with false-discovery rate correction for multiple comparisons using a step-up procedure of Benjamini, Krieger, and Yekutieli or as indicated. OLPs, overlapping peptides.

We then aimed to address the identified knowledge gap of the optimal epitope repertoire in chronic HBV infection and comprehensively characterized the HBV-specific CD8+ T-cell epitope repertoire with a focus on chronic HBV infection. To do so, we performed *in silico* fine-mapping using two prediction methods (ANN and NetMHCpan on the IEDB webpage) for positive OLPs to predict HLA class I restriction and optimal epitope sequence and length based on the HLA class I alleles expressed by the respective patient. The epitope candidates were subsequently experimentally further validated by ICCS (Fig. 2A). For a subset of responses with inconclusive results from prediction, we also used partially HLA-matched immortalized B cell lines to experimentally assess HLA restriction of the CD8+ T-cell response to the OLP or minimal epitope (Fig. S1C). In total, HLA restriction and optimal epitope sequence were previously described or experimentally determined for 83 of the 104 (79.8%) CD8+ T-cell responses. For the remaining 21 CD8+ T-cell responses, no previously described HLA-matched optimal epitope was available, and experimental fine-mapping was not possible because of limited sample availability. In these cases, the best *in silico* predicted candidate was inferred. Specificity of assessed HLA restriction was further confirmed by HLA-mismatch experiments. PBMCs of five patients with chronic HBV infection were tested after *in vitro* expansion with all epitopes targeted in our study not matching their respective HLA type (Table S5). Epitopes restricted by HLA types belonging to the same HLA supertype family^14^ as the patient’s HLA type were excluded for possible cross-recognition. With this approach, overall 176 staining were negative. We found only one weak (0.16% IFN-γ+ of CD8+) CD8+ T-cell response to epitope B∗18/Core7 (KEFGATVEL) from a patient with HLA alleles A∗01:01; A∗02:01; B∗35:03; B∗51:01. A detailed list of all positive HBV-specific CD8+ T-cell responses to OLPs with the respective fine-mapping of the minimal optimal epitope is provided in Table S2. With this approach, we were able to experimentally fine-map and characterize 28 novel HBV-specific CD8+ T-cell epitopes (Table S3).

We were further able to compare the HBV-specific CD8+ T-cell epitope repertoire in different cohorts on the population level (Fig. 2B, acute patients are presented individually in Fig. S2). Interestingly, the landscape of HBV-specific CD8+ T-cell responses showed a similar pattern in acute/resolved HBV infection. In contrast, the epitope landscape in chronic HBV infection was different compared to acute/resolved HBV infection.

### HBV-specific CD8+ T-cell responses target HBV antigens differentially in chronic *vs*. acute/resolved HBV infection

In line with previous reports,[15], [16], [17] we found a broad HBV-specific CD8+ T-cell epitope repertoire in patients with acute/resolved HBV infection targeting different HBV antigens (Fig. 2B and C). Patients with chronic HBV infection showed fewer surface-specific CD8+ T-cell responses to OLPs than patients with acute/resolved HBV infection, as recently described.^18^ We found a trend towards more polymerase- and HBx-specific CD8+ T-cell responses in patients with chronic HBV infection, while the HBV core antigen was targeted by a similar percentage of CD8+ T-cell responses from patients with acute, resolved, and chronic HBV infection (Fig. 2C). Comparing the strength of HBV-specific CD8+ T-cell responses targeting different HBV antigens, we found no significant differences (Fig. 2D).

Resolution of chronic HBV infection represents a rare event.^19^ To complement our observations in acute, resolved, and chronic infection, we screened a small number of patients (n = 4) after resolution of chronic infection for their HBV-specific CD8+ T-cell repertoire (Tables S1 and S2). Two of four patients showed at least one HBV-specific CD8+ T-cell response. Interestingly, the overall three HBV-specific CD8+ T-cell responses were located in polymerase and X protein, and targeted new epitopes. Importantly, similar to the findings in our cohort with chronic HBV infection, none of the responses was located in HBsAg.

### Dominance of conserved HLA-B-restricted CD8+ T-cell epitopes in chronic HBV infection

**Fig. 3:**
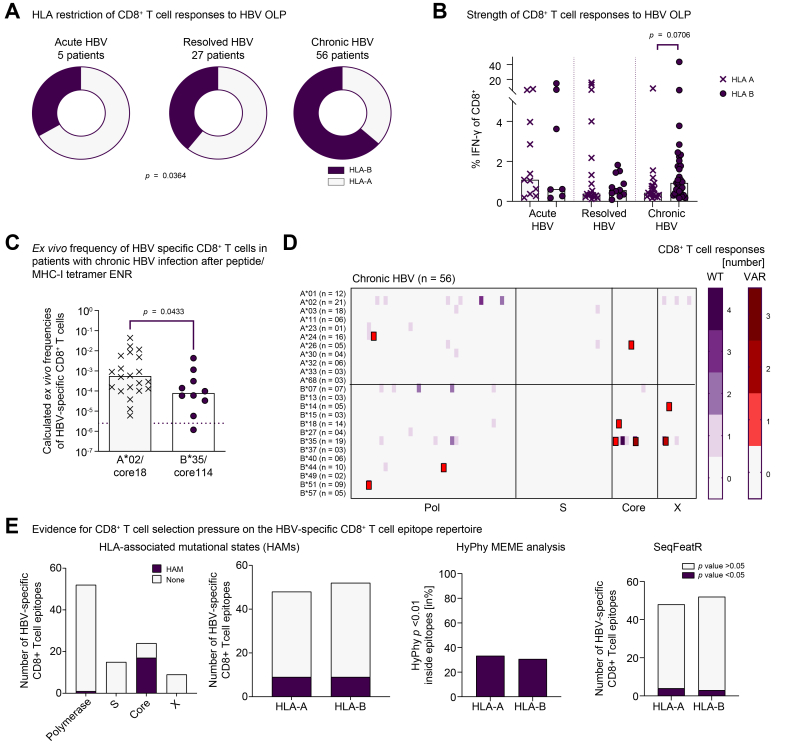
Dominance of HLA-B-restricted CD8+ T-cell epitopes in chronic HBV infection. (A) Percentage of HBV-specific CD8+ T-cell responses restricted by HLA-A *vs.* HLA-B in acute, resolved, or chronic HBV infection. Statistical analysis was performed with Fisher’s exact test. (B) Strength of HLA-A- *vs.* HLA-B-restricted HBV-specific CD8+ T-cell responses to HBV OLPs. Statistical analysis was performed with Kruskal-Wallis test with false-discovery rate correction using a step-up procedure of Benjamini, Krieger, and Yekutieli. (C) Calculated *ex vivo* frequencies of HBV-specific CD8+ T cells targeting a representative HLA-A *vs.* HLA-B-restricted core-epitope in patients with chronic HBV infection. Statistical analysis was performed with two-tailed Mann-Whitney *U* test. (D) Number, location, and HLA restriction of HBV-specific CD8+ T-cell responses to OLPs spanning the whole HBV proteome in patients with chronic HBV infection. Epitopes with sequence variations within the autologous viral sequences from the respective patients are indicated in red. (E) Number of HBV-specific CD8+ T-cell epitopes with or without HLA-associated mutational states (HAMdetector) or with *vs.* without positions under T-cell selection pressure (HyPhy MEME analysis and SeqFeatR). Statistical analysis was performed with two-way ANOVA with false-discovery rate correction for multiple comparisons using a step-up procedure of Benjamini, Krieger, and Yekutieli or unpaired *t* test. HLA, human leukocyte antigen; MEME, mixed effects model of evolution; OLPs, overlapping peptides; ENR, enrichment.

Regarding HLA restriction, patients with chronic HBV infection showed more HLA-B-restricted CD8+ T-cell responses, whereas the distribution in patients with acute/resolved HBV infection was quite balanced (Fig. 3A). This was also true when analyzing the data per patient with a significant (*p* = 0.0318) enrichment of patients targeting only HLA-B-restricted CD8+ T-cell responses in patients with chronic HBV infection compared with the combined cohorts with acute/resolved HBV infection (Fig. S3A). In patients with chronic HBV infection, HLA-B-restricted CD8+ T-cell responses were restricted by overall seven HLA types. HLA-B∗07 and HLA-B∗35 were the most frequently detected HLA restrictions. In patients with acute/resolved HBV infection, HLA-B-restricted CD8+ T-cell responses were restricted overall by eight HLA types; HLA-B∗15, HLA-B∗35, and HLA-B∗40 were the most frequently detected HLA restrictions (Fig. S3B). Importantly, there was no significant enrichment of HLA-B∗07 or HLA-B∗35 in patients with chronic HBV infection (Fig. S1B). To confirm the dominance of the identified HLA-B-restricted CD8+ T-cell epitopes, we tested them in additional HLA-matched patients with chronic HBV infection after peptide-specific expansion with the minimal optimal epitope (Fig. S3C). The epitopes were dominant, with 12 of 20 HLA-B-restricted epitopes targeted in >20% of patients and seven of 20 epitopes in >40% of patients (Fig. S3C). Notably, similar to the findings obtained with OLPs, HLA-B∗35 was the most frequently detected HLA restriction, followed by HLA-B∗07. In patients with chronic HBV infection, there was a trend towards more vigorous HLA-B- *vs.* HLA-A-restricted CD8+ T-cell responses; however, this trend was not statistically significant (Fig 3B). Of note, both HLA-A and HLA-B-restricted epitopes reached detectable (>0.1%) IFN-γ responses in titration experiments at the applied peptide concentration of 5 μM (Fig. S3D). Comparison of *ex vivo* frequencies of HBV-specific CD8+ T cells targeting a representative HLA-A- *vs.* HLA-B-restricted epitope, interestingly, revealed lower frequencies of CD8+ T cells targeting the HLA-B- compared with the HLA-A-restricted epitope (Fig. 3C). This indicates that the dominance of functional HLA-B-restricted CD8+ T-cell epitopes in chronic HBV infection might not simply be explained by higher *ex vivo* frequencies.

Next, we analyzed the autologous viral sequences corresponding to CD8+ T-cell epitopes in patients with chronic HBV infection and a detectable immune response (Table S2). We found viral sequence variations in overall 11 of 54 (20.4%) HBV-specific CD8+ T-cell responses (Fig. 3D). Of these 11 variants, seven were previously described as escape variants on the basis of footprint analysis.^7^ In two exemplary cases, viral escape was also experimentally demonstrated (Fig. S3E). The autologous variant of epitope B∗14/X_45-53_ “VS**SGL**GAHL” in patient CH-13 was no longer recognized by patient’s CD8+ T cells. The autologous variant of epitope B∗35/X_10-18_ “D**T**ARDVLCL” in patient CH-3 and CH-24 was cross-recognized. However, because the mutation affects the HLA-binding motif of the epitope, we pulsed B-LCL matched for only B∗35 with the respective patients with wild type and variant of epitope B∗35/X_10-18_. Indeed, the B-LCL pulsed with variant peptide prompted a weaker HBV-specific CD8+ T-cell response upon co-culture with patient’s PBMCs, indicative of suboptimal binding of the variant to the HLA molecule as escape mechanism (Fig. S3E). There was no significant accumulation of sequence variations in HLA-B-restricted CD8+ T-cell responses compared with HLA-A-restricted responses. We then expanded the analysis to the HBV-specific CD8+ T-cell epitope repertoire identified across all cohorts and used a set of 239 previously published sequences of patients with known HLA alleles (all HBV genotype D; all HBeAg negative). All HBV-specific CD8+ T-cell epitopes targeted in our study were screened with a Bayesian model (HAMdetector) for identification of HLA-associated mutational states (HAMs).^8^ The posterior probabilities for substitutions being HLA associated were quantified for each position in the targeted epitope in a range from 0 (“substitution is disfavored in the presence of the restricting HLA allele”) to 1 (“substitution is favored in the presence of the restricting HLA allele”). High posterior probabilities with values >0.8 indicate that the amino acid is favored in individuals with this HLA class I allele, consistent with CD8 T-cell escape. For each epitope, the maximum posterior probability was determined and epitopes with at least one residue >0.8 were considered “HAM positive.” In line with recent evidence,^7^ most HAMs were located in the HBV core protein. The number of HAM-positive epitopes was similar in HLA-A- *vs.* HLA-B-restricted epitopes (Fig. 3E). This was also true when analyzing viral selection pressure in HLA-A- *vs*. HLA-B-restricted epitopes using alternative statistical methods (MEME analysis and SeqFeatR). Accordingly, general entropy analysis did not find differences in sequence variability in HLA-A- *vs.* HLA-B-restricted epitopes (Fig. S3F). Overall, the dominance of HLA-B-restricted HBV-specific CD8+ T-cell epitopes is not associated with T-cell selection pressure, making these epitopes interesting candidates for immunotherapeutic approaches.

## Discussion

Our data provides an overview of the HBV-specific CD8+ T-cell landscape in different stages of HBV infection. The main finding is that the functional HBV-specific CD8+ T-cell epitope repertoire in chronic infection differs from that in acute/resolved HBV infection in several important aspects. Although our experimental setup is focused on functional repertoire and competitive effects, and the necessity for epitope processing from OLPs may limit the complete detection of all CD8+ T-cell responses, our data clearly supports the following conclusions:

(1) The antigen target of functional HBV-specific CD8+ T-cell responses is different in acute/resolved *vs.* chronic HBV infection. Indeed, HBsAg-specific CD8+ T-cell responses are largely absent in chronic HBeAg-negative HBV infection. In addition, polymerase-specific as well as X protein-specific CD8+ T-cell responses are enriched in chronic HBV infection (trend). This observation underlines the previous notion that the functional HBV-specific CD8+ T-cell epitope repertoire differs between acute/resolved *vs.* chronic infection. Le Bert *et al.*^18^ observed, for example, rarely HBsAg-specific CD8+ T-cell responses in patients with chronic HBV infection aged >30 yr. This finding is in line with a low detection rate of HBsAg-specific CD8+ T cells by our group, even when using a highly sensitive tetramer-based enrichment approach.^20^ Park *et al.*^21^ also found a different distribution of HBV-specific CD8+ T-cell responses to HBV proteins in different clinical phases. The mechanisms resulting in the differential antigen targeting in chronic *vs.* acute/resolved HBV infection are not yet well defined. Long-term exposure to high antigen loads may contribute to exhaustion and deletion of HBsAg-specific CD8+ T cells. For the other HBV antigens, a growing body of evidence indicates that the targeted antigen may have an important impact on function and differentiation of HBV-specific CD8+ T cells.^5^^,^^20^^,^[22], [23], [24] Indeed, HBV-specific CD8+ T cells targeting different viral antigens are a heterogeneous population composed of subsets with different functional levels contributing to endogenous viral control in some patients. Although our results agree with previous reports testing responses to OLP pools, the small number of available samples with acute HBV infection and combination of acute and resolved cohorts for a few analyses is a limitation of our dataset. For patients with resolved HBV infection, time since viral clearance might also influence the vigor of detected CD8+ T-cell responses.^13^ Unfortunately, precise clinical information on the time point of viral clearance/infection is only available for a small subgroup of our cohort with resolved HBV infection, hindering systematic stratified analysis.
(2) In patients with HBeAg-negative chronic HBV infection, the majority of detectable IFN-γ-producing HBV-specific CD8+ T-cell responses were restricted by HLA-B, whereas the majority of HBV-specific CD8+ T-cell responses in patients with acute/resolved HBV infection were restricted by HLA-A. Notably, the dominance of HLA-B-restricted CD8+ T-cell responses was largely driven by the common HLA-B types B∗07 and B∗35 and not limited to rare HLA-B types. This dominance of HLA-B does not translate into higher frequencies of HLA-B-associated mutational escape, as viral sequence variations occurred with similar frequency in both HLA-A- and HLA-B-restricted epitopes. This was true in our cohort with comprehensive data on the targeted epitopes as well as in a larger cohort with combined viral sequence and HLA data. To our knowledge, our study is the first to report the dominance of HLA-B-restricted CD8+ T-cell responses in chronic HBV infection. This observation further supports the concept of differential HBV-specific CD8+ T-cell epitope repertoires in acute/resolved *vs.* chronic HBV infection. This observation agrees with findings in the context of other chronic viral infections such as HCV^25^^,^^26^ and HIV,^27^ where these responses are associated with viral control. Viral sequence variations consistent with viral escape occurred in 20% of targeted HBV-specific CD8+ T-cell epitopes. This is less frequent compared with, for example, HCV infection, where ∼50–70% of virus-specific CD8+ T-cell responses are affected by viral escape.^28^^,^^29^ This finding is consistent with previous concepts suggesting that viral escape in HBV infection is limited by overlapping reading frames of HBV proteins, thereby limiting mutational flexibility of HBV.^30^ Of note, this finding may not necessarily translate to a setting of more replicative chronic HBV infection, where viral escape may occur more frequently. Data on this important issue are still lacking. However, in contrast to HCV and HIV, HLA-B does not seem to have a dominant role in driving viral evolution in HBV infection. HLA-B-restricted epitopes in chronic HBV infection might be more under constraint to retain their viral sequence due to overlapping reading frames. HLA-B-restricted HLA-binding patterns more often include amino acids with structural function (*e.g.* proline at position 2 for HLA-B∗07 supertype, arginine at position 2 for HLA-B∗27), whereas HLA-A-restricted epitopes generally allow more flexible amino acid residues at their binding anchors (*e.g.* HLA-A∗02 supertype requires a small or aliphatic hydrophobic residue [A, I, V, L, M, or T] at the second amino acid position). The substitution of amino acids within HLA-B binding patterns therefore might be associated with higher viral fitness costs in the context of overlapping reading frames. Thus, it might be worthwhile to include these HLA-B-restricted epitopes as targets in immunotherapeutic approaches such as therapeutic vaccination. The mechanisms that may direct the dominance of HLA-B restriction in chronic HBV infection remain elusive. As the proportion of responses affected by viral escape mutations does not substantially differ between HLA-A and HLA-B, superior functional capacities of CD8+ T cells that no longer recognize their cognate epitope due to mutational escape and are less prone to T-cell exhaustion is unlikely to explain the dominance of HLA-B-restricted CD8+ T-cell responses. Of note, the abundance of HLA-B-restricted CD8+ T-cell responses in chronic HBV infection may be owing to the sensitivity of the method used and possible competitive effects, as there was a trend towards more vigorous HLA-B- than HLA-A-restricted CD8+ T-cell responses in chronic HBV infection but not in acute/resolved HBV infection. However, the dominance of functional HLA-B-restricted CD8+ T-cell epitopes in chronic HBV infection might not be explained simply by higher *ex vivo* frequencies (Fig. 3C). Further longitudinal studies need to clarify if the difference in HLA restriction of the functional HBV-specific CD8+ T-cell repertoire between chronic and acute/resolved HBV infection is dynamic (*e.g.* shifting in patients with acute-persisting infection from acute to chronic infection, or if patients with acute-resolving *vs.* chronic-persisting HBV infection primarily show a differential HBV-specific CD8+ T-cell epitope repertoire already in the acute phase of infection). Future studies also need to clarify if HLA-B- *vs.* HLA-A-restricted responses differ in cytotoxic capacity, proliferative potential, and phenotype.
(3) Consistent with a differential antigen targeting and HLA restriction, the functional HBV-specific CD8+ T-cell epitope repertoire in chronic HBV infection is not well covered by previously described HBV-specific CD8+ T-cell epitopes. These epitopes have been mostly identified in patients with acute/resolved HBV infection. This finding indicates that utilizing the previously described HBV-specific CD8+ T-cell epitopes from databases in immunology studies leads to a bias when analyzing the HBV-specific CD8+ T-cell epitope repertoire in chronic HBV infection. Furthermore, using these known HBV-specific CD8+ T-cell epitopes in immunotherapeutic approaches aiming at functional cure of chronic HBV infection may not be appropriate. We thus aimed to add to the knowledge of HBV-specific CD8+ T-cell epitopes that are targeted in chronic HBV infection. Importantly, we could fine-map 28 novel HBV-specific CD8+ T-cell epitopes that were targeted in chronic HBV infection. When defining the HLA restriction of these novel epitopes, in line with our previous observations, HLA-B clearly dominated. These novel epitopes may be used as a “toolbox” for further studies and also for target antigen/epitope design in the context of immunotherapeutic approaches. Utilizing the previously described HBV-specific CD8+ T-cell epitopes from databases in immunology studies instead might lead to a bias when analyzing the HBV-specific CD8+ T-cell epitope repertoire in chronic HBV infection. In addition, T-cell receptor-redirected CD8+ T cells targeting the novel epitopes in the X protein may be promising for the treatment of HBV-associated hepatocellular carcinoma.^31^

In this study, we demonstrated a differential epitope repertoire of acute and resolved *vs.* chronic HBV infection with respect to viral antigen target and HLA-A *vs.* HLA-B dominance. The HBV-specific CD8+ T-cell epitope repertoire in chronic HBV infection is not well covered by previously described HBV-specific CD8+ T-cell epitopes. These findings have important implications for the design of immunotherapeutic approaches towards functional cure of chronic HBV infection. Indeed, targeting the well-conserved HLA-B-restricted CD8+ T-cell epitopes by therapeutic vaccination, in combination with restoration and enhancement of pre-existing CD4+ and CD8+ T-cell responses,[32], [33], [34] may be an important therapeutic component for HBV infection.

## Abbreviations

B-LCL, B-lymphoblastoid cell line; HAM, HLA-associated mutational state; HLA, human leukocyte antigen; ICCS, intracellular cytokine staining; IEDB, Immune Epitope Database; IFN-γ, interferon gamma; MEME, mixed effects model of evolution; OLPs, overlapping peptides; PBMCs, peripheral blood mononuclear cells.

## Authors’ contributions

JLM, ALD, JP, PE, ESA, HL, MM, MK, FJ, and IS planned, performed, and analyzed experiments. GR, EG, SLL, FE, BB, TB, DAP, AW, and JT collected clinical data and provided critical reagents/resources. RT, MH, and CNH designed the study and contributed to experimental design and planning. JLM, RT, MH, and CNH. interpreted data and wrote the manuscript. All authors contributed intellectually and concurred with the decision to submit the work for publication.

## Data availability

No data is deposited on public databases. All requests for raw and analyzed data and materials will be reviewed by the corresponding authors to verify if the request is subject to any confidentiality obligations. Patient-related data not included in the paper were generated as part of clinical examination and may be subject to patient confidentiality. Any data and materials that can be shared will be released via a material transfer agreement.

## Financial support

This study was supported by grants from the 10.13039/501100001659Deutsche Forschungsgemeinschaft (DFG, 10.13039/501100001659German Research Foundation; 272983813 to BB, TB, RT, MH, and CNH, and 256073931 to BB, RT, MH, and CNH; IMM-PACT Program for Clinician Scientists 413517907 to HL and JLM) and the 10.13039/100009139Deutsches Zentrum für Infektionsforschung (DZIF, 10.13039/100009139German Center for Infection Research; TTU Hepatitis to TB, RT, and CNH). JLM was further supported by an Else Kröner Memorial Fellowship from the 10.13039/501100003042Else Kroner-Fresenius Foundation. HL was further supported by the IMMediate Advanced Clinician Scientist-Program, Department of Medicine II, Medical Center – 10.13039/501100002714University of Freiburg and 10.13039/501100021729Faculty of Medicine, University of Freiburg, funded by the 10.13039/501100002347Bundesministerium für Bildung und Forschung (BMBF, 10.13039/501100002347Federal Ministry of Education and Research) - 01EO2103. MH was further supported by the Heisenberg program (DFG, 10.13039/501100001659German Research Foundation; HO 5836/2-1). RT was further supported by the European Union (EU H2020-847939-IP-cure-B). JP was supported by the German Federal Ministry of Education and Research (10.13039/501100002347Bundesministerium für Bildung und Forschung; Netzwerk Universitätsmedizin, GenSurv/MolTraX 01KX2021). DAP was supported by a Wellcome Trust Senior Investigator Award (100326/Z/12/Z). The funding bodies had no role in the decision to write or submit the manuscript for publication.

## Conflicts of interest

Please refer to the accompanying ICMJE disclosure forms for further details.
