# Supplementary material for: Robust HLA-B-restricted CD8+ T-cell responses in chronic HBV infection
Source: JHEP Rep. 2026 Apr 24;8(8):101868. doi: 10.1016/j.jhepr.2026.101868 (PMC13351134; doi:10.1016/j.jhepr.2026.101868)
Supplement: Multimedia component 1 [file mmc1.pdf]

Robust HLA-B restricted CD8+ T cell responses in chronic HBV infection

Julia Lang-Meli, Anna-Lena Denecke, Johannes Ptok, Philipp Ehrenmann, Elahe Salimi Alizei, Hendrik Luxenburger, Michelle Maas, Muthamia Kiraithe, Felix Jacobi, Giuseppe Rusignuolo, Isabel Schulien, Emma Gostick, Sian Llewellyn-Lacey, Florian Emmerich, Bertram Bengsch, Tobias Böttler, David A. Price, Andreas Walker, Jörg Timm, Robert Thimme, Maike Hofmann, Christoph Neumann-Haefelin

Table of contents

Fig. S1 ……………………………………………………………...………………...2

Fig. S2 ……………………………………………………………………………..…3

Fig. S3 ………………………………………………………………………...……...4

Fig. S4 ……………………………………………..…………………………………6

Table S1 …………………………………………………..……………………….……7

Table S2 ……………………………………………..………………………..……..12

Table S3 …………………………………….……………………………….………...33

Table S4 …………………………………………………………………………………37

Table S5 …………………………………………………...…………….………..38

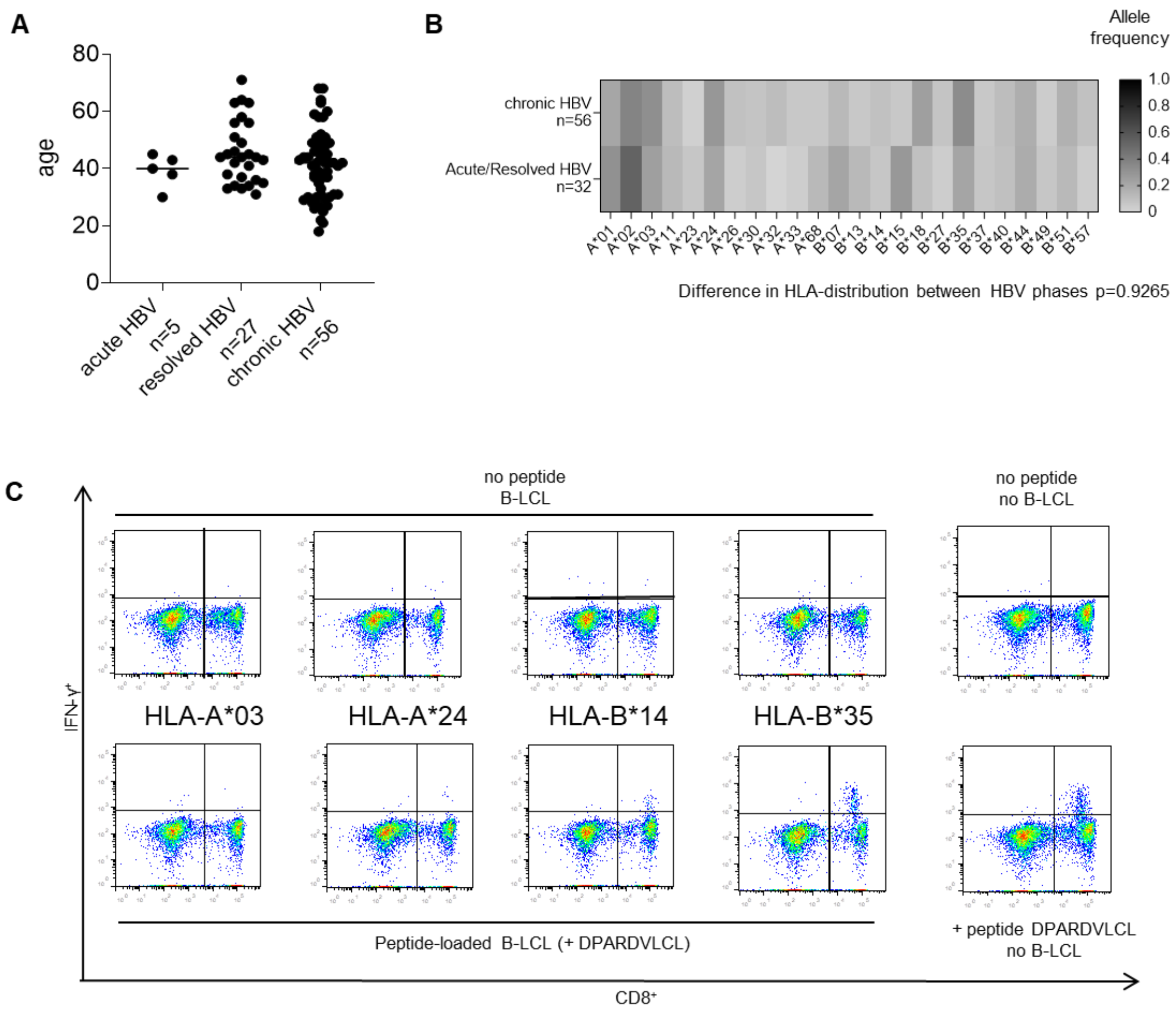

Fig. S1. Patient cohorts and representative staining

Age of patients (A) and frequency of different HLA types (B) in the patient cohorts. Representative staining for experimental determination of HLA-restriction using B-lymphoblastoid cell lines (B-LCL); patient CH-3 with response to B*35-restricted epitope DPARDVLCL (C). Statistical analysis was performed with Kruskal-Wallis test (A) or two-way ANOVA (B) with false-discovery rate correction using a step-up procedure of Benjamini, Krieger and Yekutieli.

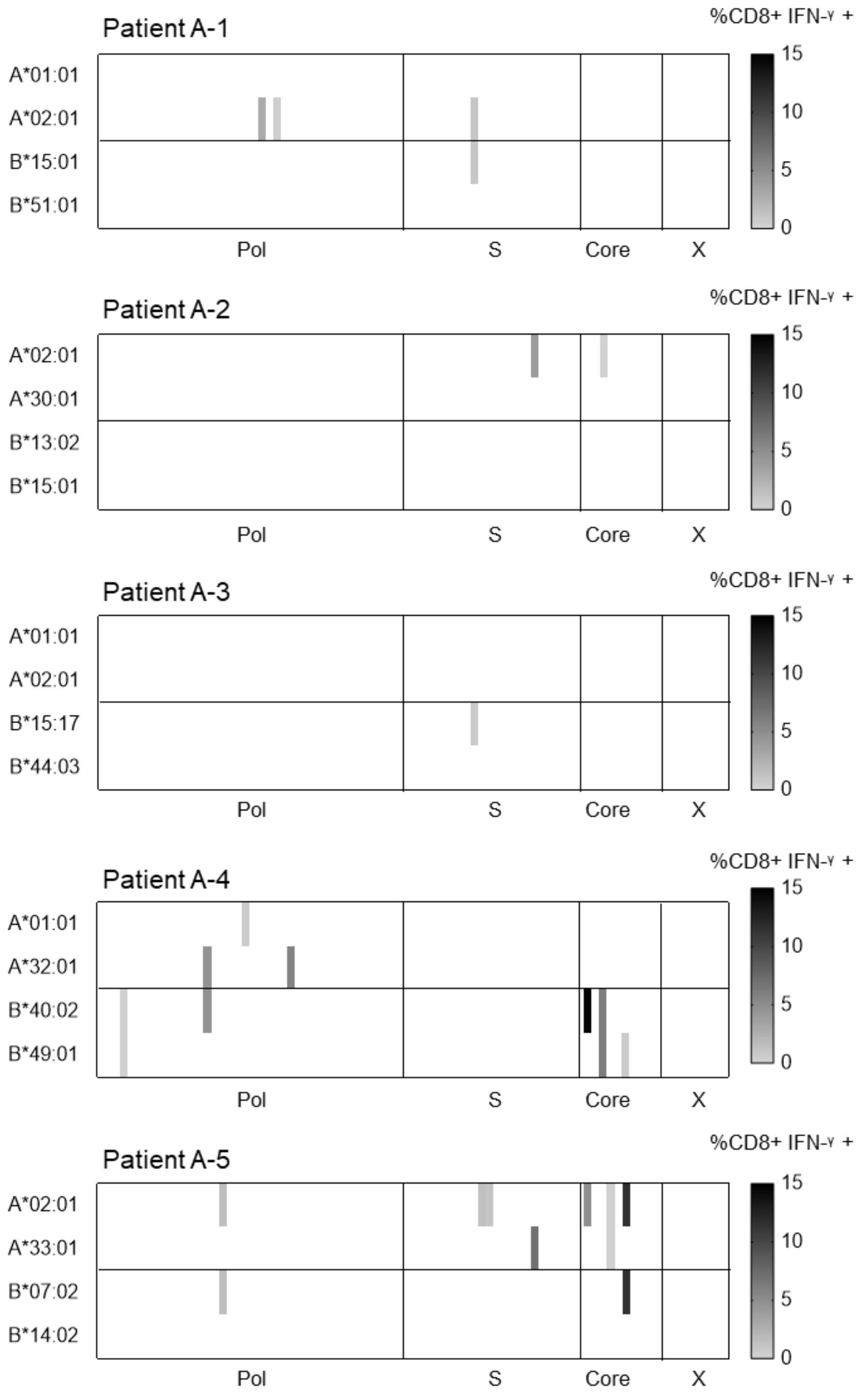

Fig. S2. HBV-specific CD8+ T cell repertoire in individual patients with acute HBV infection

Strength, location and HLA-restriction of HBV-specific CD8+ T cell responses to OLP spanning the whole HBV proteome in 5 patients with acute HBV infection

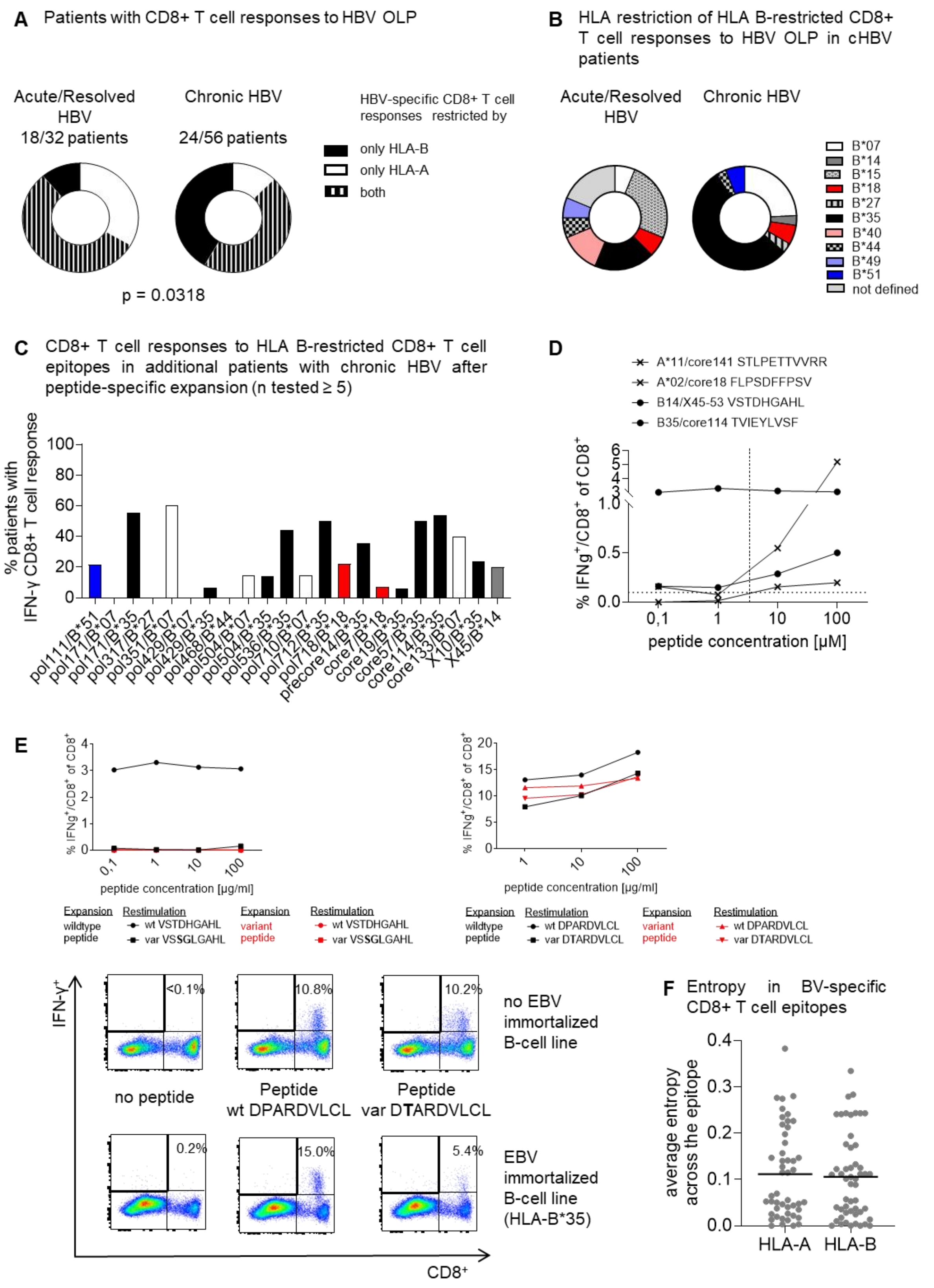

Fig. S3. Dominant HLA-B-restricted HBV-specific CD8+ T cell epitopes

18/32 patients with acute/resolved, and 24/56 patients with chronic HBV infection targeted at least one HBV OLP. Of these, the number of patients targeting only HLA-

A, HLA-B restricted epitopes or both are depicted; statistical analysis was performed with Fisher’s exact test (A). HLA type of HLA-B-restricted HBV-specific CD8+ T cell responses to OLP in the patient cohort with chronic (n=56) or acute/resolved (n=32) HBV infection (B). Testing of dominant HLA-B-restricted HBV-specific CD8+ T cell epitopes in additional HLA-matched patients with chronic HBV infection after peptide-specific expansion with the minimal optimal epitope (C). Peptide titrations after peptide-specific expansion of PBMC using representative HLA-A versus HLA-B restricted epitopes. The peptide concentration used in our study (5µM) and the cutoff for HBV-specific CD8+ T cell responses (0.1% IFNg+/CD8+ of CD8+) are indicated by dotted lines (D). Testing of representative wildtype versus autologous variant epitopes by peptide titration are depicted. Loading of wildtype versus variant of epitope B*35/$_{X10\text{-}18}$ using B-lymphoblastoid cell lines (B-LCL) is shown (E). Entropy (sequence variability) in HBV-specific CD8+ T cell epitopes targeted in our study. Statistical analysis was performed with unpaired t-test (F)

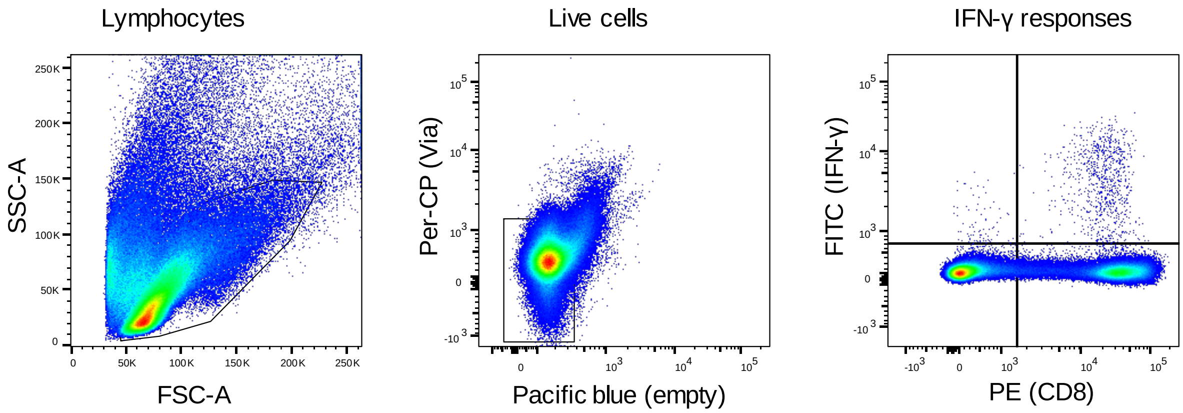

Fig. S4. Gating strategy

The gating strategy is depicted.

Table S1 Patient characteristics

Clinical parameters in the tested patient cohorts (acute, resolved and chronic HBV infection). NA = not available; NUC = nucleoside-analogues; IFN = interferon

code	HLA type	sex	age	cohort	acute/ resolved: details on clinical history	chronic: EASL clinical stage	chronic: NUC-therapy?	chronic: history of IFN treatment?	Viral load [IU/ml]	ALT [U/L]
CH-1	A*02:01, A*24:02, B*18:01, B*39:01	M	68	chronic		HBeAg negative chronic hepatitis	yes	no	<10	23
CH-2	A*02:01, A*03:01, B*35:03, B*51:01	M	49	chronic		HBeAg negative chronic hepatitis	yes	no	<10	16
CH-3	A*03:01, A*24:02, B*14:02, B*35:02	M	49	chronic		HBeAg negative chronic hepatitis	yes	yes	74	53
CH-4	A*02:01, A*24:02, B*08:01, B*1801	F	39	chronic		HBeAg negative chronic infection	no	no	962	35
CH-5	A*30:02, A*68:01, B*18:01, B*40:01	M	41	chronic		HBeAg negative chronic hepatitis	yes	no	<10	99
CH-6	A*02:01, A*24:02, B*15:01, B*1801	F	49	chronic		HBeAg negative chronic hepatitis	yes	no	<10	28
CH-7	A*01:01, A*30:01, B*35:08, B*51:01	F	26	chronic		HBeAg negative chronic infection	no	no	137	27
CH-8	A*11:01, A*24:02, B*18:01, B*37:01	M	31	chronic		HBeAg negative chronic hepatitis	yes	yes	<10	44
CH-9	A*02:01, A*03:01, B*18:01, B*4901	M	43	chronic		HBeAg negative chronic hepatitis	no	yes	10209	28
CH-10	A*02:01, A*32:01, B*08:01, B*35:03	F	42	chronic		HBeAg negative chronic infection	no	no	919	30
CH-11	A*01:01, A*24:02, B*35:03, B*44:02	M	31	chronic		HBeAg negative chronic infection	no	no	7149	23
CH-12	A*24:02, A*32:01, B*18:01, B*40:02	F	18	chronic		HBeAg negative chronic infection	no	no	2462	27
CH-13	A*24:02, A*33:01, B*14:02, B*49:01	F	58	chronic		HBeAg negative chronic infection	no	no	3871	30
CH-14	A*03:01, A*24:02, B*15:01, B*35:03	M	44	chronic		HBeAg negative chronic infection	no	no	1590	23
CH-15	A*11:01, A*24:02, B*27:02, B*44:02	M	21	chronic		HBeAg negative chronic hepatitis	yes	no	<10	49
CH-16	A*02:01, A*03:01, B*44:03, B*44:05	F	59	chronic		HBeAg negative chronic infection	no	no	2092	24

CH-17	A*02:01, A*23:01, B*07:05, B*44:03	F	42	chronic		HBeAg negative chronic infection	no	no	120	23
CH-18	A*33:01, B*14:02	F	46	chronic		HBeAg negative chronic infection	no	no	30	42
CH-19	A*03:01, A*68:02, B*27:02, B*38:01	M	33	chronic		HBeAg negative chronic hepatitis	yes	yes	<10	26
CH-20	A26:01, A*31:01, B*07:02, B*51:01	M	42	chronic		HBeAg negative chronic hepatitis	yes	no	17	71
CH-21	A*24:02, A*66:01, B*41:02, B*51:01	F	58	chronic		HBeAg negative chronic hepatitis	no	no	29625	64
CH-22	A*03:01, A*11:01, B*35:01, B*37:01	F	42	chronic		HBeAg negative chronic hepatitis	yes	no	<10	23
CH-23	A*01:01, A*02:05, B*44:02, B*51:01	M	42	chronic		HBeAg negative chronic infection	no	no	14335	34
CH-24	A*01:01, A*11:01, B*18:01, B*35:02	F	30	chronic		HBeAg negative chronic hepatitis	no	no	21727	41
CH-25	A*01:01, A*32:01, B*08:01	M	27	chronic		HBeAg negative chronic infection	no	no	4076	39
CH-26	A*01:01, A*03:01, B*37:01, B*51:01	M	30	chronic		HBeAg negative chronic hepatitis	no	no	4853	53
CH-27	A*01:01, A*03:01, B*13:02, B*35:01	M	45	chronic		HBeAg negative chronic hepatitis	yes	no	<10	33
CH-28	A*26:01, A*30:01, B*13:02, B*35:01	F	28	chronic		HBeAg negative chronic hepatitis	yes	yes	<10	48
CH-29	A*02:01, B*35:01, B*40:01	F	27	chronic		HBeAg negative chronic hepatitis	yes	no	20	30
CH-30	A*02:01, A*25:01, B*14:02, B*18:01	F	31	chronic		HBeAg negative chronic hepatitis	yes	no	<10	51
CH-31	A*03:01, A*2402, B*35:01	F	51	chronic		HBeAg negative chronic hepatitis	yes	no	87	20
CH-32	A*03:01, A*25:01, B*07:02, B*57:01	F	29	chronic		HBeAg negative chronic infection	no	no	456	12
CH-33	A*01:01, A*66:01, B*41:02, B*57:01	M	37	chronic		HBeAg negative chronic infection	no	no	1339	56
CH-34	A*03:01, A*31:01, B*35:01, B*57:01	F	41	chronic		HBeAg negative chronic infection	no	no	976	23
CH-35	A*25:01, A*32:01, B*18:01, B*57:01	M	35	chronic		HBeAg negative chronic infection	no	no	1160	42
CH-36	A*01:01, A*02:01, B*40:06, B*41:01	F	46	chronic		HBeAg negative chronic hepatitis	no	no	828919	46
CH-37	A*02:01, A*31:01, B*15:01, B*40:01	M	39	chronic		HBeAg negative chronic infection	no	no	347	30

CH-38	A*02:01, A*74:03, B*18:01, B*44:03	F	30	chronic		HBeAg negative chronic infection	no	no	288	28
CH-39	A*02:01, A*24:02, B*07:02, B*44:03	F	44	chronic		HBeAg negative chronic infection	no	no	13524	26
CH-40	A*01:01, A*02:01, B*07:02, B*18:01	M	52	chronic		HBeAg negative chronic hepatitis	yes	no	101	59
CH-41	A*24:02, A*32:01, B*35:03, B*35:08	F	49	chronic		HBeAg negative chronic infection	no	no	2220	21
CH-42	A*11:01, A*24:02, B*07:02, B*35:01	F	25	chronic		HBeAg negative chronic infection	no	no	15	34
CH-43	A*24:02, B*35:02	F	29	chronic		HBeAg negative chronic hepatitis	yes	no	11	40
CH-44	A*01:01, A*26:01, B*08:01, B*38:01	M	63	chronic		HBeAg negative chronic hepatitis	yes	no	<10	57
CH-45	A*03:01, A*03:02, B*44:02, B*51:01	M	44	chronic		HBeAg negative chronic hepatitis	yes	no	11	38
CH-46	A*03:01, A*32:01, B*13:02, B*40:02	F	68	chronic		HBeAg negative chronic hepatitis	yes	no	<10	38
CH-47	A*02:01, A*33:01, B*14:02, B*18:01	M	51	chronic		HBeAg negative chronic hepatitis	yes	no	<10	72
CH-48	A*03:02, A*11:01, B*35:01, B*41:02	M	22	chronic		HBeAg negative chronic infection	no	no	2399	27
CH-49	A*01:01, A*02:01, B*35:03, B*51:01	M	38	chronic		HBeAg negative chronic hepatitis	yes	no	<10	30
CH-50	A*30:01, B*41:01	F	37	chronic		HBeAg negative chronic hepatitis	yes	no	23764	64
CH-51	A*26:01, B*27:05, B*51:01	F	38	chronic		HBeAg negative chronic infection	no	no	117	46
CH-52	A*03:01, A*68:01, B*27:02, B*57:01	F	60	chronic		HBeAg negative chronic hepatitis	yes	no	273	19
CH-53	A*02:01, B*35:01, B*52:01	M	64	chronic		HBeAg negative chronic hepatitis	yes	no	871	28
CH-54	A*02:01, B*18:01, B*44:02	F	48	chronic		HBeAg negative chronic infection	no	no	1795	31
Ch-55	A*02:05, A*03:01, B*44:03, B*50:01	F	47	chronic		HBeAg negative chronic hepatitis	no	no	22616	50
CH-56	A*03:01, A*26:01, B*07:02, B*41:01	M	38	chronic		HBeAg negative chronic hepatitis	yes	no	3449	40
R-1	A*01:01, A*02:01, B*14:01, B*38:01	F	43	resolved	incidental serological finding of resolved HBV infection at blood donation center (anti-HBs positive, anti-HBc positive)				not applicable	68
R-2	A*02 (flow cytometry); 4-digit HLA typing NA	F	46	resolved	incidental serological finding of resolved HBV infection at check-up (anti-HBs positive, anti-HBc positive)				not applicable	29

R-3	A*01:01, A*11:01, B*40:06, B*44:03	M	51	resolved	serological finding of resolved HBV infection (anti-HBs positive, anti-HBc positive), check up because of partner with HBV				not applicable	54
R-4	A*01:01, A*02:01, B*35:01, B*51:01	F	33	resolved	incidental serological finding of resolved HBV infection at check-up (anti-HBs positive, anti-HBc positive)				not applicable	48
R-5	A*03:01, A*68:01, B*35:02, B*39:01	F	45	resolved	incidental serological finding of resolved HBV infection at check-up (anti-HBs negative, anti-HBc positive, HBsAg negative)				not applicable	34
R-6	A*03:01, A*26:01, B*15:17, B*38:01	F	44	resolved	incidental serological finding of resolved HBV infection at blood donation center (anti-HBs positive, anti-HBc positive)				not applicable	13
R-7	A*23:01, A*68:01, B*44:03, B*51:01	M	33	resolved	serological finding of resolved HBV infection (anti-HBs positive, anti-HBc positive), check up because of partner with HBV				not applicable	26
R-8	A*02:01, B*35:02, B*57:01	M	45	resolved	documented acute HBV infection with spontaneous resolution 8 years ago				not applicable	35
R-9	A*01:01, A*11:01, B*37:02, B*52:01	F	42	resolved	incidental serological finding of resolved HBV infection at check-up (anti-HBs positive, anti-HBc positive)				not applicable	22
R-10	A*03:01, A*23:01, B*07:02, B*44:03	F	44	resolved	documented acute HBV infection with spontaneous resolution 1 year ago				not applicable	43
R-11	A*02:01, A*11:01, B*15:02, B*18:01	M	31	resolved	incidental serological finding of resolved HBV infection at check-up (anti-HBs positive, anti-HBc positive)				not applicable	50
R-12	A*03:01, A*24:02, B*27:05, B*52:01	F	58	resolved	incidental serological finding of resolved HBV infection at check-up (anti-HBs positive, anti-HBc positive)				not applicable	40
R-13	A*01:01, A*24:02, B*35:02, B*38:01	M	44	resolved	incidental serological finding of resolved HBV infection at check-up (anti-HBs negative, anti-HBc positive, HBsAg negative)				not applicable	21
R-14	A*03:01, A*68:01, B*07:02, B*35:03	M	41	resolved	incidental serological finding of resolved HBV infection at check-up (anti-HBs positive, anti-HBc positive)				not applicable	27
R-15	A*01:01, A*24:02, B*07:02, B*37:01	M	56	resolved	incidental serological finding of resolved HBV infection at blood donation center (anti-HBs positive, anti-HBc positive)				not applicable	59
R-16	A*03:01, A*24:02, B*07:02, B*35:01	F	34	resolved	incidental serological finding of resolved HBV infection at check-up (anti-HBs positive, anti-HBc positive)				not applicable	7
R-17	A*02:01, B*13:02, B*44:03	F	34	resolved	incidental serological finding of resolved HBV infection at check-up (anti-HBs positive, anti-HBc positive)				not applicable	16
R-18	A*24:02, A*24:10, B*15:02, B*48:03	F	38	resolved	incidental serological finding of resolved HBV infection at check-up (anti-HBs positive, anti-HBc positive)				not applicable	17
R-19	A*11:01, A*24:02, B*13:02, B*52:01	F	35	resolved	incidental serological finding of resolved HBV infection at check-up (anti-HBs negative, anti-HBc positive, HBsAg negative)				not applicable	30
R-20	A*01:01, A*02:01, B*08:01, B*18:01	F	71	resolved	incidental serological finding of resolved HBV infection at check-up (anti-HBs positive, anti-HBc positive)				not applicable	32
R-21	A*68:01, B*15:01, B*35:03	F	63	resolved	incidental serological finding of resolved HBV infection at check-up (anti-HBs positive, anti-HBc positive)				not applicable	41
R-22	A*02:01, A*30:01, B*07:02, B*45:01	F	36	resolved	incidental serological finding of resolved HBV infection at check-up (anti-HBs negative, anti-HBc positive, HBsAg negative, HBV-PCR negative)				not applicable	13
R-23	A*02:01, B*18:01, B*51:07	M	64	resolved	incidental serological finding of resolved HBV infection at check-up (anti-HBs positive, anti-HBc positive)				not applicable	38

R-24	A*02:05, A*25:01, B*15:01, B*50:01	F	56	resolved	incidental serological finding of resolved HBV infection at check-up (anti-HBs negative, anti-HBc positive, HBsAg negative, HBV-PCR negative)				not applicable	41
R-25	A*03:01, A*25:01, B*07:02, B*40:02	F	37	resolved	incidental serological finding of resolved HBV infection at check-up (anti-HBs positive, anti-HBc positive)				not applicable	19
R-26	A*02:01, A*03:01, B*13:02, B*49:01	F	49	resolved	documented history of acute HBV infection with spontaneous resolution 5 months ago				not applicable	47
R-27	A*02:01, A*24:02, B*15:01, B*44:02	F	63	resolved	documented history of acute HBV infection with spontaneous resolution 5 years ago				not applicable	41
A-1	A*01:01, A*02:01, B*15:01, B*51:01	M	38	acute	presentation with jaundice during acute hepatitis B virus infection, later documented spontaneous resolution				291 496	3019
A-2	A*02:01, A*30:01, B*13:02, B*15:01	F	40	acute	presentation with jaundice during acute hepatitis B virus infection, later documented spontaneous resolution				6 111	7751
A-3	A*01:01, A*02:01, B*15:17, B*44:03	M	30	acute	presentation with jaundice during acute hepatitis B virus infection, later documented spontaneous resolution				751 288	2310
A-4	A*01:01, A*02:01, B*40:02, B*49:01	M	43	acute	presentation with jaundice during acute hepatitis B virus infection, later documented spontaneous resolution				50 153	748
A-5	A*02:01, A*33:01, B*07:02, B*14:02	F	45	acute	presentation with jaundice during acute hepatitis B virus infection, later documented spontaneous resolution				20 487	4372
ChR-1	A*02:01, A*24:02, B*15:13	F	60	chronic resolved	spontaneous clearance, before >5 years documented chronic HBV infection		no	no	not applicable	18
ChR-2	A*03:01, A*68:02, B*38:01, B*41:01	F	58	chronic resolved	spontaneous clearance, history of chronic HBV infection		no	no	not applicable	18
ChR-3	A*03:01, A*24:02, B*08:01, B*53:01	M	37	chronic resolved	spontaneous clearance, before >10 years documented chronic HBV infection		no	no	not applicable	28
ChR-4	A*01:01, A*68:02, B*38:01, B*52:01	F	31	chronic resolved	spontaneous clearance, before >5 years documented chronic HBV infection		no	no	not applicable	23

Table S2 Epitope fine-mapping

Details on experimental fine-mapping of minimal optimal epitopes within positive overlapping peptides.

Patient information		Responses to overlapping peptides (OLP)				Previously described optimal epitope				Best prediction optimal epitope (netMHCpan + ANN 4.0; 8/9/10-mers)					Viral sequence
**ID**	**HLA type**	**Number of positive OLP**	**Strength of response (%IFNγ⁺/ CD8⁺)**	**Position of OLP(s)**	**Sequence of OLP**	**Position**	**Sequence**	**HLA restriction**	**Experimentally validated?**	**Position**	**Sequence**	**HLA restriction**	**Epitope experimentally validated?**	**HLA-restriction experimentally validated?**	**Autologous patient's viral sequence**
CH-1	A*02:01, A*24:02, B*18:01, B*39:01	178/179	2.23	Precore 29-Core24	GMDIDPY**KEFGATVELLS**FLPSDFF	none				Core 7-15	KEFGATVEL	B*18:01	yes	yes	KEFGA**S**VEL
CH-2	A*02:01, A*03:01, B*35:03, B*51:01	62	1.22	Polymerase 428-445	LHPAAMPHLLVGSSGLSR	none				Pol 429-436	HPAAMPHL	B*35:03	yes	yes	HPAAMPHL(L)
CH-2	A*02:01, A*03:01, B*35:03, B*51:01	175	2.33	Precore 8-25	LIISCSCPTVQASKLCLG	none				Precore 14-22	CPTVQASKL	B*35:03	yes	yes	CPTVQASKL
CH-3	A*03:01, A*24:02, B*14:02, B*35:02	19	0.58	Polymerase 127-144	KGIKPYYPEHLVNHYFQT	none				Pol 132-142	YYPEHLVNHYF	A*24:02	yes	yes	(Y)YPEH**V**VNHYF
CH-3	A*03:01, A*24:02, B*14:02, B*35:02	77	43.57	Polymerase 533-550	CLAFSYMDDVVLGAKSVQ	none				Pol 536-544	FSYMDDVVL	B*35:02	yes	no	FSYMDDVVL

CH-3	A*03:01, A*24:02, B*14:02, B*35:02	102	0.30	Polymerase 708-725	FLAPLPIHTAELLAACFA	none				Pol 712-719	LPIHTAEL	B*35:02	yes	yes	LPIHTAEL
CH-3	A*03:01, A*24:02, B*14:02, B*35:02	204	0.18	X 1-18	MAARLCCQLDPARDVLCL	none				X10-18	DPARDVLCL	B*35:02	yes	yes	D**T**ARDVLCL
CH-4	A*02:01, A*24:02, B*08:01, B*1801	none													
CH-5	A*30:02, A*68:01, B*18:01, B*40:01	none													
CH-6	A*02:01, A*24:02, B*15:01, B*1801	93/94	1.18	Polymerase 645-669	PLYACIQ**SKQAFTFSPTY**KAFLCKQ	Pol 654-663	QAFTFSPTYK	A*02:01	nd						QAFTFSPTYK
CH-7	A*01:01, A*30:01, B*35:08, B*51:01	none													
CH-8	A*11:01, A*24:02, B*18:01, B*37:01	none													
CH-9	A*02:01, A*03:01,	none													

	B*18:01, B*4901														
CH-10	A*02:01, A*32:01, B*08:01, B*35:03	none													
CH-11	A*01:01, A*24:02, B*35:03, B*44:02	none													
CH-12	A*24:02, A*32:01, B*18:01, B*40:02	none													
CH-13	A*24:02, A*33:01, B*14:02, B*49:01	209	0.92	X 36-53	TLSSPSPSAVSTDHGAHL	none				X 45-53	VSTDHGAHL	B*14:02	yes	yes	VS**SGL**GAHL
CH-14	A*03:01, A*24:02, B*15:01, B*35:03	17	0.78	Polymerase 113-130	ARFYPNVTKYLPLDKGIK	none				Pol 114-123	RFYPNVTKYL	A*24:02	yes	nd	RFYPNVTKYL
CH-14	A*03:01, A*24:02, B*15:01, B*35:03	180	2.44	Core 14-31	ELLSFLPSDFFPSVRDLL	Core 19-27	LPSDFFPSV	B*35:01	yes						LP**A**DFFPS (V)
CH-15	A*11:01, A*24:02, B*27:02, B*44:02	none													

CH-16	A*02:01, A*03:01, B*44:03, B*44:05	none													
CH-17	A*02:01, A*23:01, B*07:05, B*44:03	16/17	0.32	Polymerase 106-130	RLQLIMP**ARFYPNVTKYL**PLDKGIK	none				Pol 115-123	FYPNVTKYL	A*23:01	nd	nd	FYPNVTKYL
CH-17	A*02:01, A*23:01, B*07:05, B*44:03	22	0.24	Polymerase 148-165	LHTLWKAGILYKRETTHS	Pol 149-159	HTLWKAGILYK	A*02:01	nd						HTLWKAGILYK
CH-17	A*02:01, A*23:01, B*07:05, B*44:03	24	0.31	Polymerase 162-179	TTHSASFCGSPYSWEQEL	none				Pol 171-179	SPYSWEQEL	B*07:05	yes	yes	SPYSWEQEL
CH-17	A*02:01, A*23:01, B*07:05, B*44:03	27	0.40	Polymerase 183-200	AESFHQQSSGILSRPPVG	none				Pol 192-199 / Pol 183-191	GILSRPPV / AESFHQQSS	A*02:01 / B*44:03	nd	nd	GILSRPPV / AESFHQQSS
CH-17	A*02:01, A*23:01, B*07:05, B*44:03	45	0.92	Polymerase 309-326	VFPCWWLQFRNSKPCSDY	none				Pol 309-317	VFPCWWLQF	A*23:01	yes	nd	VFPCWWLQF
CH-17	A*02:01, A*23:01, B*07:05, B*44:03	62	0.33	Polymerase 428-445	LHPAAMPHLLVGSSGLSR	none				Pol 429-437	HPAAMPHLL	B*07 / B*07:05	nd	nd	HPAAMPHLL
CH-17	A*02:01, A*23:01,	67	0.70	Polymerase 463-480	TMQNLHDSCSRNLYVSLL	none				Pol 468-476	HDSCSRNLY	B*44:03	nd	nd	HDSCSR**H**LY

	B*07:05, B*44:03														
CH-17	A*02:01, A*23:01, B*07:05, B*44:03	72	1.07	Polymerase 498-515	ILGFRKIPMGVGLSPFLL	Pol 504-512	IPMGVGLSP	B*07:02	yes						IPMGVGLSPFL
CH-17	A*02:01, A*23:01, B*07:05, B*44:03	107	1.55	Polymerase 743-760	SRKYTSFPWLLGCAANWI	Pol 752-760	LLGCAANWI	A*02:01	yes						LLGCAANWI
CH-17	A*02:01, A*23:01, B*07:05, B*44:03	224	0.34	X 141-154	LVCAPAPCNFFTSA	X 142-150	VCAPAPCNF	A*02	nd						VCAPAPCNF
CH-18	A*33:01, B*14:02	none													
CH-19	A*03:01, A*68:02, B*27:02, B*38:01	none													
CH-20	A*26:01, A*31:01, B*07:02, B*51:01	none													
CH-21	A*24:02, A*66:01, B*41:02, B*51:01	none													
CH-22	A*03:01, A*11:01,	185	2.03	Core 49-66	SPHHTALRQAILCWGELM	none				Core 57-66	QAILCWGELM	B*35:01	yes	yes	QAILCWGELM

	B*35:01, B*37:01														
CH-23	A*01:01, A*02:05, B*44:02, B*51:01	16	0.33	Polymerase 106-123	RLQLIMPARFYPNVTKYL	none				Pol 111-119	MPARFYPNV	B*51:01	yes	yes	MPARFYPNV
CH-23	A*01:01, A*02:05, B*44:02, B*51:01	79	0.62	Polymerase 547-564	KSVQHLESLFTAVTNFLL	none				Pol 556-564	FTAVTNFLL	A*02:05	yes	yes	FTAVTNFLL
CH-24	A*01:01, A*11:01, B*18:01, B*35:02	204	0.91	X 1-18	MAARLCCQLDPARDVLCL	none				X10-18	DPARDVLCL	B*35:02	yes	yes	D**T**ARDVLCL
CH-25	A*01:01, A*32:01, B*08:01	none													
CH-26	A*01:01, A*03:01, B*37:01, B*51:01	none													
CH-27	A*01:01, A*03:01, B*13:02, B*35:01	72	0.74	Polymerase 498-515	ILGFRKIPMGVGLSPFLL	none				Pol 504-513	IPMGVGLSPF	B*35:01	yes	nd	IPMGVGLSPF
CH-27	A*01:01, A*03:01, B*13:02, B*35:01	75	0.21	Polymerase 519-536	TSAICSVVRRAFPHCLAF	Pol 520-228	SAICSVVRR	A*03:01	tested negative						SAICSVVRR

CH-28	A*26:01, A*30:01, B*13:02, B*35:01	186	0.96	Core 56-73	RQAILCWGELMTLATWVG	none				Core 57-66	QAILCWGELM	B*35:01	yes	yes	nd
CH-28	A*26:01, A*30:01, B*13:02, B*35:01	193	0.81	Core 105-122	ISCLTFGRETVIEYLVSF	none				Core 113-122 / Core 114-122	ETVIEYLVSF / TVIEYLVSF	A*26:01 / B*35:01	yes / yes	yes / yes	ETVVEYLVSF / TVVEYLVSF
CH-29	A*02:01, B*35:01, B*40:01	none													
CH-30	A*02:01, A*25:01, B*14:02, B*18:01	none													
CH-31	A*03:01, A*2402, B*35:01	24	0.57	Polymerase 162-179	TTHSASFCGSPYSWEQEL	Pol 171-179	SPYSWEQEL	B*35	yes						SPYSWEQEL
CH-31	A*03:01, A*2402, B*35:01	72	0.31	Polymerase 498-515	ILGFRKIPMGVGLSPFLL	none				Pol 504-513	IPMGVGLSPF	B*35:01	yes	yes	IPMGVGLSPF
CH-32	A*03:01, A*25:01, B*07:02, B*57:01	none													
CH-33	A*01:01, A*66:01, B*41:02, B*57:01	none													

CH-34	A*03:01, A*31:01, B*35:01, B*57:01	186	0.40	Core 56-73	RQAILCWGELMTLATWVG	none				Core 57-66	QAILCWGELM	B*35:01	yes*	yes	QAILCWGELM
CH-34	A*03:01, A*31:01, B*35:01, B*57:01	194	0.22	Core 112-129	RETVIEYLVSFGVWIRTP	none				Core 114-122	TVIEYLVSF	B*35:01	yes	nd	IVIEYLVSF
CH-35	A*25:01, A*32:01, B*18:01, B*57:01	none													
CH-36	A*01:01, A*02:01, B*40:06, B*41:01	none													
CH-37	A*02:01, A*31:01, B*15:01, B*40:01	216	0.64	X 85-102	AHQFLPKVLHKRTLGLSV	X 92-100	(K)VLHKRTLGL	A*02:01	nd						(K)VLHKRTLGL
CH-37	A*02:01, A*31:01, B*15:01, B*40:01	219	0.36	X 106-123	TDLEAYFKDCLFKDWEEL	X 115-123	CLFKDWEEL	A*02.01	nd						CLFKDWEEL
CH-38	A*02:01, A*74:03, B*18:01, B*44:03	none													
CH-39	A*02:01, A*24:02,	none													

	B*07:02, B*44:03														
CH-40	A*01:01, A*02:01, B*07:02, B*18:01	51	0.97	Polymerase 351-368	IPRTPARVTGGVFLVDKN	Pol 351-363	IPRTPARVTGGVF	B*07:02	yes						IPRTPARVTGGVF
CH-40	A*01:01, A*02:01, B*07:02, B*18:01	72	1.07	Polymerase 498-515	ILGFRKIPMGVGLSPFLL	Pol 504-515	IPMGVGLSPFL	B*07:02	yes						IPMGVGLSPFL
CH-40	A*01:01, A*02:01, B*07:02, B*18:01	102	3.78	Polymerase 708-725	FLAPLPIHTAELLAACFA	Pol 710-719	APLPIHTAEL	B*07:02	yes						APLPIHTAEL
CH-40	A*01:01, A*02:01, B*07:02, B*18:01	206	0.28	X 15-32	VLCLRPVGAESRGRPFSG	X 15-23	VLCLRPVGA	A*02:01	nd						VLCLRPVGA
CH-41	A*24:02, A*32:01, B*35:03, B*35:08	180	0.60	Core 14-31	ELLSFLPSDFFPSVRDLL	Core 19-27	LPSDFFPSV	B*35:03	yes						LPSDFFPSV
CH-42	A*11:01, A*24:02, B*07:02, B*35:01	51	0.16	Polymerase 351-368	IPRTPARVTGGVFLVDKN	Pol 351-363	IPRTPARVTGGVF	A*11:01 / **B*07:02** / B*35:01	yes						nd
CH-42	A*11:01, A*24:02, B*07:02, B*35:01	186	1.75	Core 56-73	RQAILCWGELMTLATWVG	none				Core 57-66	QAILCWGELM	B*35:01	yes*	yes	nd

CH-42	A*11:01, A*24:02, B*07:02, B*35:01	193	1.77	Core 105-122	ISCLTFGRETVIEYLVSF	none				Core 114-122	TVIEYLVSF	B*35:01	yes	yes	TV**<u>L</u>**EYLVSF
CH-43	A*24:02, B*35:02	216	1.61	X 85-102	AHQFLPKVLHKRTLGLSV	none				X 89-98	LPKVLHKRTL	B*35:02	yes*	nd	LPKVLHKRTL
CH-44	A*01:01, A*26:01, B*08:01, B*38:01	none													
CH-45	A*03:01, A*03:02, B*44:02, B*51:01	none													
CH-46	A*03:01, A*32:01, B*13:02, B*40:02	none													
CH-47	A*02:01, A*33:01, B*14:02, B*18:01	70/71	0.43	Polymerase 484-508	QTFGRKL**<u>HLYSHPIILGF</u>**RKIPMGV	Pol 491-499 / Pol 489-497	HLYSHPIIL / KLHLYSHPI	A*02:01	yes						HLYSHPIIL / KLHLYSHPI
CH-47	A*02:01, A*33:01, B*14:02, B*18:01	92	0.34	Polymerase 638-655	CGYPALMPLYACIQSKQA	Pol 642-650	ALMPLYACI	A*02:01	tested negative						ALMPLYACI
CH-47	A*02:01, A*33:01, B*14:02, B*18:01	102/103	5.53	Polymerase 708-732	FLAPLPI**<u>HTAELLAACFA</u>**RSRSGAN	none				Pol 718-724	ELLAACF	B*18:01	yes	yes	ELLAACF

CH-48	A*03:02, A*11:01, B*35:01, B*41:02	none													
CH-49	A*01:01, A*02:01, B*35:03, B*51:01	16	0.68	Polymerase 106-123	RLQLIMPARFYPNVTKYL	none				Pol 111-119	MPARFYPNV	B*51:01	yes	yes	MPARFYPN**F**
CH-49	A*01:01, A*02:01, B*35:03, B*51:01	106/107	7.90	Polymerase 736-760	TDNSVVL**SRKYTSFPWLL**GCAANWI	Pol 752-760	LLGCAANWI	A*02:01	nd						LLGCAANWI
CH-50	A*30:01, B*41:01	75	0.76	Polymerase 519-536	TSAICSVVRRAFPHCLAF	none				Pol 525-533	VVRRAFPHC	A*30:01	yes	nd	VVRRAFPHC
CH-51	A*26:01, B*27:05, B*51:01	45	2.84	Polymerase 309-326	VFPCWWLQFRNSKPCSDY	none				Pol 317-326	FRNSKPCSDY	B*27:05	yes	yes	nd
CH-52	A*03:01, A*68:01, B*27:02, B*57:01	none													
CH-53	A*02:01, B*35:01, B*52:01	none													
CH-54	A*02:01, B*18:01, B*44:02	none													
CH-55	A*02:05, A*03:01,	none													

	B*44:03, B*50:01														
CH-56	A*03:01, A*26:01, B*07:02, B*41:01	70/71	0.23/0.16	Polymerase 484 - 508	QTFGRKL**HLYSHPIILGF**RKIPMGV	none				Pol 493-501	YSHPIILGF	A*26:01	nd	nd	nd
CH-56	A*03:01, A*26:01, B*07:02, B*41:01	171	0.16	Envelope 358 - 375	IWMMWYWGPSLYSILSPF	none				Env 360-369 / Env 367-375	MMWYWGPSLY / SLYSILSPF	A*03:01 / A*26:01	nd	nd	nd
CH-56	A*03:01, A*26:01, B*07:02, B*41:01	196	0.50	Core 126 - 143	IRTPPAYRPPNAPILSTL	none				Core 133-142	RPPNAPILST	B*07:02	nd	nd	nd
R-1	A*01:01, A*02:01, B*14:01, B*38:01	none													
R-2	A*02+	165/166	15.70/12.80	Envelope 316-333 / Envelope 323-340	PSSWAF**GKFLWEWASAR**FSWLSLLV	Env 324-333	FLWEWASA	A*02:01	nd						
R-3	A*01:01, A*11:01, B*40:06, B*44:03	none													
R-4	A*01:01, A*02:01, B*35:01, B*51:01	166	0.31	Envelope 323-340	KFLWEWASARFSWLSLLV	Env 324-333	FLWEWASA	A*02:01	nd						

R-4	A*01:01, A*02:01, B*35:01, B*51:01	167	0.29	Envelope 330-347	SARFSWLSLLVPFVQWFV	Env 335-343 / Env 338-347	WLSLLVPFV / LLVPFVQWFV	A*02:01 / A*02:01	nd						
R-4	A*01:01, A*02:01, B*35:01, B*51:01	172	0.30	Envelope 365-382	GPSLYSILSPFLPLLPIF	Env 370/371-379	(S)ILSPFLPLL	A*02:01	nd						
R-4	A*01:01, A*02:01, B*35:01, B*51:01	207	0.35	X 22-39	GAESRGRPFSGSLGTLSS	none				X 28-37/38	RPFSGSLGT(L)	B*35:01	yes*	nd	
R-4	A*01:01, A*02:01, B*35:01, B*51:01	212	0.57	X 57-74	GLPVCAFSSAGPCALRFT	none				X 58-66 / X65-73	LPVCAFSSA / SAGPCALRF	B*35:01	yes* / nd	nd	
R-5	A*03:01, A*68:01, B*35:02, B*39:01	none													
R-6	A*03:01, A*26:01, B*15:17, B*38:01	none													
R-7	A*23:01, A*68:01, B*44:03, B*51:01	none													

R-8	A*02:01, B*35:02, B*57:01	24	1.52	Polymerase 162-179	TTHSASFCGSPYSWEQEL	Pol 171-179	SPYSWEQEL	B*35:01	nd						
R-8	A*02:01, B*35:02, B*57:01	67	0.88	Polymerase 463-480	TMQNLHDSCSRNLYVSLL	none				Pol 471-479	CSRNLYVSL / HDSCSRNLY	B*57:01 / B*35:01	nd / nd	nd	
R-9	A*01:01, A*11:01, B*37:02, B*52:01	198	0.33	Core 140-159	LSTLPETTVVRRRGRSPR	Core 141-150	STLPETTVVR	A*11:01	yes						
R-10	A*03:01, A*23:01, B*07:02, B*44:03	none													
R-11	A*02:01, A*11:01, B*15:02, B*18:01	none													
R-12	A*03:01, A*24:02, B*27:05, B*52:01	none													
R-13	A*01:01, A*24:02, B*35:02, B*38:01	none													
R-14	A*03:01, A*68:01,	51	0.51	Polymerase 351-368	IPRTPARVTGGVFLVDKN	Pol 351-363	IPRTPARVTGGVF	B*07:02	yes						

	B*07:02, B*35:03														
R-14	A*03:01, A*68:01, B*07:02, B*35:03	105	0.37	Polymerase 729-746	SGANILGTDNSVVLSRKY	none				Pol 735-744 / Pol 737-745	GTDNSVVLSR / DNSVVLSRK	A*68:01	nd	nd	
R-15	A*01:01, A*24:02, B*07:02, B*37:01	23	0.08	Polymerase 155-172	GILYKRETTHSASFCGSP	none				Pol 160-168 / Pol 158-166	RETTHSASF / YKRETTHSA	B*37:01 / B*07:02	nd / nd	nd	
R-15	A*01:01, A*24:02, B*07:02, B*37:01	66	0.10	Polymerase 456-473	IFNHQHGTMQNLHDSCSR	none				Pol 456-464 / Pol 459-477	IFNHQHGTM / HQHGTMQNL	A*24:02 / B*37:01	yes* / nd	nd	
R-15	A*01:01, A*24:02, B*07:02, B*37:01	166	0.04	Envelope 323-340	KFLWEWASARFSWLSLLV	Env 325-333	LWEWASARF	A*24:02	yes						
R-16	A*03:01, A*24:02, B*07:02, B*35:01	none													
R-17	A*02:01, B*13:02, B*44:03	23	1.44	Polymerase 155-172	GILYKRETTHSASFCGSP	none				Pol 160-168	RETTHSASF	B*44:03	nd	nd	
R-17	A*02:01, B*13:02, B*44:03	111	0.35	Polymerase 771-788	SALNPADDPSRGRLGLSR	none				Pol 772-780	ALNPADDPS / ADDPSRGRL	A*02:01 / B*44:03	nd	nd	

R-17	A*02:01, B*13:02, B*44:03	193	2.17	Core 105-122	ISCLTFGRETVIEYLVSF	Core 107-115	CLTFGRETV	A*02:01	nd						
R-18	A*24:02, A*24:10, B*15:02, B*48:03	none													
R-19	A*11:01, A*24:02, B*13:02, B*52:01	44	0.53	Polymerase 302-319	RSQSERPVFPCWWLQFRN	none				Pol 309-317	VFPCWWLQF	A*24:02	yes	nd	
R-20	A*01:01, A*02:01, B*08:01, B*18:01	23	0.70	Polymerase 155-172	GILYKRETTHSASFCGSP	none				Pol 160-168	RETTHSASF	B*18:01	nd	nd	
R-20	A*01:01, A*02:01, B*08:01, B*18:01	172	0.41	Envelope 365-382	GPSLYSILSPFLPLLPIF	Env 370/371-379	(S)ILSPFLPLL	A*02:01	nd						
R-21	A*68:01, B*15:01, B*35:03	94	1.82	Polymerase 652-669	SKQAFTFSPTYKAFLCKQ	none				Pol 653-662	KQAFTFSPTY	B*15:01	nd	nd	
R-21	A*68:01, B*15:01, B*35:03	157	0.41	Envelope 260-277	LLDYQGMLPVCPLIPGSS	none				Env 263-272	YQGMLPVCPL	B*15:01	nd	nd	
R-21	A*68:01, B*15:01, B*35:03	198	6.05	Core 140-157	LSTLPETTVVRRRGRSPR	Core 141-151	STLPETTVVRR	A*68:01	nd						
R-22	A*02:01, A*30:01,	179	1.32	Core 7-24	KEFGATVELLSFLPSDFF	Core 18-27	FLPSDFFPSV	A*02:01	yes						

	B*07:02, B*45:01														
R-23	A*02:01, B*18:01, B*51:07	none													
R-24	A*02:01, A*03:01, B*07:02, B*15:01	none													
R-25	A*02:05, A*25:01, B*15:01, B*50:01	none													
R-26	A*02:01, A*24:02, B*15:01, B*44:02	100	0.25	Polymerase 694 - 710	GWGLVMGHQRMRGTFLAP	none				Pol 701 - 709	HQRMRGTFL	B*15:01	nd	nd	
R-26	A*02:01, A*24:02, B*15:01, B*44:02	170	0.12	Envelope 188 - 205	PTVWLSVIWMMWYWGPSL	Env 360 - 368	MMWYWGPSL	A*02:01/A*24:02	nd						
R-26	A*02:01, A*24:02, B*15:01, B*44:02	190	0.22	Core 84 - 101	LVVSYVNTNMGLKFRQLL	Core 87 - 95	SYVNTNMGL	A*24:02	nd						
R-27	A*02:01, A*03:01, B*13:02, B*49:01	63	0.19	Polymerase 435 - 452	HLLVGSSGLSRYVARLSS	Pol 455-463	GLSRYVARL	A*02:01	yes						

R-27	A*02:01, A*03:01, B*13:02, B*49:01	180	0.18	Core 14 - 31	ELLSFLPSDFFPSVRDLL	Core 18-27	FLPSDFFPSV	A*02:01	yes						
R-27	A*02:01, A*03:01, B*13:02, B*49:01	198	0.88	Core 140 - 157	LSTLPETTVVRRRGRSPR	Core 141 - 150/1	STLPETTVV(RR)	A*02:01	nd						
A-1	A*01:01, A*02:01, B*15:01, B*51:01	63	2.85	Polymerase 435 - 452	HLLVGSSGLSRYVARLSS	Pol 455-463	GLSRYVARL(S)	A*02:01	tested negative						
A-1	A*01:01, A*02:01, B*15:01, B*51:01	70	0.37	Polymerase 484 - 501	QTFGRKLHLYSHPIILGF	Pol 489-497	KLHLYSHPI	A*02:01	nd						
A-1	A*01:01, A*02:01, B*15:01, B*51:01	143	0.97	Envelope 162-179	LNMENITSGFLGPLLVLQ	none				Env 162 - 171 / Env 171 - 178	LNMENITSGF / FLGPLLVL	B*15:01 / A*02:01	nd	nd	
A-2	A*02:01, A*30:01, B*13:02, B*15:01	167/168	3.97/1.08	Envelope 330-347/ Envelope 337-354	SARFSWL**SLLVPFVQWFV**GLSPTVW	Env 335-343	WLSLLVPFV	A*02:01	yes						
A-2	A*02:01, A*30:01, B*13:02, B*15:01	187	0.19	Core 63-80	GELMTLATWVGGNLEDPI	none				Core 63-72	GELMTLATWV	A*02:01	yes	nd	
A-3	A*01:01, A*02:01,	143	0.61	Envelope 162-179	LNMENITSGFLGPLLVLQ	none				Env 167-175	ITSGFLGPL	B*15:17	yes	nd	

	B*15:17, B*44:03														
A-4	A*01:01, A*32:01, B*40:02, B*49:01	13/14	0.17/ 0.27	Polymerase 87-104/Polymerase 94-111	HQDIIKK**CEQFVGPLTVN**EKRRLQL	none				Pol 92-101	(C)EQFVGPLTV	B*49:01 / B*40:02	nd	nd	
A-4	A*01:01, A*32:01, B*40:02, B*49:01	43/44	4.56/ 1.71	Polymerase 295-312/ Polymerase 302-319	NLPPNSA**RSQSERPVFP**CWWLQFRN	none				Pol 305-312 / Pol 302-310	SERPVFPC / RSQSERPVF	B*40:02 / A*32:01	nd	nd	
A-4	A*01:01, A*32:01, B*40:02, B*49:01	59	0.62	Polymerase 407-422	LLSSNLSWLSLDVSAAFY	none				Pol 416-424	SLDVSAAFY	A*01:01	yes*	nd	
A-4	A*01:01, A*32:01, B*40:02, B*49:01	75	5.73	Polymerase 519-536	TSAICSVVRRAFPHCLAF	none				Pol 528-536	RAFPHCLAF	A32:01	nd	nd	
A-4	A*01:01, A*32:01, B*40:02, B*49:01	178/179	3.62/14.62	Precore 29-Core 17/ Core 7-24	GMDIDPY**KEFGATVELLS**FLPSDFF	none				Core 7-15	KEFGATVEL	B*40:02	nd	nd	
A-4	A*01:01, A*32:01, B*40:02, B*49:01	186	6.15	Core 56-73	RQAILCWGELMTLATWVG	none				Core 63-73	GELMTLATWV	B*40:02 / B*49:01	nd	nd	
A-4	A*01:01, A*32:01,	194	0.59	Core 112-129	RETVIEYLVSFGVWIRTP	Core 112-122	RETVIEYLVSF	B*49:01	nd						

	B*40:02, B*49:01														
A-5	A*02:01, A*33:01, B*07:02, B*14:02	51	1.44	Polymerase 351-368	IPRTPARVTGGVFLVDKN	Pol 357-362 / Pol 354-362/363	RVTGGVFLV / TPARVTGGV(F)	A*02:01 / B*07:02	nd						
A-5	A*02:01, A*33:01, B*07:02, B*14:02	146	1.38	Envelope 183-200	FLLTRILTIPQSLDSWWT	Env 183-191	FLLTRILTI	A*02:01	nd						
A-5	A*02:01, A*33:01, B*07:02, B*14:02	148	1.04	Envelope 197-214	SWWTSLNFLGGTTVCLGQ	Env 199-207	WTSLNFLGG	A*02:01	nd						
A-5	A*02:01, A*33:01, B*07:02, B*14:02	167	6.98	Envelope 330-347	SARFSWLSLLVPFVQWFV	Env 335-343 / Env 338-347	WLSLLVPFV / LLVPFVQWFV	A*02:01	nd						
A-5	A*02:01, A*33:01, B*07:02, B*14:02	180	4.87	Core 14-31	ELLSFLPSDFFPSVRDLL	Core 18-27 / Core 19-27	FLPSDFFPSV / LPSDFFPSV	A*02:01, B*07:02	nd						
A-5	A*02:01, A*33:01, B*07:02, B*14:02	192	0.27	Core 98-115	RQLLWFHISCLTFGRETV	Core 100-108 / Core 104-112 / Core 107-115	LLWFHISCL / HISCLTFGR / CLTFGRETV	A*02:01, A*33:01, A*02:01	nd						

A-5	A*02:01, A*33:01, B*07:02, B*14:02	196/197	11.55/ 4.69	Core 126-143/ Core 133-150	IRTPPAY**RPPNAPIL** **STL**PETTVVR	Core 133-140 / Core 139-148	RPPNAPIL / (IL)STLPET TV	B*07:02 / A*02:01	nd						
ChR -1	A*02:01, A*24:02, B*15:13	none													
ChR -2	A*03:01, A*68:02, B*38:01, B*41:01	219	0.72	X 106-123	TDLEAYFKDCLFKD WEEL	none				X109-118 / X108-116	EAYFKDCL FK / LEAYFKDC L	A*03:01 / B*41:01	nd	nd	
ChR -3	A*03:01, A*24:02, B*08:01, B*53:01	none													
ChR -4	A*01:01, A*68:02, B*38:01, B*52:01	79 / 80	1.9 / 0.56	Pol 560-584	KSVQHLE**SLFTAVT** **NFLL**SLGIHLN	none				Pol 556-564	FTAVTNFL L	A*68:02	nd	nd	

Table S3. Epitope list

List of HBV-specific CD8+ T cell epitopes targeted in this study.

**Position Genotype D**	**sequence**	**HLA Restriction**	**previously described vs new**	**minimal epitope experimentally tested/ validated**	**HLA-restriction experimentally validated**
Precore 14-22	CPTVQASKL	B*35:03	new	yes	yes
Core 7-15	KEFGATVEL	B*18:01	new	yes	yes
Core 7-15	KEFGATVEL	B*40:02	new	no	no
Core 18-27	FLPSDFFPSV	A*02:01	previously described	yes	no
Core 19-27	LPSDFFPSV	B*35:01	previously described	yes	yes
Core 19-27	LPSDFFPSV	B*35:03	previously described	yes	yes
Core 19-27	LPSDFFPSV	B*07:02	previously described	no	no
Core 57-66	QAILCWGELM	B*35:01	new	yes	yes
Core 63-72	GELMTLATWV	A*02:01	new	yes	no
Core 63-73	GELMTLATWV	B*40:02 / B*49:01	new	no	no
Core 87 - 95	SYVNTNMGL	A*24:02	previously described	no	no
Core 107-115	CLTFGRETV	A*02:01	previously described	no	no
Core 100-108	LLWFHISCL	A*02:01	previously described	no	no
Core 104-112	HISCLTFGR	A*33:01	previously described	no	no
Core 112-122	RETVIEYLVSF	B*49:01	previously described	no	no
Core 113-122	ETVIEYLVSF	A*26:01	new	yes	yes
Core 114-122	TVIEYLVSF	B*35:01	new	yes	yes
Core 133-140	RPPNAPIL	B*07:02	previously described	no	no
Core 133-142	RPPNAPILST	B*07:02	new	no	no
Core 139-148	(IL)STLPETTV	A*02:01	previously described	no	no
Core 141 - 150/1	STLPETTVV(RR)	A*02:01	previously described	no	no
Core 141-150	STLPETTVVR	A*11:01	previously described	yes	no
Core 141-151	STLPETTVVRR	A*68:01	previously described	no	no
Env 162 - 171	LNMENITSGF	B*15:01	new	no	no
Env 167-175	ITSGFLGPL	B*15:17	new	yes	no

Env 171 - 178	FLGPLLVL	A*02:01	new	no	no
Env 183-191	FLLTRILTI	A*02:01	previously described	no	no
Env 199-207	WTSLNFLGG	A*02:01	previously described	no	no
Env 263-272	YQGMLPVCPL	B*15:01	new	no	no
Env 324-333	FLWEWASA	A*02:01	previously described	no	no
Env 325-333	LWEWASARF	A*24:02	previously described	yes	no
Env 335-343	WLSLLVPFV	A*02:01	previously described	yes	no
Env 338-347	LLVPFVQWFV	A*02:01	previously described	no	no
Env 360 - 368	MMWYWGPSL	A*02:01/A*24:02	previously described	no	no
Env 360-369	MMWYWGPSLY	A*03:01	new	no	no
Env 367-375	SLYSILSPF	A*26:01	new	no	no
Env 370/371-379	(S)ILSPFLPLL	A*02:01	previously described	no	no
Pol 17-25	EAGPLEEEL	B*35	new	no	no
Pol 92-101	(C)EQFVGPLTV	B*49:01 / B*40:02	new	no	no
Pol 111-119	MPARFYPNV	B*51:01	new	yes	yes
Pol 114-123	RFYPNVTKYL	A*24:02	new	yes	no
Pol 115-123	FYPNVTKYL	A*23:01	new	no	no
Pol 132-142	YYPEHLVNHYF	A*24:02	new	yes	yes
Pol 149-159	HTLWKAGILYK	A*02:01	previously described	no	no
Pol 158-166	YKRETTHSA	B*07:02	new	no	no
Pol 160-168	RETTHSASF	B*37:01	new	no	no
Pol 160-168	RETTHSASF	B*44:03	new	no	no
Pol 160-168	RETTHSASF	B*18:01	new	no	no
Pol 171-179	SPYSWEQEL	B*35:01	previously described	yes	yes
Pol 171-179	SPYSWEQEL	B*07:05	new	yes	yes
Pol 183-191	AESFHQQSS	B*44:03	new	no	no
Pol 192-199	GILSRPPV	A*02:01	new	no	no
Pol 302-310	RSQSERPVF	A*32:01	new	no	no
Pol 305-312	SERPVFPC	B*40:02	new	no	no
Pol 309-317	VFPCWWLQF	A*23:01	new	yes	no
Pol 309-317	VFPCWWLQF	A*24:02	new	yes	no
Pol 317-326	FRNSKPCSDY	B*27:05	new	yes	yes
Pol 351-363	IPRTPARVTGGVF	B*07:02	previously described	yes	yes

Pol 354-362/363	TPARVTGGV(F)	B*07:02	previously described	no	no
Pol 357-362	RVTGGVFLV	A*02:01	previously described	no	no
Pol 416-424	SLDVSAAFY	A*01:01	new	yes*	no
Pol 429-436	HPAAMPHL	B*35:03	new	yes	yes
Pol 429-437	HPAAMPHLL	B*07	new	no	no
Pol 455-463	GLSRYVARL	A*02:01	previously described	yes	no
Pol 456-464	IFNHQHGTM	A*24:02	new	yes*	no
Pol 459-477	HQHGTMQNL	B*37:01	new	no	no
Pol 468-476	HDSCSRNLY	B*35:01	new	no	no
Pol 468-476	HDSCSRNLY	B*44:03	new	no	no
Pol 471-479	CSRNLYVSL	B*57:01	new	no	no
Pol 489-497	KLHLYSHPI	A*02:01	previously described	no	no
Pol 491-499	HLYSHPIIL	A*02:01	previously described	yes	yes
Pol 493-501	YSHPIILGF	A*26:01	new	no	no
Pol 504-512	IPMGVGLSP	B*07:02	previously described	yes	yes
Pol 504-513	IPMGVGLSPF	B*35:01	new	yes	yes
Pol 504-515	IPMGVGLSPFL	B*07:02	previously described	yes	no
Pol 525-533	VVRRAFPHC	A*30:01	new	yes	no
Pol 528-536	RAFPHCLAF	A32:01	new	no	no
Pol 536-544	FSYMDDVVL	B*35:02	new	yes	no
Pol 556-564	FTAVTNFLL	A*02:05	new	yes	yes
Pol 701 - 709	HQRMRGTFL	B*15:01	new	no	no
Pol 710-719	APLPIHTAEL	B*07:02	previously described	yes	yes
Pol 712-719	LPIHTAEL	B*35:02	new	yes	yes
Pol 718-724	ELLAACF	B*18:01	new	yes	yes
Pol 735-744	GTDNSVVLSR	A*68:01	new	no	no
Pol 737-745	DNSVVLSRK	A*68:01	new	no	no
Pol 752-760	LLGCAANWI	A*02:01	previously described	yes	yes
Pol 772-780	ALNPADDPS	A*02:01	new	no	no
Pol 776-784	ADDPSRGRL	B*44:03	new	no	no
X10-18	DPARDVLCL	B*35:02	new	yes	yes
X 15-23	VLCLRPVGA	A*02:01	previously described	no	no
X 28-37/38	RPFSGSLGT(L)	B*35:01	new	yes*	no

X 45-53	VSTDHGAHL	B*14:02	new	yes	yes
X 58-66	LPVCAFSSA	B*35:01	new	yes*	no
X 65-73	SAGPCALRF	B*35:01	new	no	no
X 89-98	LPKVLHKRTL	B*35:02	new	yes*	no
X 92-100	(K)VLHKRTLGL	A*02:01	previously described	no	no
X 115-123	CLFKDWEEL	A*02.01	previously described	no	no

*in other patient

Table S4. Primers

List of primers used to determine autologous viral sequences of patients with responses to overlapping peptides. Position indicated for consensus sequence genotype D; pubmed GenBank accession code X02496.1

name	sequence	position
HBV_Pol_1extF	CACCTCTGCCTAATCATCTCTTGT	1828-1851
HBV_Pol_1extR	CTTGAGCAGGAGTCGTGCAGGT	523-544
HBV_Pol_1F	TGACTCTAGCTACCTGGGTG	2099-2118
HBV_Pol_1R	AACGGGCAACATACCTTGAT	455-474
HBV_Pol_2extF	GTGGCTCCAGTTCAGGAACAGTA	65-87
HBV_Pol_2extR	CTACAGCCTCCTAATACAAAGACCT	1764-1788
HBV_Pol_2F	GAACATGGAGAACATCACATCAG	153-175
HBV_Pol_2R	CTCAAGGTCGGTCGTTGACATT	1681-1702

Table S5. HLA-mismatched controls

PBMC of patients with chronic HBV infection were tested after in vitro expansion with all HLA-mismatched epitopes targeted in our study. Epitopes restricted by HLA-types belonging to the same HLA supertype family as patient's HLA type were excluded for possible cross-recognition

**Patient**	**age**	**Sex**	**HLA Type**	**HLA class I restriction**	**Protein**	**Sequence**	**IFN-γ+ of CD8+**
**NC1**	**52**	**m**	**A*26:01; A*68:01; B*08:01**	A*02:01	Core 18-27	FLPSDFFPSV	negative
				A*02:01	Core 100-108	LLWFHISCL	negative
				A*02:01	Core 107-115	CLTFGRETV	negative
				A*02:01	Envelope 183-191	FLLTRILTI	negative
				A*02:01	Envelope 204-212	FLGGTTVCL	negative
				A*02:01	Envelope 324-333	FLWEWASA	negative
				A*02:01	Envelope 338-347	LLVPFVQWFV	negative
				A*02:01	Envelope 370-379	SILSPFLPLL	negative
				A*02:01	Envelope 371-379	ILSPFLPLL	negative
				A*02:01	Polymerase 156-166	ILYKRETTHSA	negative
				A*02:01	Polymerase 357-362	RVTGGVFLV	negative
				A*02:01	Polymerase 455	GLSRYVARL	negative
				A*02:01	Polymerase 491-499	HLYSHPIIL	negative
				A*02:01	Polymerase 752-760	LLGCAANWI	negative
				A*02:01	Polymerase 772-780	ALNPADDPS	negative
				A*02:01	Core 63-73	GELMTLATWV	negative
				A*02:01	Envelope 335-343	WLSLLVPFV	negative
				A*02:01	X 15-23	VLCLRPVGA	negative
				A*02:01	X 92-100	VLHKRTLGL	negative
				A*02:01	X 115-123	CLFKDWEEL	negative
				A*02:05	Polymerase 556-564	FTAVTNFLL	negative
				A*23:01	Polymerase 115-123	FYPNVTKYL	negative
				A*23:01	Polymerase 309-317	VFPCWWLQF	negative
				A*24:02	Polymerase 132-142	YYPEHLVNHYF	negative
				A*24:02	Envelope 325-333	LWEWASARF	negative
				A*24:02	Polymerase 114-123	RFYPNVTKYL	negative

**NC1**				A*24:02	Polymerase 456-464	IFNHQHGTM	negative
				B*07:02	Polymerase 351-363	IPRTPARVTGGVF	negative
				B*07:02	Polymerase 354-362	TPARVTGGV	negative
				B*07:02	Polymerase 354-363	TPARVTGGVF	negative
				B*07:02	Polymerase 710-719	APLPIHTAEL	negative
				B*07:02	Core 133-140	RPPNAPIL	negative
				B*07:02	X 146-154	APCNFFTSA	negative
				B*07:05	Polymerase 149-157	HTLWKAGIL	negative
				B*07:05	Polymerase 429-437	HPAAMPHLL	negative
				B*07:05<br>B*07:02	Polymerase 504-514	IPMGVGLSPFL	negative
				B*13:02	Polymerase 526-534	VRRAFPHCL	negative
				B*14:02	X 45-53	VSTDHGAHL	negative
				B*15:01	Envelope 263-272	YQGMLPVCPL	negative
				B*15:01	Polymerase 653-662	KQAFTFSPTY	negative
				B*15:17	Envelope 167-175	ITSGFLGPL	negative
				B*18:01	Core 7-15	KEFGATVEL	negative
				B*18:01	Polymerase 718-724	ELLAACF	negative
				B*18:01	Polymerase 640-647	YPALMPLY	negative
				B*27:05	Polymerase 317-326	FRNSKPCSDY	negative
				B*35:01	Core 57-66	QAILCWGELM	negative
				B*35:01	Core 114-122	TVIEYLVSF	negative
				B*35:01	Polymerase 171-179	SPYSWEQEL	negative
				B*35:01	Polymerase 504-513	IPMGVGLSPF	negative
				B*35:01	X 28-37	RPFSGPLGTL	negative
				B*35:01	X 58-66	LPVCAFSSA	negative
				B*35:02	Polymerase 712-719	LPIHTAEL	negative
				B*35:02	X10-18	DPARDVLCL	negative
				B*35:02	Polymerase 536-544	FSYMDDVVL	negative
				B*35:02	X 89-98	LPKVLHKRTL	negative
				B*35:03	Precore 14-22	CPTVQASKL	negative
				B*35:03	Core 19-27	LPSDFFPSV	negative
				B*35:03	Polymerase 116-123	YPNVTKYL	negative
				B*35:03	Polymerase 429-436	HPAAMPHL	negative
				B*35:03	Polymerase 745-753	KYTSFPWLL	negative

**NC1**				B*37:01<br>B*44:03	Polymerase 160-168	RETTHSASF	negative
				B*40:01	Polymerase 305-312	SERPVFPC	negative
				B*44:02	Polymerase 183-193	AESFHQQSSGI	negative
				B*44:03	Polymerase 468-476	HDSCSRNLY	negative
				B*49:01	Core 112-122	RETVIEYLVSF	negative
				B*49:01	Polymerase 92-101	CEQFVGPLTV	negative
				B*51:01	Polymerase 111-119	MPARFYPNV	negative
				B*57:01	Polymerase 471-479	CSRNLYVSL	negative
**NC2**	**31**	**m**	**A*23:01;A*33:01;<br>B*14:02;B*44:03**	A*01:01	Polymerase 416-424	SLDVSAAFY	negative
				A*02:01	Core 18-27	FLPSDFFPSV	negative
				A*02:01	Core 100-108	LLWFHISCL	negative
				A*02:01	Core 107-115	CLTFGRETV	negative
				A*02:01	Envelope 183-191	FLLTRILTI	negative
				A*02:01	Envelope 204-212	FLGGTTVCL	negative
				A*02:01	Envelope 324-333	FLWEWASA	negative
				A*02:01	Envelope 338-347	LLVPFVQWFV	negative
				A*02:01	Envelope 370-379	SILSPFLPLL	negative
				A*02:01	Envelope 371-379	ILSPFLPLL	negative
				A*02:01	Polymerase 156-166	ILYKRETTHSA	negative
				A*02:01	Polymerase 357-362	RVTGGVFLV	negative
				A*02:01	Polymerase 455	GLSRYVARL	negative
				A*02:01	Polymerase 491-499	HLYSHPIIL	negative
				A*02:01	Polymerase 752-760	LLGCAANWI	negative
				A*02:01	Polymerase 772-780	ALNPADDPS	negative
				A*02:01	Core 63-73	GELMTLATWV	negative
				A*02:01	Envelope 335-343	WLSLLVPFV	negative
				A*02:01	X 15-23	VLCLRPVGA	negative
				A*02:01	X 92-100	VLHKRTLGL	negative
				A*02:01	X 115-123	CLFKDWEEL	negative
				A*02:05	Polymerase 556-564	FTAVTNFLL	negative
				A*03:02	X 130-140	KVFVLGGCRHK	negative
				A*11:01	Core 141-150	STLPETTVVR	negative
				B*07:02	Polymerase 351-363	IPRTPARVTGGV<br>F	negative
				B*07:02	Polymerase 354-362	TPARVTGGV	negative
				B*07:02	Polymerase 354-363	TPARVTGGVF	negative

**NC2**				B*07:02	Polymerase 710-719	APLPIHTAEL	negative
				B*07:02	Core 133-140	RPPNAPIL	negative
				B*07:02	X 146-154	APCNFFTSA	negative
				B*07:05	Polymerase 149-157	HTLWKAGIL	negative
				B*07:05	Polymerase 429-437	HPAAMPHLL	negative
				B*07:05<br>B*07:02	Polymerase 504-514	IPMGVGLSPFL	negative
				B*13:02	Polymerase 526-534	VRRAFPHCL	negative
				B*15:01	Envelope 263-272	YQGMLPVCPL	negative
				B*15:01	Polymerase 653-662	KQAFTFSPTY	negative
				B*15:17	Envelope 167-175	ITSGFLGPL	negative
				B*35:01	Core 57-66	QAILCWGELM	negative
				B*35:01	Core 114-122	TVIEYLVSF	negative
				B*35:01	Polymerase 171-179	SPYSWEQEL	negative
				B*35:01	Polymerase 504-513	IPMGVGLSPF	negative
				B*35:01	X 28-37	RPFSGPLGTL	negative
				B*35:01	X 58-66	LPVCAFSSA	negative
				B*35:02	Polymerase 712-719	LPIHTAEL	negative
				B*35:02	X10-18	DPARDVLCL	negative
				B*35:02	Polymerase 536-544	FSYMDDVVL	negative
				B*35:02	X 89-98	LPKVLHKRTL	negative
				B*35:03	Precore 14-22	CPTVQASKL	negative
				B*35:03	Core 19-27	LPSDFFPSV	negative
				B*35:03	Polymerase 116-123	YPNVTKYL	negative
				B*35:03	Polymerase 429-436	HPAAMPHL	negative
				B*35:03	Polymerase 745-753	KYTSFPWLL	negative
				B*49:01	Core 112-122	RETVIEYLVSF	negative
				B*49:01	Polymerase 92-101	CEQFVGPLTV	negative
				B*51:01	Polymerase 111-119	MPARFYPNV	negative
				B*57:01	Polymerase 471-479	CSRNLYVSL	negative
**NC3**	**47**	**m**	**A*01:01; A*02:01;**<br>**B*35:03; B*51:01**	A*03:02	X 130-140	KVFVLGGCRHK	negative
				A*11:01	Core 141-150	STLPETTVVR	negative
				A*24:02	Polymerase 132-142	YYPEHLVNHYF	negative
				A*24:02	Envelope 325-333	LWEWASARF	negative
				A*24:02	Polymerase 114-123	RFYPNVTKYL	negative
				A*24:02	Polymerase 456-464	IFNHQHGTM	negative
				B*13:02	Polymerase 526-534	VRRAFPHCL	negative

**NC3**				B*15:01	Envelope 263-272	YQGMLPVCPL	negative
				B*15:01	Polymerase 653-662	KQAFTFSPTY	negative
				B*15:17	Envelope 167-175	ITSGFLGPL	negative
				B*18:01	Core 7-15	KEFGATVEL	0,16%
				B*18:01	Polymerase 718-724	ELLAACF	negative
				B*18:01	Polymerase 640-647	YPALMPLY	negative
				B*27:05	Polymerase 317-326	FRNSKPCSDY	negative
				B*40:01	Polymerase 305-312	SERPVFPC	negative
				B*49:01	Core 112-122	RETVIEYLVSF	negative
				B*49:01	Polymerase 92-101	CEQFVGPLTV	negative
				B*57:01	Polymerase 471-479	CSRNLYVSL	negative
**NC4**	**43**	**m**	**A*02:01; A*230:1;**	A*01:01	Polymerase 416-424	SLDVSAAFY	negative
			**B*35:01; B*51:01**	A*03:02	X 130-140	KVFVLGGCRHK	negative
				A*11:01	Core 141-150	STLPETTVVR	negative
				A*30:01	Polymerase 525-533	VVRRAFPHC	negative
				A*32:01	Polymerase 528-536	RAFPHCLAF	negative
				B*13:02	Polymerase 526-534	VRRAFPHCL	negative
				B*15:01	Envelope 263-272	YQGMLPVCPL	negative
				B*15:01	Polymerase 653-662	KQAFTFSPTY	negative
				B*15:17	Envelope 167-175	ITSGFLGPL	negative
				B*18:01	Core 7-15	KEFGATVEL	negative
				B*18:01	Polymerase 718-724	ELLAACF	negative
				B*18:01	Polymerase 640-647	YPALMPLY	negative
				B*27:05	Polymerase 317-326	FRNSKPCSDY	negative
				B*40:01	Polymerase 305-312	SERPVFPC	negative
				B*49:01	Core 112-122	RETVIEYLVSF	negative
				B*49:01	Polymerase 92-101	CEQFVGPLTV	negative
				B*57:01	Polymerase 471-479	CSRNLYVSL	negative
**NC5**	**37**	**f**	**A*02:01; A*11:01;**	A*01:01	Polymerase 416-424	SLDVSAAFY	negative
			**B*18:01; B*35:01**	A*23:01	Polymerase 115-123	FYPNVTKYL	negative
				A*23:01	Polymerase 309-317	VFPCWWLQF	negative
				A*24:02	Polymerase 132-142	YYPEHLVNHYF	Negative
				A*24:02	Envelope 325-333	LWEWASARF	Negative
				A*24:02	Polymerase 114-123	RFYPNVTKYL	Negative
				A*24:02	Polymerase 456-464	IFNHQHGTM	Negative
				A*26:01	Core 113-122	ETVIEYLVSF	Negative
				B*13:02	Polymerase 526-534	VRRAFPHCL	Negative

**NC5**				B*14:02	X 45-53	VSTDHGAHL	Negative
				B*15:01	Envelope 263-272	YQGMLPVCPL	Negative
				B*15:01	Polymerase 653-662	KQAFTFSPTY	Negative
				B*15:17	Envelope 167-175	ITSGFLGPL	Negative
				B*27:05	Polymerase 317-326	FRNSKPCSDY	Negative
				B*49:01	Core 112-122	RETVIEYLVSF	Negative
				B*49:01	Polymerase 92-101	CEQFVGPLTV	negative
				B*57:01	Polymerase 471-479	CSRNLYVSL	negative
