# Supplementary material for: Robust HLA-B-restricted CD8+ T-cell responses in chronic HBV infection
Source: JHEP Rep. 2026 Apr 24;8(8):101868. doi: 10.1016/j.jhepr.2026.101868 (PMC13351134; doi:10.1016/j.jhepr.2026.101868)
Supplement: Multimedia component 2 [file mmc2.docx]

**JHEP Reports**

**CTAT methods**

Tables for a “Complete, Transparent, Accurate and Timely account” (CTAT) are now mandatory for all revised submissions. The aim is to enhance the reproducibility of methods.

- Only include the parts relevant to your study
- Refer to the CTAT in the main text as ‘Supplementary CTAT Table’
- Do not add subheadings
- Add as many rows as needed to include all information
- Only include one item per row

**If the CTAT form is not relevant to your study, please outline the reasons why:**

|  |
| --- |

- 1. **Antibodies**

| **Name** | **Citation** | **Supplier** | **Cat no.** | **Clone no.** |
| --- | --- | --- | --- | --- |
| Fixierbarer Viabilitätsfarbstoff eFluor™ 506 |  | Thermo Fisher | 65-0866-14 |  |
| BD™ CD8 APC |  | BD Biosciences | 345775 | RPA-T8 |
| Brilliant Violet 421™ anti-human CD4 Antibody |  | BioLegend | 300532 | RPA-T4 |
| BD FastImmune™ Ant-Human IFN-y (25723.11) FITC |  | BD Biosciences | 340449 | 25723.11 |
| BD™ ELISPOT Human IFN-γ ELISPOT Pair |  | BD Biosciences | 551873 |  |
| BD™ ELISPOT HRP Streptavidin for ELISPOT |  | BD Biosciences | 557630 |  |

- 1. **Cell lines**

| **Name** | **Citation** | **Supplier** | **Cat no.** | **Passage no.** | **Authentication test method** |
| --- | --- | --- | --- | --- | --- |
|  |  |  |  |  |  |

- 1. **Organisms**

| **Name** | **Citation** | **Supplier** | **Strain** | **Sex** | **Age** | **Overall n number** |
| --- | --- | --- | --- | --- | --- | --- |
|  |  |  |  |  |  |  |

- 1. **Sequence based reagents**

| **Name** | **Sequence** | **Supplier** |
| --- | --- | --- |
| HBV_Pol_1extF | CACCTCTGCCTAATCATCTCTTGT | invitrogen |
| HBV_Pol_1extR | CTTGAGCAGGAGTCGTGCAGGT | invitrogen |
| HBV_Pol_1F | TGACTCTAGCTACCTGGGTG | invitrogen |
| HBV_Pol_1R | AACGGGCAACATACCTTGAT | invitrogen |
| HBV_Pol_2extF | GTGGCTCCAGTTCAGGAACAGTA | invitrogen |
| HBV_Pol_2extR | CTACAGCCTCCTAATACAAAGACCT | invitrogen |
| HBV_Pol_2F | GAACATGGAGAACATCACATCAG | invitrogen |
| HBV_Pol_2R | CTCAAGGTCGGTCGTTGACATT | invitrogen |

- 1. **Biological samples**

| **Description** | **Source** | **Identifier** |
| --- | --- | --- |
|  |  |  |

- 1. **Deposited data**

| **Name of repository** | **Identifier** | **Link** |
| --- | --- | --- |
|  |  |  |

- 1. **Software**

| **Software name** | **Manufacturer** | **Version** |
| --- | --- | --- |
| Flow Jo | BD Biosciences | 10.9.0 |
| BD FACSDiva™ | BD Biosciences | 8.0 |
| GraphPad Prism 9 | GraphPad | 10 |
| Immunospot Switchboard 2.7.4 | Cellular Technology Limited CTL | 2.7.4 |

- 1. **Other (e.g. drugs, proteins, vectors etc.)**

|  |  |  |
| --- | --- | --- |
|  |  |  |

- 1. **Please provide the details of the corresponding methods author for the manuscript:**

| Dr. Julia Lang-Meli  Department of Gastroenterology and Hepatology  University Hospital Cologne  Kerpener Strasse 62  50937 Cologne  Germany  Julia.lang-meli@uk-koeln.de |
| --- |

**2.0 Please confirm for randomised controlled trials all versions of the clinical protocol are included in the submission. These will be published online as supplementary information.**

|  |
| --- |
