# Supplementary material for: Robust HLA-B-restricted CD8+ T-cell responses in chronic HBV infection
Source: JHEP Rep. 2026 Apr 24;8(8):101868. doi: 10.1016/j.jhepr.2026.101868 (PMC13351134; doi:10.1016/j.jhepr.2026.101868)
Supplement: Multimedia component 4 [file mmc4.pdf]

JHEP|Reports

Robust HLA-B-restricted CD8+ T-cell responses in chronic HBV infection

Authors

Julia Lang-Meli, Anna-Lena Denecke, Johannes Ptok, …, Robert Thimme, Maike Hofmann, Christoph Neumann-Haefelin

Correspondence

robert.thimme@uniklinik-freiburg.de (R. Thimme), maike.hofmann@uniklinik-freiburg.de (M. Hofmann), christoph.neumann-haefelin@uk-koeln.de (C. Neumann-Haefelin).

Graphical abstract

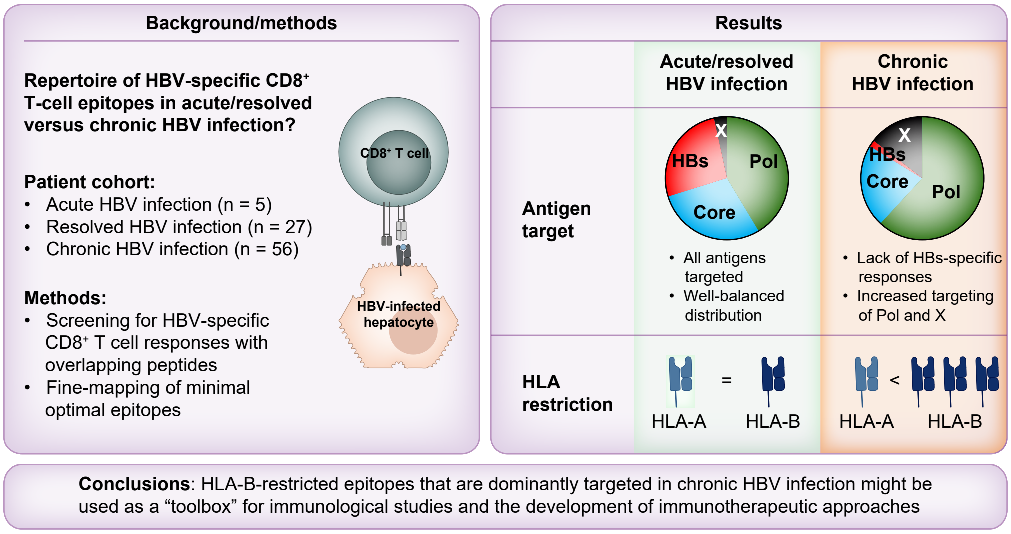

Highlights:

- “Toolbox” of 28 novel HBV-specific CD8+ T-cell epitopes primarily targeted in chronic HBV infection.
- Increased targeting of HBV pol- and X-derived CD8+ T-cell epitopes in chronic *vs.* acute/resolved HBV infection.
- Dominance of functional HLA-B-restricted epitopes in chronic *vs.* acute/resolved HBV infection.

Impact and implications:

To date, there is no treatment to achieve functional cure of chronic HBV infection. Immunotherapy boosting dysfunctional HBV-specific CD8+ T cells is an interesting approach, but relies on selection of optimal target epitopes. Using an unbiased approach, we found that the HBV-specific CD8+ T-cell epitope repertoire in chronic HBV infection is not well covered by previously described epitopes. We characterized 28 novel, primarily HLA-B-restricted epitopes that are dominantly targeted in chronic HBV infection. These might be used as a “toolbox” for immunological studies and immunotherapeutic approaches.

https://doi.org/10.1016/j.jhepr.2026.101868

© 2026 The Author(s). Published by Elsevier B.V. on behalf of European Association for the Study of the Liver (EASL). This is an open access article under the CC BY license (http://creativecommons.org/licenses/by/4.0/).

JHEP|Reports

Robust HLA-B-restricted CD8+ T-cell responses in chronic HBV infection☆

Julia Lang-Meli[1,2,3], Anna-Lena Denecke[1,4], Johannes Ptok[5], Philipp Ehrenmann[1], Elahe Salimi Alizei[1], Hendrik Luxenburger[1], Michelle Maas[1,4], Muthamia Kiraithe[1], Felix Jacobi[6], Giuseppe Rusignuolo[1], Isabel Schulien[1], Emma Gostick[7], Sian Llewellyn-Lacey[7], Florian Emmerich[8], Bertram Bengsch[1], Tobias Boettler[1], David A. Price[7,9], Andreas Walker[5], Jörg Timm[5], Robert Thimme[1,*], Maike Hofmann[1,*], Christoph Neumann-Haefelin[1,2,3,*]

JHEP Reports **2026**. vol. 8 | 1–10

Check for updates

Background & Aims: The function and quantity of CD8+ T-cell responses is superior in acute *vs.* chronic HBV infection. However, whether different HBV-derived epitopes are targeted in these distinct courses of HBV infection remains unclear.

Methods: We screened peripheral blood mononuclear cells from 56 patients with chronic HBV infection genotype D and 32 patients with acute/resolved HBV infection to identify responses to overlapping peptides (18mers) covering the full HBV genotype D proteome. We then performed experimental fine-mapping of the minimal optimal epitopes.

Results: Patients with acute HBV infection showed a broad HBV-specific CD8+ T-cell epitope repertoire. After spontaneous resolution, the strength of HBV-specific CD8 T-cell responses decreased, whereas the broad epitope landscape was preserved. In chronic HBV infection, the specificity of HBV-specific CD8+ T-cell responses to HBV antigens was shifted, with a lack of functional HBsAg-specific CD8+ T-cell responses (p = 0.0007), as recently described. Interestingly, patients with chronic HBV infection showed significantly more HLA-B-restricted HBV-specific CD8+ T-cell responses than HLA-A-restricted responses (33 *vs.* 19), whereas this distribution was balanced in patients with acute/resolved HBV infection (16 *vs.* 27; p = 0.0364). Most (79.6%) of the detected responses showed conserved autologous viral sequences. This observation was confirmed in a broader sequence dataset with no evidence for HLA-B-driven CD8+ T-cell selection pressure.

Conclusions: In contrast to acute/resolved infection, conserved HLA-B-restricted epitopes are dominant in chronic HBV infection, making them interesting candidates for immunotherapeutic approaches towards functional cure of chronic HBV infection.

© 2026 The Author(s). Published by Elsevier B.V. on behalf of European Association for the Study of the Liver (EASL). This is an open access article under the CC BY license (http://creativecommons.org/licenses/by/4.0/).

Introduction

Chronic HBV infection is a major global health burden accounting for >750,000 deaths annually. Most of these result from HBV-associated complications such as liver cirrhosis and hepatocellular carcinoma. Despite the availability of a safe and effective vaccine, the incidence of HBV infection remains high in the African and South East Asian region.[1,2] For patients with chronic HBV infection, treatment achieving functional cure is still lacking. One promising strategy is to enhance the HBV-specific CD8+ T-cell response by immunotherapeutic approaches like therapeutic vaccination.[3] The development of such therapies requires a detailed understanding of the HBV-specific CD8+ T-cell response. The choice of the ideal target antigen/epitopes is crucial in this context.[4] However, which epitopes are targeted in acute/resolved *vs.* chronic HBV infection remains incompletely understood. This open aspect in HBV immunobiology is of special importance, as most HBV-specific CD8+ T-cell epitopes have been identified in acute/resolved HBV infection, indicating a substantial bias when these epitopes are used to analyze HBV-specific CD8+ T-cell immunity in chronic HBV infection. More importantly, targeting these epitopes by immunotherapeutic approaches in chronic HBV infection may be of limited benefit. We thus addressed this knowledge gap with a detailed unbiased analysis of the HBV-specific CD8+ T-cell landscape in age- and HLA-matched patients with HBV infection of different courses.

Materials and methods

Study cohort

In this study, 56 patients with chronic HBV infection (all HBeAg negative and without evidence for liver cirrhosis, nucleos(t)ide

☆ Given their role as Associate Editor, Tobias Boettler had no involvement in the peer-review of this article and had no access to information regarding its peer-review. Full responsibility for the editorial process for this article was delegated to the Editor in Chief Josep M. Llovet.

* Corresponding authors. Addresses: Department of Medicine II, Freiburg University Medical Center, Hugstetter Strasse 55, 79106 Freiburg, Germany (R. Thimme or M. Hofmann), or Department of Gastroenterology and Hepatology, University Hospital Cologne, Kerpener Strasse 12, 50937 Cologne, Germany (C. Neumann-Haefelin).
E-mail addresses: robert.thimme@uniklinik-freiburg.de (R. Thimme), maike.hofmann@uniklinik-freiburg.de (M. Hofmann), christoph.neumann-haefelin@uk-koeln.de (C. Neumann-Haefelin).

https://doi.org/10.1016/j.jhepr.2026.101868

analogs-treated if clinically indicated), five patients with acute HBV infection, and 27 patients with serological evidence for resolved HBV infection were included. All patients were recruited at the Freiburg University Medical Center, Freiburg, Germany. HLA typing was performed using next-generation sequencing. Donor characteristics are summarized in Table S1.

Ethics

Written informed consent was obtained from all participants before inclusion. The study was conducted according to federal guidelines and local ethics committee regulations (Albert-Ludwigs-Universität, Freiburg, Germany; vote: 383/19, 322/20, 21-1135, and 315/20) and the Declaration of Helsinki (1975).

Isolation of PBMCs

Peripheral blood mononuclear cells (PBMCs) were isolated, stored, and thawed as described previously.[5]

Peptides

We synthesized 225 overlapping peptides (OLPs) spanning the whole HBV proteome genotype D (*ayw* subtype, GenBank accession number: X02496) as 18mers overlapped by 11 amino acids and containing free amine NH2 and COOH termini. These peptides were produced by Genaxxon Bioscience (Ulm, Germany) with a purity >70%.

In vitro expansion and intracellular IFN-γ staining with OLPs

We conducted *in vitro* expansion of PBMCs as described previously.[6] On Day 10, we screened for OLP-specific interferon gamma (IFN-γ) production via enzyme-linked immunospot (ELISpot) assay after 24-h stimulation with peptide pools in a 45 × 10 matrix setup. Each OLP was included in two peptide pools of the matrix (5 μM, 5 × 10^4 cell/well) with two positive wells defining one positive individual OLP. ELISpot was performed following manufacturer's instructions (BD Biosciences, Franklin Lakes, NJ, USA; 551951, 551873, 557630, and 551958), with the following concentrations: 0.05 μl/ml capture antibody, 0.04 μl/ml detection antibody, 0.1 μl/ml streptavidin-horseradish peroxidase, 0.02 μl/ml AEC chromogen. Positive wells in ELISpot were defined with >2× mean spot forming units of the negative controls (triplicate). The identified individual IFN-y-producing OLPs were subsequently validated on Days 12–14 by intracellular cytokine staining (ICCS) and flow cytometry. In particular, cells were restimulated with individual OLPs (5 μM), dimethyl sulfoxide as negative control or phorbol 12-myristate 13-acetate and ionomycin as positive control in the presence of brefeldin A and IL-2. After 5 h of incubation at 37 °C, cells were stained for surface markers (CD8+, CD4+; 7-amino-actinomycin D) and intracellular markers (IFN-γ). To test for specificity of the HBV OLP approach, four healthy donors were tested as described above without any detection of CD8+ T-cell responses.

"Fine-mapping" of minimal optimal epitopes

We used the Immune Epitope Database (IEDB) to screen viral amino acid sequences of positive individual OLPs in ICCS for previously described minimal epitopes matching the patient's HLA type. If no previously described epitope was found, we predicted candidates using two prediction algorithms ANN 4.0 and NetMHCpan EL 4.123 for 8-mer, 9-mer, and 10-mer peptides with half-maximal inhibitory concentration (IC_{50}) of <500 nM. These were subsequently tested in the respective patient by ICCS.

Experimental determination of HLA restriction

Four partially HLA-matched EBV-transformed B-lymphoblastoid cell lines (B-LCLs), sharing only one HLA class I allele with the HBV-specific effector CD8 T cell along with three irrelevant HLA types, were selected. B-LCLs were pulsed with the epitope peptide overnight, then washed six times, and used for stimulation (ICCS) of the epitope-specific T-cell line from the respective patient.

Flow cytometry for T-cell analysis

Analyses were performed on FACSCanto II^{TM} with FACSDiva software version 10.6.2 (BD Biosciences) or CytoFLEX (Beckman Coulter, Brea, CA, USA) with CytExpert software version 2.3.0.84 (Beckman Coulter Inc, Brea, CA, USA) after fixation of cells in 2% paraformaldehyde (Sigma). Data were then analyzed with FlowJo 10.7.1 (Tree Star Inc., Ashland, OR, USA). The gating strategy is displayed in Fig. S4.

Amplification and sequence analysis of the HBV genome

Autologous viral sequences of patients with CD8+ T-cell responses were obtained by Sanger sequencing (Eurofins Scientific SE, Luxemburg, Luxemburg) after purification of viral DNA from patient plasma using the QIAamp DNA Blood Mini Kit (Qiagen) and amplification via nested PCR and primers (Table S4).

Detection of HLA-associated mutations in CD8+ T-cell epitopes with HAMdetector

We analyzed a set of 239 previously published[7] sequences of patients with known HLA alleles (all HBV genotype D; all HBeAg negative) using HAMdetector[8] for HLA-associated viral sequence polymorphisms within the targeted HBV-specific CD8+ T-cell epitopes. The program is implemented as a Julia package for identifying HLA-associated substitutions based on aligned viral protein sequences paired to host HLA class I data. It integrates information from epitope prediction via MHCflurry 2.0 and genome phylogeny (based on RAxML-NG). The complete source code and documentation is available on GitHub online platform.[9]

MEME

Sites in the epitopes that evolved under positive selection were detected with the maximum likelihood approach *mixed effects model of evolution* (MEME) on the Datamonkey server with default parameters.[10,11]

SeqFeatR

In addition to HAMdetector analysis, HLA-associated viral sequence polymorphisms of all HBV proteins were also tested

with the R package SeqFeatRas previously described (R Foundation for Statistical Computing, Vienna, Austria).[12]

Statistical analysis

We performed statistical analysis using non-parametric tests with Prism 9 (GraphPad Software, San Diego, CA, USA). A *p* value <0.05 was considered as statistically significant.

Results

Differential T-cell response in acute/resolved *vs.* chronic infection

To map the HBV-specific CD8+ T-cell response in detail, we tested patients with acute (n = 5), resolved (n = 27), or age- and HLA-matched chronic HBV infection (n = 56, all HBeAg negative; Table S1; Fig. S1A and B) for responses to pools of OLPs spanning the whole HBV proteome using ELISpot assay. Positive pools were evaluated for the triggering individual OLPs by flow cytometry after intracellular IFN-γ staining (ICCS) (Fig. 1A). In line with previous reports,[13] we found fewer CD8+ T-cell responses to OLPs per patient in patients with chronic compared with acute HBV infection (Fig. 1B). Patients with resolved HBV infection also showed fewer CD8+ T-cell responses to OLPs compared with patients with acute HBV infection. Furthermore, the number of detected T-cell responses per patient was not different in resolved versus chronic HBV infection (Fig. 1B). All patients with acute HBV infection showed at least one HBV-specific CD8+ T-cell response to OLPs; however, we did not find HBV-specific CD8+ T-cell responses to any OLPs in about half of patients with resolved or chronic HBV infection (Fig. 1B). In therapy-naïve patients with chronic HBV infection, the sum of HBV-specific IFN-γ CD8+ T-cell responses per patients was not significantly correlated with viral load (Fig. 1C).

For each positive individual OLP, we searched the IEDB website (https://www.iedb.org) for previously described

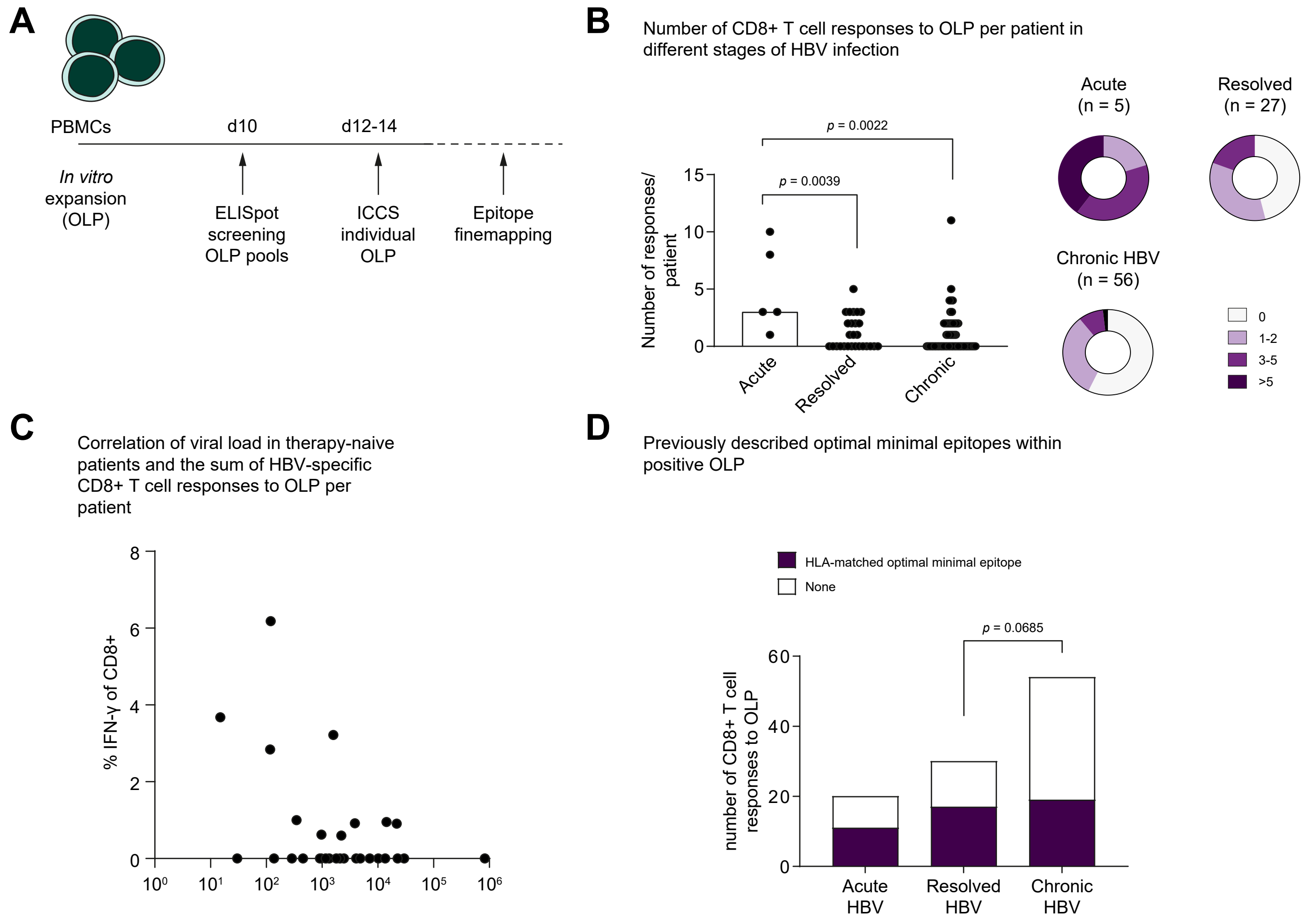

Fig. 1. Comprehensive fine-mapping of the CD8+ T-cell epitope repertoire in chronic *vs.* acute/resolved HBV infection. (A) Experimental setup. (B, left panel) Number of HBV-specific CD8+ T-cell responses to OLPs in patients with acute, resolved, or chronic HBV infection. Median is indicated and statistical analysis was performed with Kruskal-Wallis test with false-discovery rate correction using a step-up procedure of Benjamini, Krieger, and Yekutieli. (B, right panel) Proportion of CD8+ T-cell responses to OLPs in acute, resolved, or chronic HBV infection targeting different HBV proteins. (C) Correlation between the sum of HBV-specific CD8+ T-cell responses per patient and viral load in patients with chronic HBV infection. Statistical analysis was performed with Spearman correlation. (D) Percentage of positive OLPs with previously described optimal minimal epitope matching the respective patients' HLA type. Statistical analysis was performed with two-tailed Fisher's exact test. HLA, human leukocyte antigen; OLPs, overlapping peptides.

minimal optimal epitopes matching the patient's HLA type (Fig. 1D). Minimal optimal epitopes were identified more frequently with respect to T-cell responses from patients with acute (55%) or resolved HBV infection (56%) compared with chronic HBV infection (32.6%). As databases such as IEDB rely on previously identified epitopes, this suggests that the HBV-specific CD8+ T-cell epitope repertoire in chronic HBV infection remains understudied. This is inadequately represented by currently described HBV-specific CD8+ T-cell epitopes, which have primarily been identified in acute/resolved HBV infection.

Comprehensive fine-mapping of the CD8+ T-cell epitope repertoire in chronic *vs* acute/resolved HBV infection

We then aimed to address the identified knowledge gap of the optimal epitope repertoire in chronic HBV infection and comprehensively characterized the HBV-specific CD8+ T-cell epitope repertoire with a focus on chronic HBV infection. To do so, we performed *in silico* fine-mapping using two prediction methods (ANN and NetMHCpan on the IEDB webpage) for positive OLPs to predict HLA class I restriction and optimal epitope sequence and length based on the HLA class I alleles expressed by the respective patient. The epitope candidates were subsequently experimentally further validated by ICCS (Fig. 2A). For a subset of responses with inconclusive results from prediction, we also used partially HLA-matched immortalized B cell lines to experimentally assess HLA restriction of the CD8+ T-cell response to the OLP or minimal epitope (Fig. S1C). In total, HLA restriction and optimal epitope sequence were previously described or experimentally determined for 83 of the 104 (79.8%) CD8+ T-cell responses. For the remaining 21 CD8+ T-cell responses, no previously described HLA-matched optimal epitope was available, and experimental fine-mapping was not possible because of limited sample availability. In these cases, the best *in silico* predicted candidate was inferred. Specificity of assessed HLA restriction was further confirmed by HLA-mismatch experiments. PBMCs of five patients with chronic HBV infection were tested after *in vitro* expansion with all epitopes targeted in our study not matching their respective HLA type (Table S5). Epitopes restricted by HLA types belonging to the same HLA supertype family[14] as the patient's HLA type were excluded for possible cross-recognition. With this approach, overall 176 staining were negative. We found only one weak (0.16% IFN-γ+ of CD8+) CD8+ T-cell response to epitope B*18/Core7 (KEFGATVEL) from a patient with HLA alleles A*01:01; A*02:01; B*35:03; B*51:01. A detailed list of all positive HBV-specific CD8+ T-cell responses to OLPs with the respective fine-mapping of the minimal optimal epitope is provided in Table S2. With this approach, we were able to experimentally fine-map and characterize 28 novel HBV-specific CD8+ T-cell epitopes (Table S3).

We were further able to compare the HBV-specific CD8+ T-cell epitope repertoire in different cohorts on the population level (Fig. 2B, acute patients are presented individually in Fig. S2). Interestingly, the landscape of HBV-specific CD8+ T-cell responses showed a similar pattern in acute/resolved HBV infection. In contrast, the epitope landscape in chronic HBV infection was different compared to acute/resolved HBV infection.

HBV-specific CD8+ T-cell responses target HBV antigens differentially in chronic *vs*. acute/resolved HBV infection

In line with previous reports,[15–17] we found a broad HBV-specific CD8+ T-cell epitope repertoire in patients with acute/resolved HBV infection targeting different HBV antigens (Fig. 2B and C). Patients with chronic HBV infection showed fewer surface-specific CD8+ T-cell responses to OLPs than patients with acute/resolved HBV infection, as recently described.[18] We found a trend towards more polymerase- and HBx-specific CD8+ T-cell responses in patients with chronic HBV infection, while the HBV core antigen was targeted by a similar percentage of CD8+ T-cell responses from patients with acute, resolved, and chronic HBV infection (Fig. 2C). Comparing the strength of HBV-specific CD8+ T-cell responses targeting different HBV antigens, we found no significant differences (Fig. 2D).

Resolution of chronic HBV infection represents a rare event.[19] To complement our observations in acute, resolved, and chronic infection, we screened a small number of patients (n = 4) after resolution of chronic infection for their HBV-specific CD8+ T-cell repertoire (Tables S1 and S2). Two of four patients showed at least one HBV-specific CD8+ T-cell response. Interestingly, the overall three HBV-specific CD8+ T-cell responses were located in polymerase and X protein, and targeted new epitopes. Importantly, similar to the findings in our cohort with chronic HBV infection, none of the responses was located in HBsAg.

Dominance of conserved HLA-B-restricted CD8+ T-cell epitopes in chronic HBV infection

Regarding HLA restriction, patients with chronic HBV infection showed more HLA-B-restricted CD8+ T-cell responses, whereas the distribution in patients with acute/resolved HBV infection was quite balanced (Fig. 3A). This was also true when analyzing the data per patient with a significant (p = 0.0318) enrichment of patients targeting only HLA-B-restricted CD8+ T-cell responses in patients with chronic HBV infection compared with the combined cohorts with acute/resolved HBV infection (Fig. S3A). In patients with chronic HBV infection, HLA-B-restricted CD8+ T-cell responses were restricted by overall seven HLA types. HLA-B*07 and HLA-B*35 were the most frequently detected HLA restrictions. In patients with acute/resolved HBV infection, HLA-B-restricted CD8+ T-cell responses were restricted overall by eight HLA types; HLA-B*15, HLA-B*35, and HLA-B*40 were the most frequently detected HLA restrictions (Fig. S3B). Importantly, there was no significant enrichment of HLA-B*07 or HLA-B*35 in patients with chronic HBV infection (Fig. S1B). To confirm the dominance of the identified HLA-B-restricted CD8+ T-cell epitopes, we tested them in additional HLA-matched patients with chronic HBV infection after peptide-specific expansion with the minimal optimal epitope (Fig. S3C). The epitopes were dominant, with 12 of 20 HLA-B-restricted epitopes targeted in >20% of patients and seven of 20 epitopes in >40% of patients (Fig. S3C). Notably, similar to the findings obtained with OLPs, HLA-B*35 was the most frequently detected HLA restriction, followed by HLA-B*07. In patients with chronic HBV infection, there was a trend towards more vigorous HLA-B- *vs.* HLA-A-restricted CD8+ T-cell responses; however, this trend was not statistically significant (Fig 3B). Of note, both HLA-A and

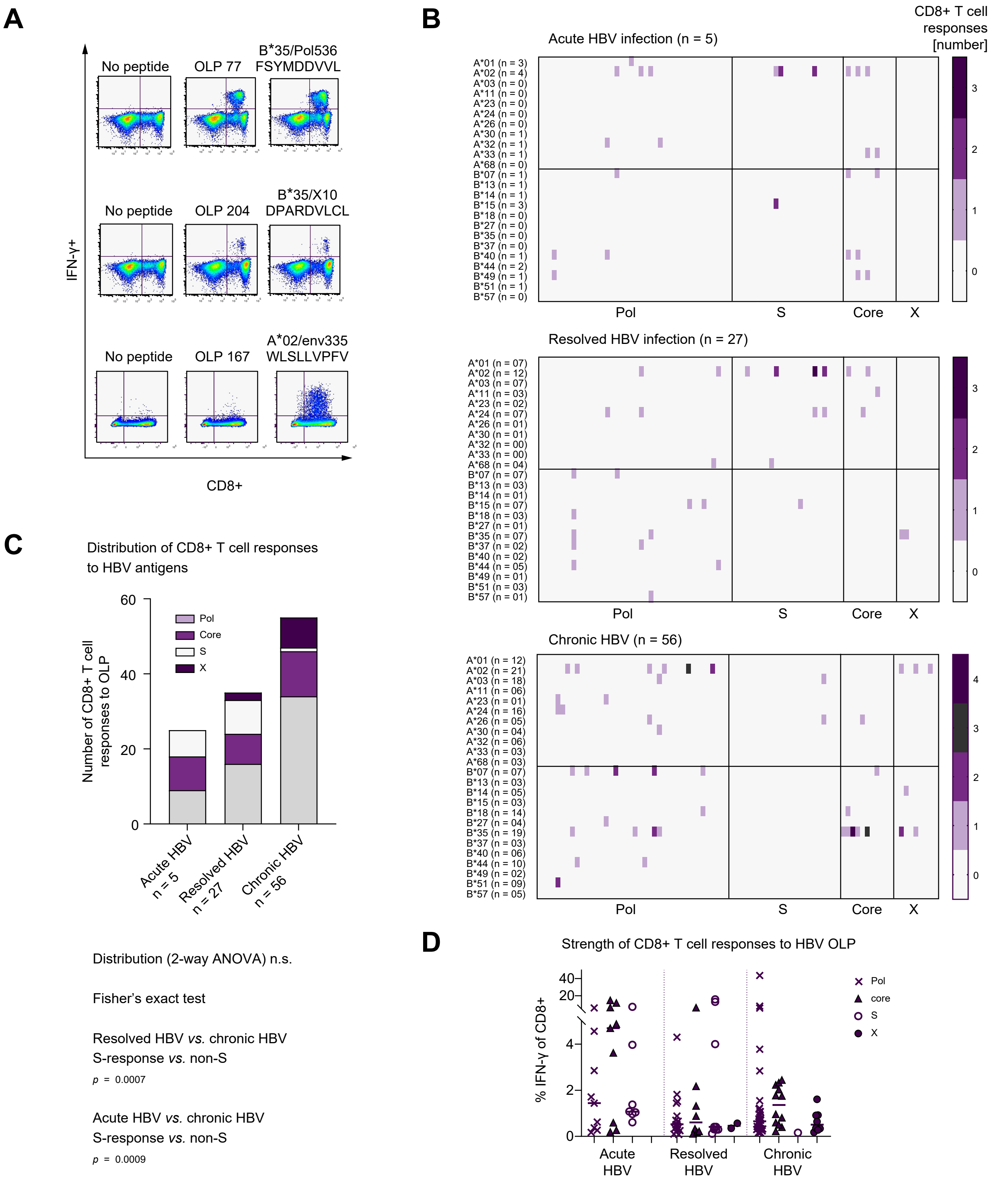

Fig. 2. Shift of antigen target of HBV-specific CD8+ T-cell responses in chronic *vs.* acute/resolved HBV infection. (A) Representative staining of experimental fine-mapping of minimal optimal epitope within positive OLPs. (B) Number, location, and HLA restriction of HBV-specific CD8+ T-cell responses to OLPs spanning the whole HBV proteome in patients with acute, resolved, or chronic HBV infection. (C) Percentage of HBV-specific CD8+ T-cell responses targeting different HBV proteins in acute/resolved *vs.* chronic HBV infection. (D) Strength of HBV-specific CD8+ T-cell responses to HBV OLPs targeting different HBV proteins. (E) Statistical analysis was performed with two-way ANOVA with false-discovery rate correction for multiple comparisons using a step-up procedure of Benjamini, Krieger, and Yekutieli or as indicated. OLPs, overlapping peptides.

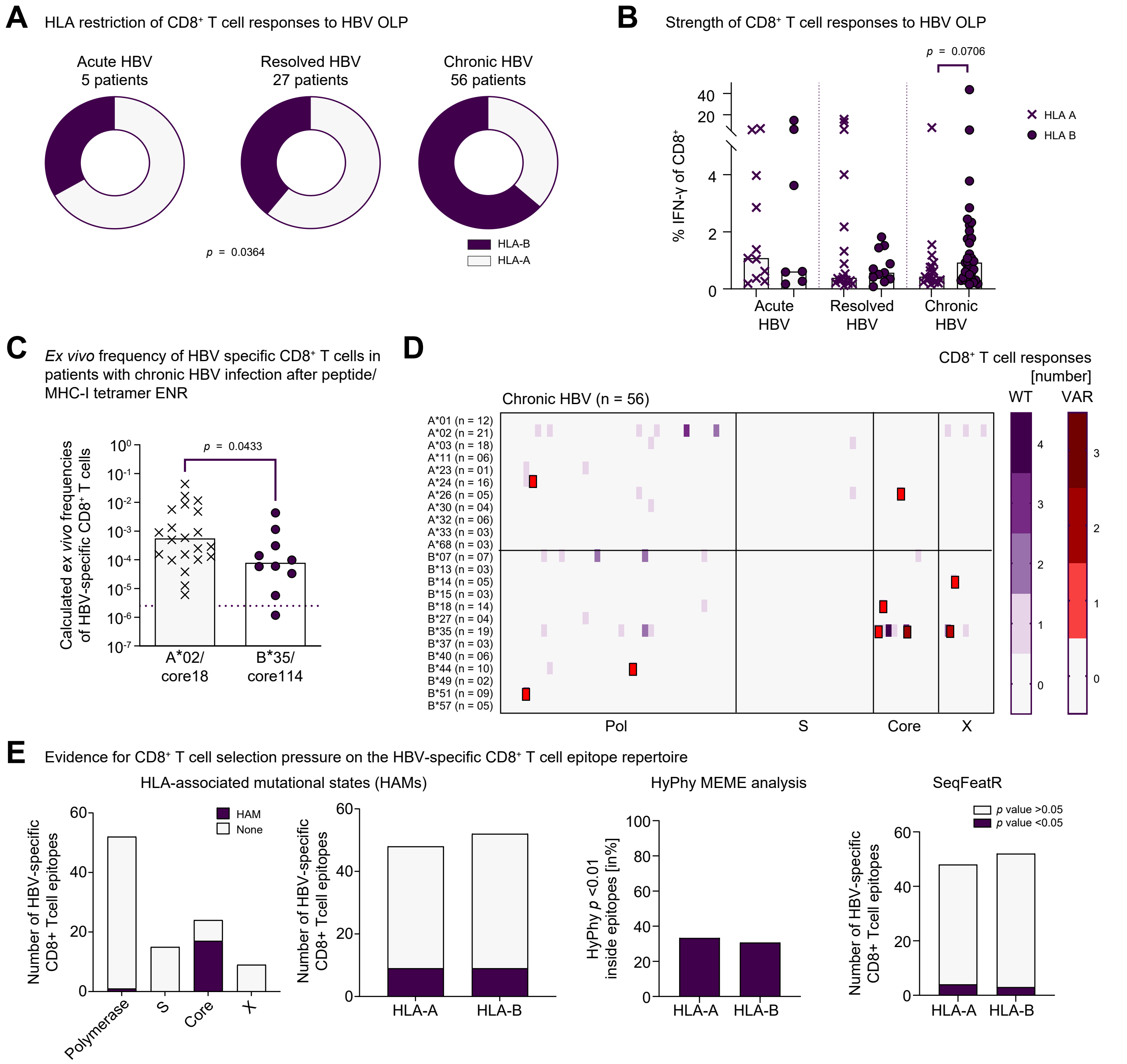

Fig. 3. Dominance of HLA-B-restricted CD8+ T-cell epitopes in chronic HBV infection. (A) Percentage of HBV-specific CD8+ T-cell responses restricted by HLA-A *vs.* HLA-B in acute, resolved, or chronic HBV infection. Statistical analysis was performed with Fisher's exact test. (B) Strength of HLA-A- *vs.* HLA-B-restricted HBV-specific CD8+ T-cell responses to HBV OLPs. Statistical analysis was performed with Kruskal-Wallis test with false-discovery rate correction using a step-up procedure of Benjamini, Krieger, and Yekutieli. (C) Calculated *ex vivo* frequencies of HBV-specific CD8+ T cells targeting a representative HLA-A *vs.* HLA-B-restricted core-epitope in patients with chronic HBV infection. Statistical analysis was performed with two-tailed Mann-Whitney *U* test. (D) Number, location, and HLA restriction of HBV-specific CD8+ T-cell responses to OLPs spanning the whole HBV proteome in patients with chronic HBV infection. Epitopes with sequence variations within the autologous viral sequences from the respective patients are indicated in red. (E) Number of HBV-specific CD8+ T-cell epitopes with or without HLA-associated mutational states (HAMdetector) or with *vs.* without positions under T-cell selection pressure (HyPhy MEME analysis and SeqFeatR). Statistical analysis was performed with two-way ANOVA with false-discovery rate correction for multiple comparisons using a step-up procedure of Benjamini, Krieger, and Yekutieli or unpaired *t* test. HLA, human leukocyte antigen; MEME, mixed effects model of evolution; OLPs, overlapping peptides; ENR, enrichment.

HLA-B-restricted epitopes reached detectable (>0.1%) IFN-γ responses in titration experiments at the applied peptide concentration of 5 µM (Fig. S3D). Comparison of *ex vivo* frequencies of HBV-specific CD8+ T cells targeting a representative HLA-A- *vs.* HLA-B-restricted epitope, interestingly, revealed lower frequencies of CD8+ T cells targeting the HLA-B- compared with the HLA-A-restricted epitope (Fig. 3C). This indicates that the dominance of functional HLA-B-

restricted CD8+ T-cell epitopes in chronic HBV infection might not simply be explained by higher *ex vivo* frequencies.

Next, we analyzed the autologous viral sequences corresponding to CD8+ T-cell epitopes in patients with chronic HBV infection and a detectable immune response (Table S2). We found viral sequence variations in overall 11 of 54 (20.4%) HBV-specific CD8+ T-cell responses (Fig. 3D). Of these 11 variants, seven were previously described as escape variants on the basis of footprint analysis.[7] In two exemplary cases, viral escape was also experimentally demonstrated (Fig. S3E). The autologous variant of epitope B*14/X_{45-53} "VS**SGL**GAHL" in patient CH-13 was no longer recognized by patient's CD8+ T cells. The autologous variant of epitope B*35/X_{10-18} "D**T**ARDVLCL" in patient CH-3 and CH-24 was cross-recognized. However, because the mutation affects the HLA-binding motif of the epitope, we pulsed B-LCL matched for only B*35 with the respective patients with wild type and variant of epitope B*35/X_{10-18}. Indeed, the B-LCL pulsed with variant peptide prompted a weaker HBV-specific CD8+ T-cell response upon co-culture with patient's PBMCs, indicative of suboptimal binding of the variant to the HLA molecule as escape mechanism (Fig. S3E). There was no significant accumulation of sequence variations in HLA-B-restricted CD8+ T-cell responses compared with HLA-A-restricted responses. We then expanded the analysis to the HBV-specific CD8+ T-cell epitope repertoire identified across all cohorts and used a set of 239 previously published sequences of patients with known HLA alleles (all HBV genotype D; all HBeAg negative). All HBV-specific CD8+ T-cell epitopes targeted in our study were screened with a Bayesian model (HAMdetector) for identification of HLA-associated mutational states (HAMs).[8] The posterior probabilities for substitutions being HLA associated were quantified for each position in the targeted epitope in a range from 0 ("substitution is disfavored in the presence of the restricting HLA allele") to 1 ("substitution is favored in the presence of the restricting HLA allele"). High posterior probabilities with values >0.8 indicate that the amino acid is favored in individuals with this HLA class I allele, consistent with CD8 T-cell escape. For each epitope, the maximum posterior probability was determined and epitopes with at least one residue >0.8 were considered "HAM positive." In line with recent evidence,[7] most HAMs were located in the HBV core protein. The number of HAM-positive epitopes was similar in HLA-A- *vs.* HLA-B-restricted epitopes (Fig. 3E). This was also true when analyzing viral selection pressure in HLA-A- *vs*. HLA-B-restricted epitopes using alternative statistical methods (MEME analysis and SeqFeatR). Accordingly, general entropy analysis did not find differences in sequence variability in HLA-A- *vs.* HLA-B-restricted epitopes (Fig. S3F). Overall, the dominance of HLA-B-restricted HBV-specific CD8+ T-cell epitopes is not associated with T-cell selection pressure, making these epitopes interesting candidates for immunotherapeutic approaches.

Discussion

Our data provides an overview of the HBV-specific CD8+ T-cell landscape in different stages of HBV infection. The main finding is that the functional HBV-specific CD8+ T-cell epitope repertoire in chronic infection differs from that in acute/resolved HBV infection in several important aspects. Although our experimental setup is focused on functional repertoire and competitive effects, and the necessity for epitope processing from OLPs may limit the complete detection of all CD8+ T-cell responses, our data clearly supports the following conclusions:

(1) The antigen target of functional HBV-specific CD8+ T-cell responses is different in acute/resolved *vs.* chronic HBV infection. Indeed, HBsAg-specific CD8+ T-cell responses are largely absent in chronic HBeAg-negative HBV infection. In addition, polymerase-specific as well as X protein-specific CD8+ T-cell responses are enriched in chronic HBV infection (trend). This observation underlines the previous notion that the functional HBV-specific CD8+ T-cell epitope repertoire differs between acute/resolved *vs.* chronic infection. Le Bert *et al.*[18] observed, for example, rarely HBsAg-specific CD8+ T-cell responses in patients with chronic HBV infection aged >30 yr. This finding is in line with a low detection rate of HBsAg-specific CD8+ T cells by our group, even when using a highly sensitive tetramer-based enrichment approach.[20] Park *et al.*[21] also found a different distribution of HBV-specific CD8+ T-cell responses to HBV proteins in different clinical phases. The mechanisms resulting in the differential antigen targeting in chronic *vs.* acute/resolved HBV infection are not yet well defined. Long-term exposure to high antigen loads may contribute to exhaustion and deletion of HBsAg-specific CD8+ T cells. For the other HBV antigens, a growing body of evidence indicates that the targeted antigen may have an important impact on function and differentiation of HBV-specific CD8+ T cells.[5,20,22–24] Indeed, HBV-specific CD8+ T cells targeting different viral antigens are a heterogeneous population composed of subsets with different functional levels contributing to endogenous viral control in some patients. Although our results agree with previous reports testing responses to OLP pools, the small number of available samples with acute HBV infection and combination of acute and resolved cohorts for a few analyses is a limitation of our dataset. For patients with resolved HBV infection, time since viral clearance might also influence the vigor of detected CD8+ T-cell responses.[13] Unfortunately, precise clinical information on the time point of viral clearance/infection is only available for a small subgroup of our cohort with resolved HBV infection, hindering systematic stratified analysis.
(2) In patients with HBeAg-negative chronic HBV infection, the majority of detectable IFN-γ-producing HBV-specific CD8+ T-cell responses were restricted by HLA-B, whereas the majority of HBV-specific CD8+ T-cell responses in patients with acute/resolved HBV infection were restricted by HLA-A. Notably, the dominance of HLA-B-restricted CD8+ T-cell responses was largely driven by the common HLA-B types B*07 and B*35 and not limited to rare HLA-B types. This dominance of HLA-B does not translate into higher frequencies of HLA-B-associated mutational escape, as viral sequence variations occurred with similar frequency in both HLA-A- and HLA-B-restricted epitopes. This was true in our cohort with comprehensive data on the targeted epitopes as well as in a larger cohort with combined viral sequence and HLA data. To our knowledge, our study is the first to report the dominance of HLA-B-restricted

CD8+ T-cell responses in chronic HBV infection. This observation further supports the concept of differential HBV-specific CD8+ T-cell epitope repertoires in acute/resolved *vs.* chronic HBV infection. This observation agrees with findings in the context of other chronic viral infections such as HCV[25,26] and HIV,[27] where these responses are associated with viral control. Viral sequence variations consistent with viral escape occurred in 20% of targeted HBV-specific CD8+ T-cell epitopes. This is less frequent compared with, for example, HCV infection, where ~50–70% of virus-specific CD8+ T-cell responses are affected by viral escape.[28,29] This finding is consistent with previous concepts suggesting that viral escape in HBV infection is limited by overlapping reading frames of HBV proteins, thereby limiting mutational flexibility of HBV.[30] Of note, this finding may not necessarily translate to a setting of more replicative chronic HBV infection, where viral escape may occur more frequently. Data on this important issue are still lacking. However, in contrast to HCV and HIV, HLA-B does not seem to have a dominant role in driving viral evolution in HBV infection. HLA-B-restricted epitopes in chronic HBV infection might be more under constraint to retain their viral sequence due to overlapping reading frames. HLA-B-restricted HLA-binding patterns more often include amino acids with structural function (*e.g.* proline at position 2 for HLA-B*07 supertype, arginine at position 2 for HLA-B*27), whereas HLA-A-restricted epitopes generally allow more flexible amino acid residues at their binding anchors (*e.g.* HLA-A*02 supertype requires a small or aliphatic hydrophobic residue [A, I, V, L, M, or T] at the second amino acid position). The substitution of amino acids within HLA-B binding patterns therefore might be associated with higher viral fitness costs in the context of overlapping reading frames. Thus, it might be worthwhile to include these HLA-B-restricted epitopes as targets in immunotherapeutic approaches such as therapeutic vaccination. The mechanisms that may direct the dominance of HLA-B restriction in chronic HBV infection remain elusive. As the proportion of responses affected by viral escape mutations does not substantially differ between HLA-A and HLA-B, superior functional capacities of CD8+ T cells that no longer recognize their cognate epitope due to mutational escape and are less prone to T-cell exhaustion is unlikely to explain the dominance of HLA-B-restricted CD8+ T-cell responses. Of note, the abundance of HLA-B-restricted CD8+ T-cell responses in chronic HBV infection may be owing to the sensitivity of the method used and possible competitive effects, as there was a trend towards more vigorous HLA-B- than HLA-A-restricted CD8+ T-cell responses in chronic HBV infection but not in acute/resolved HBV infection. However, the dominance of functional HLA-B-restricted CD8+ T-cell epitopes in chronic HBV infection might not be explained simply by higher *ex vivo* frequencies (Fig. 3C). Further longitudinal studies need to clarify if the difference in HLA restriction of the functional HBV-specific CD8+ T-cell repertoire between chronic and acute/resolved HBV infection is dynamic (*e.g.* shifting in patients with acute-persisting infection from acute to chronic infection, or if patients with acute-resolving *vs.* chronic-persisting HBV infection primarily show a differential HBV-specific CD8+ T-cell epitope repertoire already in the acute phase of infection). Future studies also need to clarify if HLA-B- *vs.* HLA-A-restricted responses differ in cytotoxic capacity, proliferative potential, and phenotype.

(3) Consistent with a differential antigen targeting and HLA restriction, the functional HBV-specific CD8+ T-cell epitope repertoire in chronic HBV infection is not well covered by previously described HBV-specific CD8+ T-cell epitopes. These epitopes have been mostly identified in patients with acute/resolved HBV infection. This finding indicates that utilizing the previously described HBV-specific CD8+ T-cell epitopes from databases in immunology studies leads to a bias when analyzing the HBV-specific CD8+ T-cell epitope repertoire in chronic HBV infection. Furthermore, using these known HBV-specific CD8+ T-cell epitopes in immunotherapeutic approaches aiming at functional cure of chronic HBV infection may not be appropriate. We thus aimed to add to the knowledge of HBV-specific CD8+ T-cell epitopes that are targeted in chronic HBV infection. Importantly, we could fine-map 28 novel HBV-specific CD8+ T-cell epitopes that were targeted in chronic HBV infection. When defining the HLA restriction of these novel epitopes, in line with our previous observations, HLA-B clearly dominated. These novel epitopes may be used as a “toolbox” for further studies and also for target antigen/epitope design in the context of immunotherapeutic approaches. Utilizing the previously described HBV-specific CD8+ T-cell epitopes from databases in immunology studies instead might lead to a bias when analyzing the HBV-specific CD8+ T-cell epitope repertoire in chronic HBV infection. In addition, T-cell receptor-redirected CD8+ T cells targeting the novel epitopes in the X protein may be promising for the treatment of HBV-associated hepatocellular carcinoma.[31]

In this study, we demonstrated a differential epitope repertoire of acute and resolved *vs.* chronic HBV infection with respect to viral antigen target and HLA-A *vs.* HLA-B dominance. The HBV-specific CD8+ T-cell epitope repertoire in chronic HBV infection is not well covered by previously described HBV-specific CD8+ T-cell epitopes. These findings have important implications for the design of immunotherapeutic approaches towards functional cure of chronic HBV infection. Indeed, targeting the well-conserved HLA-B-restricted CD8+ T-cell epitopes by therapeutic vaccination, in combination with restoration and enhancement of pre-existing CD4+ and CD8+ T-cell responses,[32–34] may be an important therapeutic component for HBV infection.

Affiliations

[1]*Department of Medicine II (Gastroenterology, Hepatology, Endocrinology and Infectious Diseases), Freiburg University Medical Center, Faculty of Medicine, University of Freiburg, Freiburg, Germany;* [2]*Department of Gastroenterology and Hepatology, University Hospital Cologne, Faculty of Medicine, University of Cologne, Cologne, Germany;* [3]*Center for Molecular Medicine Cologne (CMMC), University of Cologne, Cologne, Germany;* [4]*Faculty of Biology, University of Freiburg, Freiburg, Germany;* [5]*Institute of Virology, Heinrich-Heine-University, University Hospital, Duesseldorf, Germany;* [6]*Department of Dermatology and Venereology, Freiburg University Medical Center, Faculty of Medicine, University of Freiburg, Freiburg, Germany;* [7]*Division of Infection and Immunity, Cardiff University School of Medicine, University Hospital of Wales, Cardiff, UK;* [8]*Institute for Transfusion Medicine and Gene Therapy, Freiburg University Medical Center, Faculty of Medicine, University of Freiburg, Freiburg, Germany;* [9]*Systems Immunity Research Institute, Cardiff University School of Medicine, University Hospital of Wales, Cardiff, UK*

Abbreviations

B-LCL, B-lymphoblastoid cell line; HAM, HLA-associated mutational state; HLA, human leukocyte antigen; ICCS, intracellular cytokine staining; IEDB, Immune Epitope Database; IFN-γ, interferon gamma; MEME, mixed effects model of evolution; OLPs, overlapping peptides; PBMCs, peripheral blood mononuclear cells.

Financial support

This study was supported by grants from the Deutsche Forschungsgemeinschaft (DFG, German Research Foundation; 272983813 to BB, TB, RT, MH, and CNH, and 256073931 to BB, RT, MH, and CNH; IMM-PACT Program for Clinician Scientists 413517907 to HL and JLM) and the Deutsches Zentrum für Infektionsforschung (DZIF, German Center for Infection Research; TTU Hepatitis to TB, RT, and CNH). JLM was further supported by an Else Kröner Memorial Fellowship from the Else Kroner-Fresenius Foundation. HL was further supported by the IMMediate Advanced Clinician Scientist-Program, Department of Medicine II, Medical Center – University of Freiburg and Faculty of Medicine, University of Freiburg, funded by the Bundesministerium für Bildung und Forschung (BMBF, Federal Ministry of Education and Research) - 01EO2103. MH was further supported by the Heisenberg program (DFG, German Research Foundation; HO 5836/2-1). RT was further supported by the European Union (EU H2020-847939-IP-cure-B). JP was supported by the German Federal Ministry of Education and Research (Bundesministerium für Bildung und Forschung; Netzwerk Universitätsmedizin, GenSurv/MolTraX 01KX2021). DAP was supported by a Wellcome Trust Senior Investigator Award (100326/Z/12/Z). The funding bodies had no role in the decision to write or submit the manuscript for publication.

Conflicts of interest

Please refer to the accompanying ICMJE disclosure forms for further details.

Authors' contributions

JLM, ALD, JP, PE, ESA, HL, MM, MK, FJ, and IS planned, performed, and analyzed experiments. GR, EG, SLL, FE, BB, TB, DAP, AW, and JT collected clinical data and provided critical reagents/resources. RT, MH, and CNH designed the study and contributed to experimental design and planning. JLM, RT, MH, and CNH. interpreted data and wrote the manuscript. All authors contributed intellectually and concurred with the decision to submit the work for publication.

Data availability

No data is deposited on public databases. All requests for raw and analyzed data and materials will be reviewed by the corresponding authors to verify if the request is subject to any confidentiality obligations. Patient-related data not included in the paper were generated as part of clinical examination and may be subject to patient confidentiality. Any data and materials that can be shared will be released via a material transfer agreement.

Acknowledgements

We thank all patients for participating in the study. The graphical abstract was created with BioRender.com.

Supplementary data

Supplementary data to this article can be found online at https://doi.org/10.1016/j.jhepr.2026.101868.

References

Author names in bold designate shared co-first authorship

[1] Devarbhavi H, Asrani SK, Arab JP, et al. Global burden of liver disease: 2023 update. J Hepatol 2023;79:516–537.
[2] Wong GLH, Lemoine M. The 2024 updated WHO guidelines for the prevention and management of chronic hepatitis B: main changes and potential implications for the next major liver society clinical practice guidelines. J Hepatol 2025;82:918–925.
[3] Ji Y, Le Bert N, Lai-Hung Wong G, et al. The impact of hepatitis B surface antigen reduction via small interfering RNA treatment on natural and vaccine (BRII-179)-induced hepatitis B virus-specific humoral and cellular immune responses. Gastroenterology 2025;169:136–149.
[4] Maini MK, Burton AR. Restoring, releasing or replacing adaptive immunity in chronic hepatitis B. Nat Rev Gastroenterol Hepatol 2019;16:662–675.
[5] Heim K, Binder B, Sagar, et al. TOX defines the degree of CD8+ T cell dysfunction in distinct phases of chronic HBV infection. Gut 2020;70:1550–1560.
[6] **Lang-Meli J**, **Luxenburger H**, **Wild K**, et al. SARS-CoV-2-specific T-cell epitope repertoire in convalescent and mRNA-vaccinated individuals. Nat Microbiol 2022;7:675–679.
[7] Schwarz T, Ptok J, Damagnez M, et al. HBV shows different levels of adaptation to HLA class I-associated selection pressure correlating with markers of replication. J Hepatol 2025;82:805–815.
[8] Habermann D, Kharimzadeh H, Walker A, et al. HAMdetector: a Bayesian regression model that integrates information to detect HLA-associated mutations. Bioinformatics 2022;38:2428–2436.
[9] Escape.jl. https://github.com/HAMdetector/Escape.jl Accessed 28 April 2026.
[10] Datamonkey server. https://www.datamonkey.org/Accessed 28 April 2026.
[11] Murrell B, Wertheim JO, Moola S, et al. Detecting individual sites subject to episodic diversifying selection. PLoS Genet 2012;8:e1002764.
[12] Kefalakes H, Budeus B, Walker A, et al. Adaptation of the hepatitis B virus core protein to CD8(+) T-cell selection pressure. Hepatology 2015;62:47–56.
[13] Kefalakes H, Jochum C, Hilgard G, et al. Decades after recovery from hepatitis B and HBsAg clearance the CD8+ T cell response against HBV core is nearly undetectable. J Hepatol 2015;63:13–19.
[14] Sidney J, Peters B, Frahm N, et al. HLA class I supertypes: a revised and updated classification. BMC Immunol 2008;9:1.
[15] Thimme R, Wieland S, Steiger C, et al. CD8(+) T cells mediate viral clearance and disease pathogenesis during acute hepatitis B virus infection. J Virol 2003;77:68–76.
[16] Rehermann B, Fowler P, Sidney J, et al. The cytotoxic T lymphocyte response to multiple hepatitis B virus polymerase epitopes during and after acute viral hepatitis. J Exp Med 1995;181:1047–1058.
[17] Ferrari C, Penna A, Bertoletti A, et al. Cellular immune response to hepatitis B virus-encoded antigens in acute and chronic hepatitis B virus infection. J Immunol 1990;145:3442–3449.
[18] Le Bert N, Gill US, Hong M, et al. Effects of hepatitis B surface antigen on virus-specific and global T cells in patients with chronic hepatitis B virus infection. Gastroenterology 2020;159:652–664.
[19] European Association for the Study of the Liver. EASL Clinical Practice Guidelines on the management of hepatitis B virus infection. J Hepatol 2025;83:502–583.
[20] Schuch A, Salimi Alizei E, Heim K, et al. Phenotypic and functional differences of HBV core-specific versus HBV polymerase-specific CD8+ T cells in chronically HBV-infected patients with low viral load. Gut 2019;68:905–915.
[21] Park JJ, Wong DK, Wahed AS, et al. Hepatitis B virus–specific and global T-cell dysfunction in chronic hepatitis B. Gastroenterology 2016;150:684–695.e5.
[22] Hoogeveen RC, Robidoux MP, Schwarz T, et al. Phenotype and function of HBV-specific T cells is determined by the targeted epitope in addition to the stage of infection. Gut 2019;68:893–904.
[23] **Heim K**, **Sagar**, Sogukpinar Ö, et al. Attenuated effector T cells are linked to control of chronic HBV infection. Nat Immunol 2024;25:1650–1662.
[24] Winkler F, Hipp AV, Ramirez C, et al. Enolase represents a metabolic checkpoint controlling the differential exhaustion programmes of hepatitis virus-specific CD8+ T cells. Gut 2023;72:1971–1984.
[25] Walker A, Skibbe K, Steinmann E, et al. Distinct escape pathway by hepatitis C virus genotype 1a from a dominant CD8+ T cell response by selection of altered epitope processing. J Virol 2016;90:33–42.
[26] Nitschke K, Barriga A, Schmidt J, et al. HLA-B*27 subtype specificity determines targeting and viral evolution of a hepatitis C virus-specific CD8+ T cell epitope. J Hepatol 2014;60:22–29.
[27] Berger CT, Frahm N, Price DA, et al. High-functional-avidity cytotoxic T lymphocyte responses to HLA-B-restricted Gag-derived epitopes associated with relative HIV control. J Virol 2011;85:9334–9345.
[28] Neumann-Haefelin C, Timm J, Spangenberg HC, et al. Virological and immunological determinants of intrahepatic virus-specific CD8+ T-cell failure in chronic hepatitis C virus infection. Hepatology 2008;47:1824–1836.

[29] Cox AL, Mosbruger T, Mao Q, et al. Cellular immune selection with hepatitis C virus persistence in humans. J Exp Med 2005;201:1741–1752.
[30] Rehermann B, Pasquinelli C, Mosier SM, et al. Hepatitis B virus (HBV) sequence variation of cytotoxic T lymphocyte epitopes is not common in patients with chronic HBV infection. J Clin Invest 1995;96:1527–1534.
[31] Wan X, Wisskirchen K, Jin T, et al. Genetically-modified, redirected T cells target hepatitis B surface antigen-positive hepatocytes and hepatocellular carcinoma lesions in a clinical setting. Clin Mol Hepatol 2024;30:735–755.
[32] Hoogeveen RC, Dijkstra S, Bartsch LM, et al. Hepatitis B virus-specific CD4 T cell responses differentiate functional cure from chronic surface antigen+ infection. J Hepatol 2022;77:1276–1286.
[33] Schmidt NM, Wing PAC, Diniz MO, et al. Targeting human Acyl-CoA: cholesterol acyltransferase as a dual viral and T cell metabolic checkpoint. Nat Commun 2021;12:2814.
[34] Urbanek-Quaing M, Chou YH, Gupta MK, et al. Enhancing HBV-specific T cell responses through a combination of epigenetic modulation and immune checkpoint inhibition. Hepatology 2025;82:739–754.

Keywords: Hepatitis B virus (HBV); T cell; Human leukocyte antigen (HLA); Major histocompatibility complex (MHC); Viral escape.
Received 23 December 2025; received in revised form 8 April 2026; accepted 14 April 2026; Available online 24 April 2026

Journal of Hepatology, Volume *8*

Supplemental information

Robust HLA-B-restricted CD8+ T-cell responses in chronic HBV infection

Julia Lang-Meli, Anna-Lena Denecke, Johannes Ptok, Philipp Ehrenmann, Elahe Salimi Alizei, Hendrik Luxenburger, Michelle Maas, Muthamia Kiraithe, Felix Jacobi, Giuseppe Rusignuolo, Isabel Schulien, Emma Gostick, Sian Llewellyn-Lacey, Florian Emmerich, Bertram Bengsch, Tobias Boettler, David A. Price, Andreas Walker, Jörg Timm, Robert Thimme, Maike Hofmann, and Christoph Neumann-Haefelin

Robust HLA-B restricted CD8+ T cell responses in chronic HBV infection

Julia Lang-Meli, Anna-Lena Denecke, Johannes Ptok, Philipp Ehrenmann, Elahe Salimi Alizei, Hendrik Luxenburger, Michelle Maas, Muthamia Kiraithe, Felix Jacobi, Giuseppe Rusignuolo, Isabel Schulien, Emma Gostick, Sian Llewellyn-Lacey, Florian Emmerich, Bertram Bengsch, Tobias Böttler, David A. Price, Andreas Walker, Jörg Timm, Robert Thimme, Maike Hofmann, Christoph Neumann-Haefelin

Table of contents

Fig. S1 ……………………………………………………………...………………...2

Fig. S2 ……………………………………………………………………………….…3

Fig. S3 ……………………………………………………………………...………...4

Fig. S4 ………………………………………...……………………………………6

Table S1 …………………………………………….………………………….……7

Table S2 …………………………………………...……………………….……..12

Table S3 ……………………………….……………………………….………...33

Table S4 ……………………………………………………………………………37

Table S5 …………………………………………………...…………….………..38

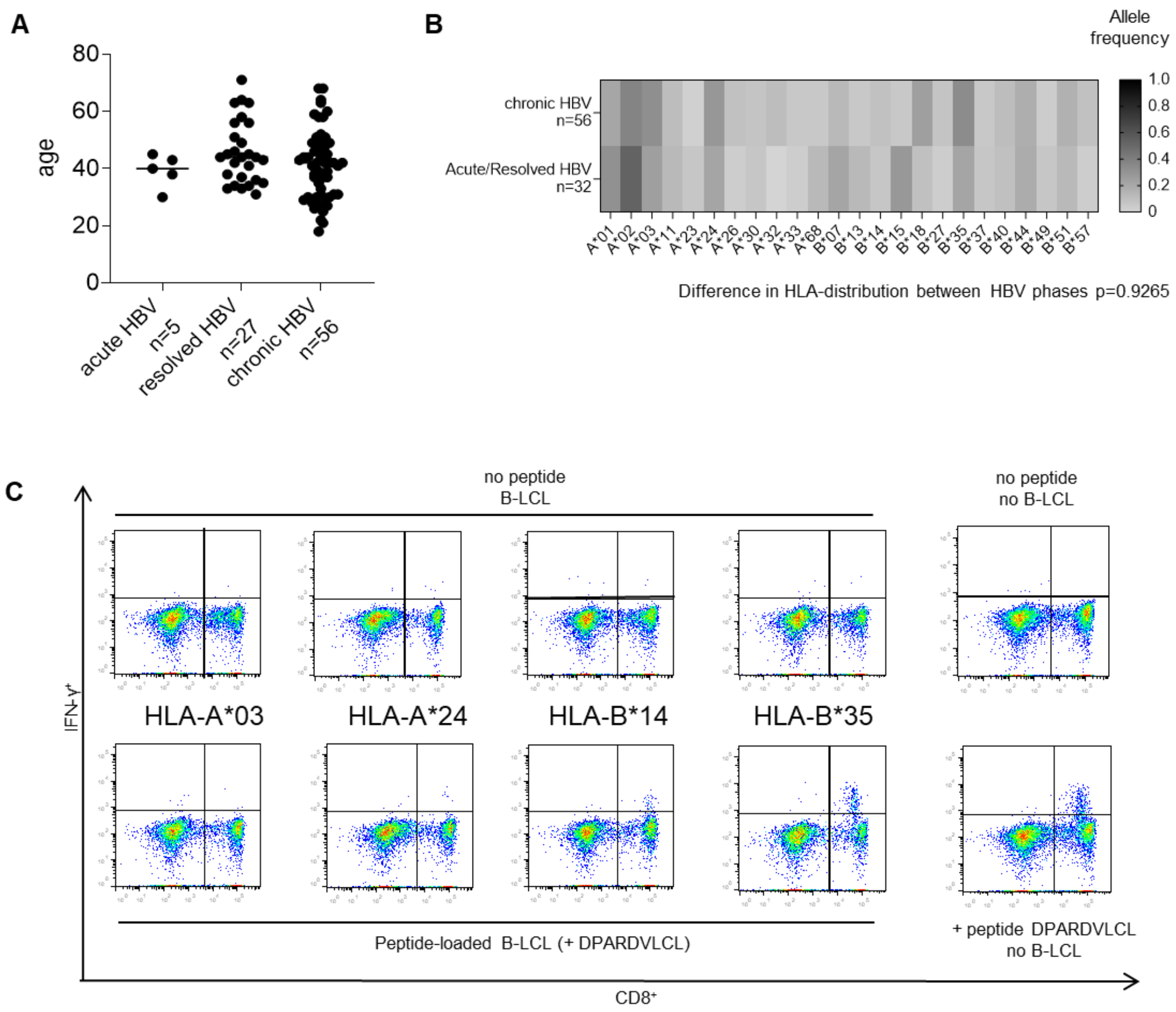

Fig. S1. Patient cohorts and representative staining

Age of patients (A) and frequency of different HLA types (B) in the patient cohorts. Representative staining for experimental determination of HLA-restriction using B-lymphoblastoid cell lines (B-LCL); patient CH-3 with response to B*35-restricted epitope DPARDVLCL (C). Statistical analysis was performed with Kruskal-Wallis test (A) or two-way ANOVA (B) with false-discovery rate correction using a step-up procedure of Benjamini, Krieger and Yekutieli.

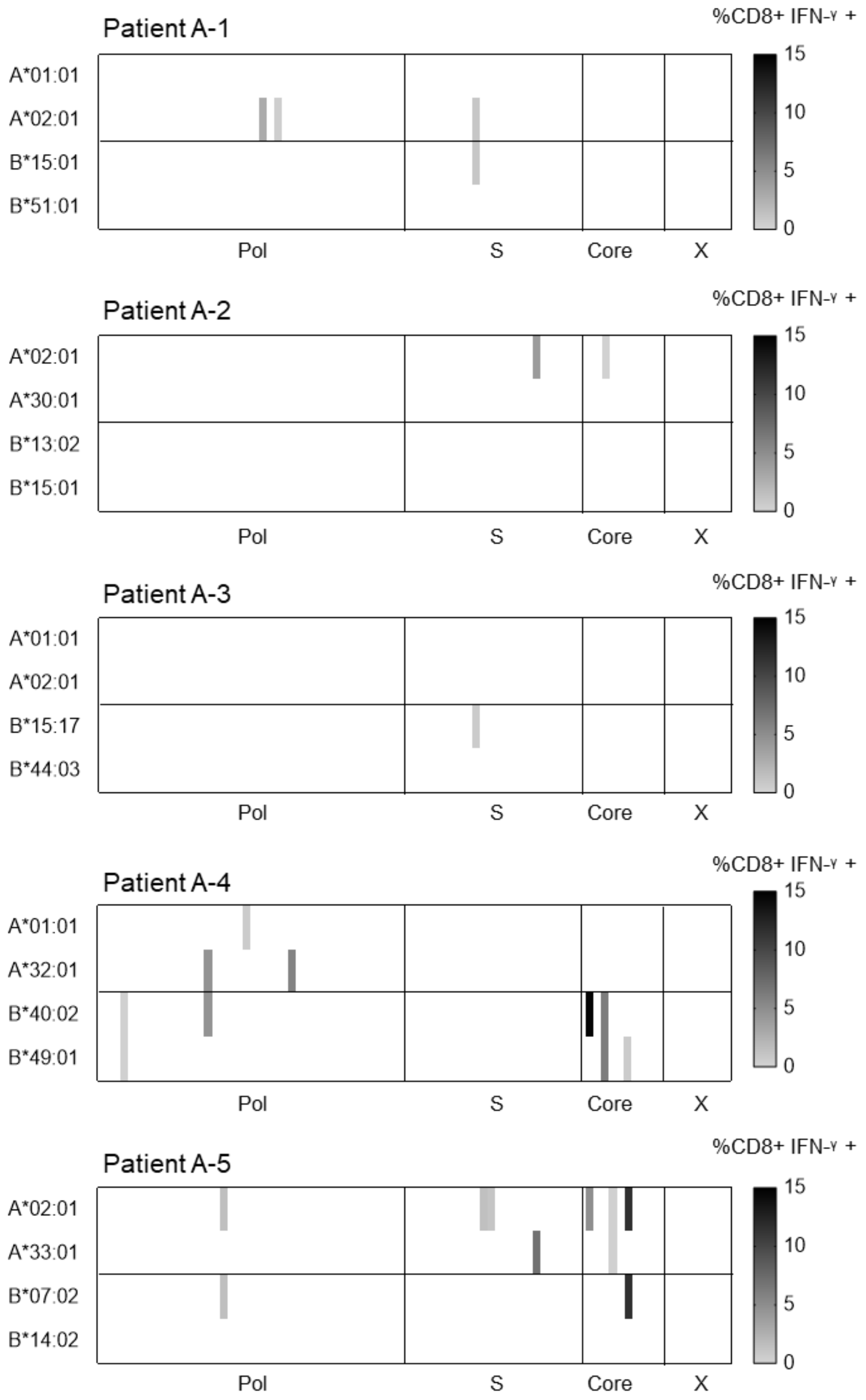

Fig. S2. HBV-specific CD8+ T cell repertoire in individual patients with acute HBV infection

Strength, location and HLA-restriction of HBV-specific CD8+ T cell responses to OLP spanning the whole HBV proteome in 5 patients with acute HBV infection

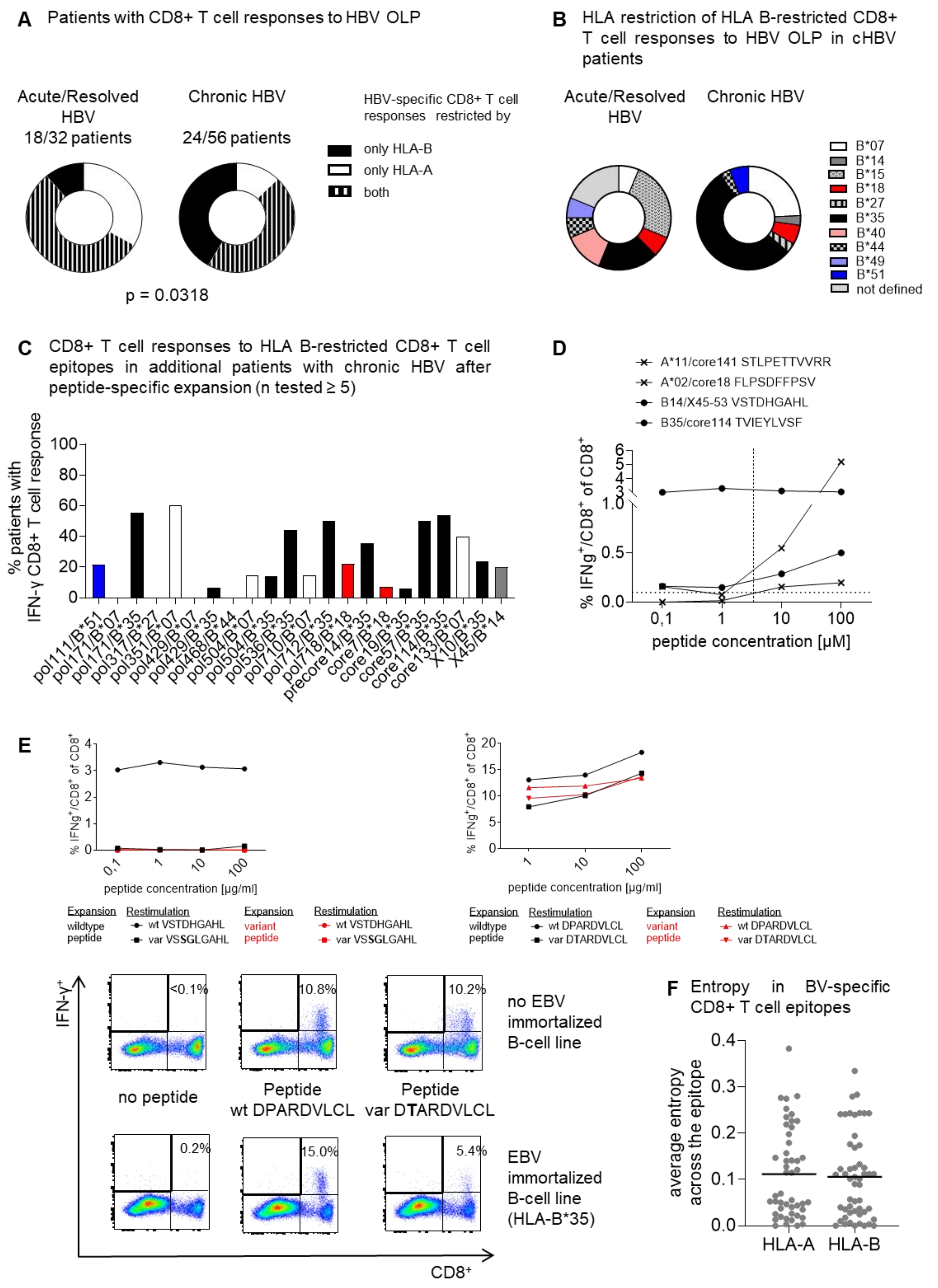

Fig. S3. Dominant HLA-B-restricted HBV-specific CD8+ T cell epitopes

18/32 patients with acute/resolved, and 24/56 patients with chronic HBV infection targeted at least one HBV OLP. Of these, the number of patients targeting only HLA-

A, HLA-B restricted epitopes or both are depicted; statistical analysis was performed with Fisher's exact test (A). HLA type of HLA-B-restricted HBV-specific CD8+ T cell responses to OLP in the patient cohort with chronic (n=56) or acute/resolved (n=32) HBV infection (B). Testing of dominant HLA-B-restricted HBV-specific CD8+ T cell epitopes in additional HLA-matched patients with chronic HBV infection after peptide-specific expansion with the minimal optimal epitope (C). Peptide titrations after peptide-specific expansion of PBMC using representative HLA-A versus HLA-B restricted epitopes. The peptide concentration used in our study (5µM) and the cutoff for HBV-specific CD8+ T cell responses (0.1% IFNg+/CD8+ of CD8+) are indicated by dotted lines (D). Testing of representative wildtype versus autologous variant epitopes by peptide titration are depicted. Loading of wildtype versus variant of epitope B*35/$_{X10-18}$ using B-lymphoblastoid cell lines (B-LCL) is shown (E). Entropy (sequence variability) in HBV-specific CD8+ T cell epitopes targeted in our study. Statistical analysis was performed with unpaired t-test (F)

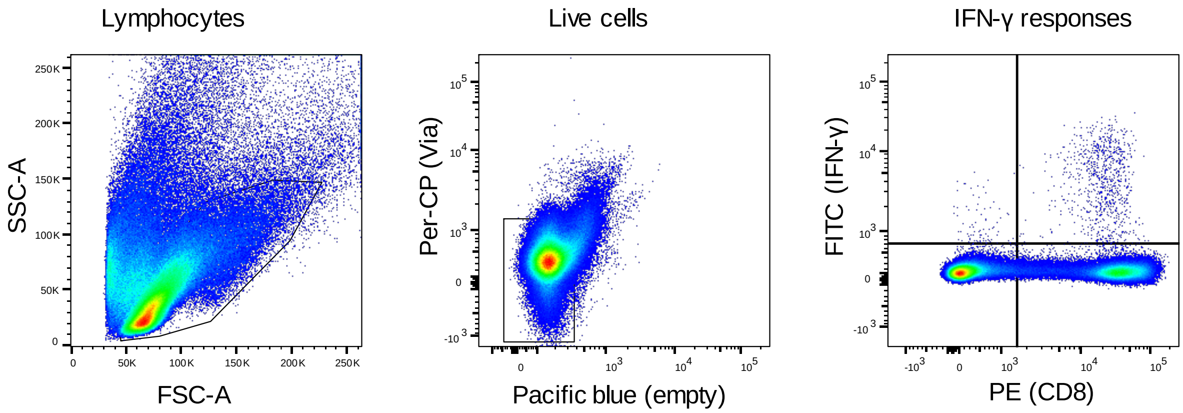

Fig. S4. Gating strategy

The gating strategy is depicted.

Table S1 Patient characteristics

Clinical parameters in the tested patient cohorts (acute, resolved and chronic HBV infection). NA = not available; NUC = nucleoside-analogues; IFN = interferon

code	HLA type	sex	age	cohort	acute/ resolved: details on clinical history	chronic: EASL clinical stage	chronic: NUC-therapy?	chronic: history of IFN treatment?	Viral load [IU/ml]	ALT [U/L]
CH-1	A*02:01, A*24:02, B*18:01, B*39:01	M	68	chronic		HBeAg negative chronic hepatitis	yes	no	<10	23
CH-2	A*02:01, A*03:01, B*35:03, B*51:01	M	49	chronic		HBeAg negative chronic hepatitis	yes	no	<10	16
CH-3	A*03:01, A*24:02, B*14:02, B*35:02	M	49	chronic		HBeAg negative chronic hepatitis	yes	yes	74	53
CH-4	A*02:01, A*24:02, B*08:01, B*1801	F	39	chronic		HBeAg negative chronic infection	no	no	962	35
CH-5	A*30:02, A*68:01, B*18:01, B*40:01	M	41	chronic		HBeAg negative chronic hepatitis	yes	no	<10	99
CH-6	A*02:01, A*24:02, B*15:01, B*1801	F	49	chronic		HBeAg negative chronic hepatitis	yes	no	<10	28
CH-7	A*01:01, A*30:01, B*35:08, B*51:01	F	26	chronic		HBeAg negative chronic infection	no	no	137	27
CH-8	A*11:01, A*24:02, B*18:01, B*37:01	M	31	chronic		HBeAg negative chronic hepatitis	yes	yes	<10	44
CH-9	A*02:01, A*03:01, B*18:01, B*4901	M	43	chronic		HBeAg negative chronic hepatitis	no	yes	10209	28
CH-10	A*02:01, A*32:01, B*08:01, B*35:03	F	42	chronic		HBeAg negative chronic infection	no	no	919	30
CH-11	A*01:01, A*24:02, B*35:03, B*44:02	M	31	chronic		HBeAg negative chronic infection	no	no	7149	23
CH-12	A*24:02, A*32:01, B*18:01, B*40:02	F	18	chronic		HBeAg negative chronic infection	no	no	2462	27
CH-13	A*24:02, A*33:01, B*14:02, B*49:01	F	58	chronic		HBeAg negative chronic infection	no	no	3871	30
CH-14	A*03:01, A*24:02, B*15:01, B*35:03	M	44	chronic		HBeAg negative chronic infection	no	no	1590	23
CH-15	A*11:01, A*24:02, B*27:02, B*44:02	M	21	chronic		HBeAg negative chronic hepatitis	yes	no	<10	49
CH-16	A*02:01, A*03:01, B*44:03, B*44:05	F	59	chronic		HBeAg negative chronic infection	no	no	2092	24

CH-17	A*02:01, A*23:01, B*07:05, B*44:03	F	42	chronic		HBeAg negative chronic infection	no	no	120	23
CH-18	A*33:01, B*14:02	F	46	chronic		HBeAg negative chronic infection	no	no	30	42
CH-19	A*03:01, A*68:02, B*27:02, B*38:01	M	33	chronic		HBeAg negative chronic hepatitis	yes	yes	<10	26
CH-20	A26:01, A*31:01, B*07:02, B*51:01	M	42	chronic		HBeAg negative chronic hepatitis	yes	no	17	71
CH-21	A*24:02, A*66:01, B*41:02, B*51:01	F	58	chronic		HBeAg negative chronic hepatitis	no	no	29625	64
CH-22	A*03:01, A*11:01, B*35:01, B*37:01	F	42	chronic		HBeAg negative chronic hepatitis	yes	no	<10	23
CH-23	A*01:01, A*02:05, B*44:02, B*51:01	M	42	chronic		HBeAg negative chronic infection	no	no	14335	34
CH-24	A*01:01, A*11:01, B*18:01, B*35:02	F	30	chronic		HBeAg negative chronic hepatitis	no	no	21727	41
CH-25	A*01:01, A*32:01, B*08:01	M	27	chronic		HBeAg negative chronic infection	no	no	4076	39
CH-26	A*01:01, A*03:01, B*37:01, B*51:01	M	30	chronic		HBeAg negative chronic hepatitis	no	no	4853	53
CH-27	A*01:01, A*03:01, B*13:02, B*35:01	M	45	chronic		HBeAg negative chronic hepatitis	yes	no	<10	33
CH-28	A*26:01, A*30:01, B*13:02, B*35:01	F	28	chronic		HBeAg negative chronic hepatitis	yes	yes	<10	48
CH-29	A*02:01, B*35:01, B*40:01	F	27	chronic		HBeAg negative chronic hepatitis	yes	no	20	30
CH-30	A*02:01, A*25:01, B*14:02, B*18:01	F	31	chronic		HBeAg negative chronic hepatitis	yes	no	<10	51
CH-31	A*03:01, A*2402, B*35:01	F	51	chronic		HBeAg negative chronic hepatitis	yes	no	87	20
CH-32	A*03:01, A*25:01, B*07:02, B*57:01	F	29	chronic		HBeAg negative chronic infection	no	no	456	12
CH-33	A*01:01, A*66:01, B*41:02, B*57:01	M	37	chronic		HBeAg negative chronic infection	no	no	1339	56
CH-34	A*03:01, A*31:01, B*35:01, B*57:01	F	41	chronic		HBeAg negative chronic infection	no	no	976	23
CH-35	A*25:01, A*32:01, B*18:01, B*57:01	M	35	chronic		HBeAg negative chronic infection	no	no	1160	42
CH-36	A*01:01, A*02:01, B*40:06, B*41:01	F	46	chronic		HBeAg negative chronic hepatitis	no	no	828919	46
CH-37	A*02:01, A*31:01, B*15:01, B*40:01	M	39	chronic		HBeAg negative chronic infection	no	no	347	30

CH-38	A*02:01, A*74:03, B*18:01, B*44:03	F	30	chronic		HBeAg negative chronic infection	no	no	288	28
CH-39	A*02:01, A*24:02, B*07:02, B*44:03	F	44	chronic		HBeAg negative chronic infection	no	no	13524	26
CH-40	A*01:01, A*02:01, B*07:02, B*18:01	M	52	chronic		HBeAg negative chronic hepatitis	yes	no	101	59
CH-41	A*24:02, A*32:01, B*35:03, B*35:08	F	49	chronic		HBeAg negative chronic infection	no	no	2220	21
CH-42	A*11:01, A*24:02, B*07:02, B*35:01	F	25	chronic		HBeAg negative chronic infection	no	no	15	34
CH-43	A*24:02, B*35:02	F	29	chronic		HBeAg negative chronic hepatitis	yes	no	11	40
CH-44	A*01:01, A*26:01, B*08:01, B*38:01	M	63	chronic		HBeAg negative chronic hepatitis	yes	no	<10	57
CH-45	A*03:01, A*03:02, B*44:02, B*51:01	M	44	chronic		HBeAg negative chronic hepatitis	yes	no	11	38
CH-46	A*03:01, A*32:01, B*13:02, B*40:02	F	68	chronic		HBeAg negative chronic hepatitis	yes	no	<10	38
CH-47	A*02:01, A*33:01, B*14:02, B*18:01	M	51	chronic		HBeAg negative chronic hepatitis	yes	no	<10	72
CH-48	A*03:02, A*11:01, B*35:01, B*41:02	M	22	chronic		HBeAg negative chronic infection	no	no	2399	27
CH-49	A*01:01, A*02:01, B*35:03, B*51:01	M	38	chronic		HBeAg negative chronic hepatitis	yes	no	<10	30
CH-50	A*30:01, B*41:01	F	37	chronic		HBeAg negative chronic hepatitis	yes	no	23764	64
CH-51	A*26:01, B*27:05, B*51:01	F	38	chronic		HBeAg negative chronic infection	no	no	117	46
CH-52	A*03:01, A*68:01, B*27:02, B*57:01	F	60	chronic		HBeAg negative chronic hepatitis	yes	no	273	19
CH-53	A*02:01, B*35:01, B*52:01	M	64	chronic		HBeAg negative chronic hepatitis	yes	no	871	28
CH-54	A*02:01, B*18:01, B*44:02	F	48	chronic		HBeAg negative chronic infection	no	no	1795	31
Ch-55	A*02:05, A*03:01, B*44:03, B*50:01	F	47	chronic		HBeAg negative chronic hepatitis	no	no	22616	50
CH-56	A*03:01, A*26:01, B*07:02, B*41:01	M	38	chronic		HBeAg negative chronic hepatitis	yes	no	3449	40
R-1	A*01:01, A*02:01, B*14:01, B*38:01	F	43	resolved	incidental serological finding of resolved HBV infection at blood donation center (anti-HBs positive, anti-HBc positive)				not applicable	68
R-2	A*02 (flow cytometry); 4-digit HLA typing NA	F	46	resolved	incidental serological finding of resolved HBV infection at check-up (anti-HBs positive, anti-HBc positive)				not applicable	29

R-3	A*01:01, A*11:01, B*40:06, B*44:03	M	51	resolved	serological finding of resolved HBV infection (anti-HBs positive, anti-HBc positive), check up because of partner with HBV				not applicable	54
R-4	A*01:01, A*02:01, B*35:01, B*51:01	F	33	resolved	incidental serological finding of resolved HBV infection at check-up (anti-HBs positive, anti-HBc positive)				not applicable	48
R-5	A*03:01, A*68:01, B*35:02, B*39:01	F	45	resolved	incidental serological finding of resolved HBV infection at check-up (anti-HBs negative, anti-HBc positive, HBsAg negative)				not applicable	34
R-6	A*03:01, A*26:01, B*15:17, B*38:01	F	44	resolved	incidental serological finding of resolved HBV infection at blood donation center (anti-HBs positive, anti-HBc positive)				not applicable	13
R-7	A*23:01, A*68:01, B*44:03, B*51:01	M	33	resolved	serological finding of resolved HBV infection (anti-HBs positive, anti-HBc positive), check up because of partner with HBV				not applicable	26
R-8	A*02:01, B*35:02, B*57:01	M	45	resolved	documented acute HBV infection with spontaneous resolution 8 years ago				not applicable	35
R-9	A*01:01, A*11:01, B*37:02, B*52:01	F	42	resolved	incidental serological finding of resolved HBV infection at check-up (anti-HBs positive, anti-HBc positive)				not applicable	22
R-10	A*03:01, A*23:01, B*07:02, B*44:03	F	44	resolved	documented acute HBV infection with spontaneous resolution 1 year ago				not applicable	43
R-11	A*02:01, A*11:01, B*15:02, B*18:01	M	31	resolved	incidental serological finding of resolved HBV infection at check-up (anti-HBs positive, anti-HBc positive)				not applicable	50
R-12	A*03:01, A*24:02, B*27:05, B*52:01	F	58	resolved	incidental serological finding of resolved HBV infection at check-up (anti-HBs positive, anti-HBc positive)				not applicable	40
R-13	A*01:01, A*24:02, B*35:02, B*38:01	M	44	resolved	incidental serological finding of resolved HBV infection at check-up (anti-HBs negative, anti-HBc positive, HBsAg negative)				not applicable	21
R-14	A*03:01, A*68:01, B*07:02, B*35:03	M	41	resolved	incidental serological finding of resolved HBV infection at check-up (anti-HBs positive, anti-HBc positive)				not applicable	27
R-15	A*01:01, A*24:02, B*07:02, B*37:01	M	56	resolved	incidental serological finding of resolved HBV infection at blood donation center (anti-HBs positive, anti-HBc positive)				not applicable	59
R-16	A*03:01, A*24:02, B*07:02, B*35:01	F	34	resolved	incidental serological finding of resolved HBV infection at check-up (anti-HBs positive, anti-HBc positive)				not applicable	7
R-17	A*02:01, B*13:02, B*44:03	F	34	resolved	incidental serological finding of resolved HBV infection at check-up (anti-HBs positive, anti-HBc positive)				not applicable	16
R-18	A*24:02, A*24:10, B*15:02, B*48:03	F	38	resolved	incidental serological finding of resolved HBV infection at check-up (anti-HBs positive, anti-HBc positive)				not applicable	17
R-19	A*11:01, A*24:02, B*13:02, B*52:01	F	35	resolved	incidental serological finding of resolved HBV infection at check-up (anti-HBs negative, anti-HBc positive, HBsAg negative)				not applicable	30
R-20	A*01:01, A*02:01, B*08:01, B*18:01	F	71	resolved	incidental serological finding of resolved HBV infection at check-up (anti-HBs positive, anti-HBc positive)				not applicable	32
R-21	A*68:01, B*15:01, B*35:03	F	63	resolved	incidental serological finding of resolved HBV infection at check-up (anti-HBs positive, anti-HBc positive)				not applicable	41
R-22	A*02:01, A*30:01, B*07:02, B*45:01	F	36	resolved	incidental serological finding of resolved HBV infection at check-up (anti-HBs negative, anti-HBc positive, HBsAg negative, HBV-PCR negative)				not applicable	13
R-23	A*02:01, B*18:01, B*51:07	M	64	resolved	incidental serological finding of resolved HBV infection at check-up (anti-HBs positive, anti-HBc positive)				not applicable	38

R-24	A*02:05, A*25:01, B*15:01, B*50:01	F	56	resolved	incidental serological finding of resolved HBV infection at check-up (anti-HBs negative, anti-HBc positive, HBsAg negative, HBV-PCR negative)				not applicable	41
R-25	A*03:01, A*25:01, B*07:02, B*40:02	F	37	resolved	incidental serological finding of resolved HBV infection at check-up (anti-HBs positive, anti-HBc positive)				not applicable	19
R-26	A*02:01, A*03:01, B*13:02, B*49:01	F	49	resolved	documented history of acute HBV infection with spontaneous resolution 5 months ago				not applicable	47
R-27	A*02:01, A*24:02, B*15:01, B*44:02	F	63	resolved	documented history of acute HBV infection with spontaneous resolution 5 years ago				not applicable	41
A-1	A*01:01, A*02:01, B*15:01, B*51:01	M	38	acute	presentation with jaundice during acute hepatitis B virus infection, later documented spontaneous resolution				291 496	3019
A-2	A*02:01, A*30:01, B*13:02, B*15:01	F	40	acute	presentation with jaundice during acute hepatitis B virus infection, later documented spontaneous resolution				6 111	7751
A-3	A*01:01, A*02:01, B*15:17, B*44:03	M	30	acute	presentation with jaundice during acute hepatitis B virus infection, later documented spontaneous resolution				751 288	2310
A-4	A*01:01, A*02:01, B*40:02, B*49:01	M	43	acute	presentation with jaundice during acute hepatitis B virus infection, later documented spontaneous resolution				50 153	748
A-5	A*02:01, A*33:01, B*07:02, B*14:02	F	45	acute	presentation with jaundice during acute hepatitis B virus infection, later documented spontaneous resolution				20 487	4372
ChR-1	A*02:01, A*24:02, B*15:13	F	60	chronic resolved	spontaneous clearance, before >5 years documented chronic HBV infection		no	no	not applicable	18
ChR-2	A*03:01, A*68:02, B*38:01, B*41:01	F	58	chronic resolved	spontaneous clearance, history of chronic HBV infection		no	no	not applicable	18
ChR-3	A*03:01, A*24:02, B*08:01, B*53:01	M	37	chronic resolved	spontaneous clearance, before >10 years documented chronic HBV infection		no	no	not applicable	28
ChR-4	A*01:01, A*68:02, B*38:01, B*52:01	F	31	chronic resolved	spontaneous clearance, before >5 years documented chronic HBV infection		no	no	not applicable	23

Table S2 Epitope fine-mapping

Details on experimental fine-mapping of minimal optimal epitopes within positive overlapping peptides.

Patient information		Responses to overlapping peptides (OLP)				Previously described optimal epitope				Best prediction optimal epitope (netMHCpan + ANN 4.0; 8/9/10-mers)					Viral sequence
**ID**	**HLA type**	**Number of positive OLP**	**Strength of response (%IFNγ⁺/ CD8⁺)**	**Position of OLP(s)**	**Sequence of OLP**	**Position**	**Sequence**	**HLA restriction**	**Experimentally validated?**	**Position**	**Sequence**	**HLA restriction**	**Epitope experimentally validated?**	**HLA-restriction experimentally validated?**	**Autologous patient's viral sequence**
CH-1	A*02:01, A*24:02, B*18:01, B*39:01	178/179	2.23	Precore 29-Core24	GMDIDPY**KEFGATVELLS**FLPSDFF	none				Core 7-15	KEFGATVEL	B*18:01	yes	yes	KEFGA**S**VEL
CH-2	A*02:01, A*03:01, B*35:03, B*51:01	62	1.22	Polymerase 428-445	LHPAAMPHLLVGSSGLSR	none				Pol 429-436	HPAAMPHL	B*35:03	yes	yes	HPAAMPHL(L)
CH-2	A*02:01, A*03:01, B*35:03, B*51:01	175	2.33	Precore 8-25	LIISCSCPTVQASKLCLG	none				Precore 14-22	CPTVQASKL	B*35:03	yes	yes	CPTVQASKL
CH-3	A*03:01, A*24:02, B*14:02, B*35:02	19	0.58	Polymerase 127-144	KGIKPYYPEHLVNHYFQT	none				Pol 132-142	YYPEHLVNHYF	A*24:02	yes	yes	(Y)YPEH**V**VNHYF
CH-3	A*03:01, A*24:02, B*14:02, B*35:02	77	43.57	Polymerase 533-550	CLAFSYMDDVVLGAKSVQ	none				Pol 536-544	FSYMDDVVL	B*35:02	yes	no	FSYMDDVVL

CH-3	A*03:01, A*24:02, B*14:02, B*35:02	102	0.30	Polymerase 708-725	FLAPLPIHTAELLAACFA	none				Pol 712-719	LPIHTAEL	B*35:02	yes	yes	LPIHTAEL
CH-3	A*03:01, A*24:02, B*14:02, B*35:02	204	0.18	X 1-18	MAARLCCQLDPARDVLCL	none				X10-18	DPARDVLCL	B*35:02	yes	yes	D**T**ARDVLCL
CH-4	A*02:01, A*24:02, B*08:01, B*1801	none													
CH-5	A*30:02, A*68:01, B*18:01, B*40:01	none													
CH-6	A*02:01, A*24:02, B*15:01, B*1801	93/94	1.18	Polymerase 645-669	PLYACIQ**SKQAFTFSPTY**KAFLCKQ	Pol 654-663	QAFTFSPTYK	A*02:01	nd						QAFTFSPTYK
CH-7	A*01:01, A*30:01, B*35:08, B*51:01	none													
CH-8	A*11:01, A*24:02, B*18:01, B*37:01	none													
CH-9	A*02:01, A*03:01,	none													

	B*18:01, B*4901														
CH-10	A*02:01, A*32:01, B*08:01, B*35:03	none													
CH-11	A*01:01, A*24:02, B*35:03, B*44:02	none													
CH-12	A*24:02, A*32:01, B*18:01, B*40:02	none													
CH-13	A*24:02, A*33:01, B*14:02, B*49:01	209	0.92	X 36-53	TLSSPSPSAVSTDHGAHL	none				X 45-53	VSTDHGAHL	B*14:02	yes	yes	VS**SGL**GAHL
CH-14	A*03:01, A*24:02, B*15:01, B*35:03	17	0.78	Polymerase 113-130	ARFYPNVTKYLPLDKGIK	none				Pol 114-123	RFYPNVTKYL	A*24:02	yes	nd	RFYPNVTKYL
CH-14	A*03:01, A*24:02, B*15:01, B*35:03	180	2.44	Core 14-31	ELLSFLPSDFFPSVRDLL	Core 19-27	LPSDFFPSV	B*35:01	yes						LP**A**DFFPS (V)
CH-15	A*11:01, A*24:02, B*27:02, B*44:02	none													

CH-16	A*02:01, A*03:01, B*44:03, B*44:05	none													
CH-17	A*02:01, A*23:01, B*07:05, B*44:03	16/17	0.32	Polymerase 106-130	RLQLIMP**ARFYPNVTKYL**PLDKGIK	none				Pol 115-123	FYPNVTKYL	A*23:01	nd	nd	FYPNVTKYL
CH-17	A*02:01, A*23:01, B*07:05, B*44:03	22	0.24	Polymerase 148-165	LHTLWKAGILYKRETTHS	Pol 149-159	HTLWKAGILYK	A*02:01	nd						HTLWKAGILYK
CH-17	A*02:01, A*23:01, B*07:05, B*44:03	24	0.31	Polymerase 162-179	TTHSASFCGSPYSWEQEL	none				Pol 171-179	SPYSWEQEL	B*07:05	yes	yes	SPYSWEQEL
CH-17	A*02:01, A*23:01, B*07:05, B*44:03	27	0.40	Polymerase 183-200	AESFHQQSSGILSRPPVG	none				Pol 192-199 / Pol 183-191	GILSRPPV / AESFHQQSS	A*02:01 / B*44:03	nd	nd	GILSRPPV / AESFHQQSS
CH-17	A*02:01, A*23:01, B*07:05, B*44:03	45	0.92	Polymerase 309-326	VFPCWWLQFRNSKPCSDY	none				Pol 309-317	VFPCWWLQF	A*23:01	yes	nd	VFPCWWLQF
CH-17	A*02:01, A*23:01, B*07:05, B*44:03	62	0.33	Polymerase 428-445	LHPAAMPHLLVGSSGLSR	none				Pol 429-437	HPAAMPHLL	B*07 / B*07:05	nd	nd	HPAAMPHLL
CH-17	A*02:01, A*23:01,	67	0.70	Polymerase 463-480	TMQNLHDSCSRNLYVSLL	none				Pol 468-476	HDSCSRNLY	B*44:03	nd	nd	HDSCSR**H**LY

	B*07:05, B*44:03														
CH-17	A*02:01, A*23:01, B*07:05, B*44:03	72	1.07	Polymerase 498-515	ILGFRKIPMGVGLSPFLL	Pol 504-512	IPMGVGLSP	B*07:02	yes						IPMGVGLSPFL
CH-17	A*02:01, A*23:01, B*07:05, B*44:03	107	1.55	Polymerase 743-760	SRKYTSFPWLLGCAANWI	Pol 752-760	LLGCAANWI	A*02:01	yes						LLGCAANWI
CH-17	A*02:01, A*23:01, B*07:05, B*44:03	224	0.34	X 141-154	LVCAPAPCNFFTSA	X 142-150	VCAPAPCNF	A*02	nd						VCAPAPCNF
CH-18	A*33:01, B*14:02	none													
CH-19	A*03:01, A*68:02, B*27:02, B*38:01	none													
CH-20	A*26:01, A*31:01, B*07:02, B*51:01	none													
CH-21	A*24:02, A*66:01, B*41:02, B*51:01	none													
CH-22	A*03:01, A*11:01,	185	2.03	Core 49-66	SPHHTALRQAILCWGELM	none				Core 57-66	QAILCWGELM	B*35:01	yes	yes	QAILCWGELM

	B*35:01, B*37:01														
CH-23	A*01:01, A*02:05, B*44:02, B*51:01	16	0.33	Polymerase 106-123	RLQLIMPARFYPNVTKYL	none				Pol 111-119	MPARFYPNV	B*51:01	yes	yes	MPARFYPNV
CH-23	A*01:01, A*02:05, B*44:02, B*51:01	79	0.62	Polymerase 547-564	KSVQHLESLFTAVTNFLL	none				Pol 556-564	FTAVTNFLL	A*02:05	yes	yes	FTAVTNFLL
CH-24	A*01:01, A*11:01, B*18:01, B*35:02	204	0.91	X 1-18	MAARLCCQLDPARDVLCL	none				X10-18	DPARDVLCL	B*35:02	yes	yes	D**T**ARDVLCL
CH-25	A*01:01, A*32:01, B*08:01	none													
CH-26	A*01:01, A*03:01, B*37:01, B*51:01	none													
CH-27	A*01:01, A*03:01, B*13:02, B*35:01	72	0.74	Polymerase 498-515	ILGFRKIPMGVGLSPFLL	none				Pol 504-513	IPMGVGLSPF	B*35:01	yes	nd	IPMGVGLSPF
CH-27	A*01:01, A*03:01, B*13:02, B*35:01	75	0.21	Polymerase 519-536	TSAICSVVRRAFPHCLAF	Pol 520-228	SAICSVVRR	A*03:01	tested negative						SAICSVVRR

CH-28	A*26:01, A*30:01, B*13:02, B*35:01	186	0.96	Core 56-73	RQAILCWGELMTLATWVG	none				Core 57-66	QAILCWGELM	B*35:01	yes	yes	nd
CH-28	A*26:01, A*30:01, B*13:02, B*35:01	193	0.81	Core 105-122	ISCLTFGRETVIEYLVSF	none				Core 113-122 / Core 114-122	ETVIEYLVSF / TVIEYLVSF	A*26:01 / B*35:01	yes / yes	yes / yes	ETV**V**EYLVSF / TV**V**EYLVSF
CH-29	A*02:01, B*35:01, B*40:01	none													
CH-30	A*02:01, A*25:01, B*14:02, B*18:01	none													
CH-31	A*03:01, A*2402, B*35:01	24	0.57	Polymerase 162-179	TTHSASFCGSPYSWEQEL	Pol 171-179	SPYSWEQEL	B*35	yes						SPYSWEQEL
CH-31	A*03:01, A*2402, B*35:01	72	0.31	Polymerase 498-515	ILGFRKIPMGVGLSPFLL	none				Pol 504-513	IPMGVGLSPF	B*35:01	yes	yes	IPMGVGLSPF
CH-32	A*03:01, A*25:01, B*07:02, B*57:01	none													
CH-33	A*01:01, A*66:01, B*41:02, B*57:01	none													

CH-34	A*03:01, A*31:01, B*35:01, B*57:01	186	0.40	Core 56-73	RQAILCWGELMTLATWVG	none				Core 57-66	QAILCWGELM	B*35:01	yes*	yes	QAILCWGELM
CH-34	A*03:01, A*31:01, B*35:01, B*57:01	194	0.22	Core 112-129	RETVIEYLVSFGVWIRTP	none				Core 114-122	TVIEYLVSF	B*35:01	yes	nd	**I**VIEYLVSF
CH-35	A*25:01, A*32:01, B*18:01, B*57:01	none													
CH-36	A*01:01, A*02:01, B*40:06, B*41:01	none													
CH-37	A*02:01, A*31:01, B*15:01, B*40:01	216	0.64	X 85-102	AHQFLPKVLHKRTLGLSV	X 92-100	(K)VLHKRTLGL	A*02:01	nd						(K)VLHKRTLGL
CH-37	A*02:01, A*31:01, B*15:01, B*40:01	219	0.36	X 106-123	TDLEAYFKDCLFKDWEEL	X 115-123	CLFKDWEEL	A*02.01	nd						CLFKDWEEL
CH-38	A*02:01, A*74:03, B*18:01, B*44:03	none													
CH-39	A*02:01, A*24:02,	none													

	B*07:02, B*44:03														
CH-40	A*01:01, A*02:01, B*07:02, B*18:01	51	0.97	Polymerase 351-368	IPRTPARVTGGVFLVDKN	Pol 351-363	IPRTPARVTGGVF	B*07:02	yes						IPRTPARVTGGVF
CH-40	A*01:01, A*02:01, B*07:02, B*18:01	72	1.07	Polymerase 498-515	ILGFRKIPMGVGLSPFLL	Pol 504-515	IPMGVGLSPFL	B*07:02	yes						IPMGVGLSPFL
CH-40	A*01:01, A*02:01, B*07:02, B*18:01	102	3.78	Polymerase 708-725	FLAPLPIHTAELLAACFA	Pol 710-719	APLPIHTAEL	B*07:02	yes						APLPIHTAEL
CH-40	A*01:01, A*02:01, B*07:02, B*18:01	206	0.28	X 15-32	VLCLRPVGAESRGRPFSG	X 15-23	VLCLRPVGA	A*02:01	nd						VLCLRPVGA
CH-41	A*24:02, A*32:01, B*35:03, B*35:08	180	0.60	Core 14-31	ELLSFLPSDFFPSVRDLL	Core 19-27	LPSDFFPSV	B*35:03	yes						LPSDFFPSV
CH-42	A*11:01, A*24:02, B*07:02, B*35:01	51	0.16	Polymerase 351-368	IPRTPARVTGGVFLVDKN	Pol 351-363	IPRTPARVTGGVF	A*11:01 / **B*07:02** / B*35:01	yes						nd
CH-42	A*11:01, A*24:02, B*07:02, B*35:01	186	1.75	Core 56-73	RQAILCWGELMTLATWVG	none				Core 57-66	QAILCWGELM	B*35:01	yes*	yes	nd

CH-42	A*11:01, A*24:02, B*07:02, B*35:01	193	1.77	Core 105-122	ISCLTFGRETVIEYLVSF	none				Core 114-122	TVIEYLVSF	B*35:01	yes	yes	TV**L**EYLVSF
CH-43	A*24:02, B*35:02	216	1.61	X 85-102	AHQFLPKVLHKRTLGLSV	none				X 89-98	LPKVLHKRTL	B*35:02	yes*	nd	LPKVLHKRTL
CH-44	A*01:01, A*26:01, B*08:01, B*38:01	none													
CH-45	A*03:01, A*03:02, B*44:02, B*51:01	none													
CH-46	A*03:01, A*32:01, B*13:02, B*40:02	none													
CH-47	A*02:01, A*33:01, B*14:02, B*18:01	70/71	0.43	Polymerase 484-508	QTFGRKL**HLYSHPIILGF**RKIPMGV	Pol 491-499 / Pol 489-497	HLYSHPIIL / KLHLYSHPI	A*02:01	yes						HLYSHPIIL / KLHLYSHPI
CH-47	A*02:01, A*33:01, B*14:02, B*18:01	92	0.34	Polymerase 638-655	CGYPALMPLYACIQSKQA	Pol 642-650	ALMPLYACI	A*02:01	tested negative						ALMPLYACI
CH-47	A*02:01, A*33:01, B*14:02, B*18:01	102/103	5.53	Polymerase 708-732	FLAPLPI**HTAELLAACFA**RSRSGAN	none				Pol 718-724	ELLAACF	B*18:01	yes	yes	ELLAACF

CH-48	A*03:02, A*11:01, B*35:01, B*41:02	none													
CH-49	A*01:01, A*02:01, B*35:03, B*51:01	16	0.68	Polymerase 106-123	RLQLIMPARFYPNVTKYL	none				Pol 111-119	MPARFYPNV	B*51:01	yes	yes	MPARFYPN**F**
CH-49	A*01:01, A*02:01, B*35:03, B*51:01	106/107	7.90	Polymerase 736-760	TDNSVVL**SRKYTSFPWLL**GCAANWI	Pol 752-760	LLGCAANWI	A*02:01	nd						LLGCAANWI
CH-50	A*30:01, B*41:01	75	0.76	Polymerase 519-536	TSAICSVVRRAFPHCLAF	none				Pol 525-533	VVRRAFPHC	A*30:01	yes	nd	VVRRAFPHC
CH-51	A*26:01, B*27:05, B*51:01	45	2.84	Polymerase 309-326	VFPCWWLQFRNSKPCSDY	none				Pol 317-326	FRNSKPCSDY	B*27:05	yes	yes	nd
CH-52	A*03:01, A*68:01, B*27:02, B*57:01	none													
CH-53	A*02:01, B*35:01, B*52:01	none													
CH-54	A*02:01, B*18:01, B*44:02	none													
CH-55	A*02:05, A*03:01,	none													

	B*44:03, B*50:01														
CH-56	A*03:01, A*26:01, B*07:02, B*41:01	70/71	0.23/0.16	Polymerase 484 - 508	QTFGRKL**HLYSHPIILGF**RKIPMGV	none				Pol 493-501	YSHPIILGF	A*26:01	nd	nd	nd
CH-56	A*03:01, A*26:01, B*07:02, B*41:01	171	0.16	Envelope 358 - 375	IWMMWYWGPSLYSILSPF	none				Env 360-369 / Env 367-375	MMWYWGPSLY / SLYSILSPF	A*03:01 / A*26:01	nd	nd	nd
CH-56	A*03:01, A*26:01, B*07:02, B*41:01	196	0.50	Core 126 - 143	IRTPPAYRPPNAPILSTL	none				Core 133-142	RPPNAPILST	B*07:02	nd	nd	nd
R-1	A*01:01, A*02:01, B*14:01, B*38:01	none													
R-2	A*02+	165/166	15.70/12.80	Envelope 316-333 / Envelope 323-340	PSSWAF**GKFLWEWASAR**FSWLSLLV	Env 324-333	FLWEWASA	A*02:01	nd						
R-3	A*01:01, A*11:01, B*40:06, B*44:03	none													
R-4	A*01:01, A*02:01, B*35:01, B*51:01	166	0.31	Envelope 323-340	KFLWEWASARFSWLSLLV	Env 324-333	FLWEWASA	A*02:01	nd						

R-4	A*01:01, A*02:01, B*35:01, B*51:01	167	0.29	Envelope 330-347	SARFSWLSLLVPFVQWFV	Env 335-343 / Env 338-347	WLSLLVPFV / LLVPFVQWFV	A*02:01 / A*02:01	nd						
R-4	A*01:01, A*02:01, B*35:01, B*51:01	172	0.30	Envelope 365-382	GPSLYSILSPFLPLLPIF	Env 370/371-379	(S)ILSPFLPLL	A*02:01	nd						
R-4	A*01:01, A*02:01, B*35:01, B*51:01	207	0.35	X 22-39	GAESRGRPFSGSLGTLSS	none				X 28-37/38	RPFSGSLGT(L)	B*35:01	yes*	nd	
R-4	A*01:01, A*02:01, B*35:01, B*51:01	212	0.57	X 57-74	GLPVCAFSSAGPCALRFT	none				X 58-66 / X65-73	LPVCAFSSA / SAGPCALRF	B*35:01	yes* / nd	nd	
R-5	A*03:01, A*68:01, B*35:02, B*39:01	none													
R-6	A*03:01, A*26:01, B*15:17, B*38:01	none													
R-7	A*23:01, A*68:01, B*44:03, B*51:01	none													

R-8	A*02:01, B*35:02, B*57:01	24	1.52	Polymerase 162-179	TTHSASFCGSPYSWEQEL	Pol 171-179	SPYSWEQEL	B*35:01	nd						
R-8	A*02:01, B*35:02, B*57:01	67	0.88	Polymerase 463-480	TMQNLHDSCSRNLYVSLL	none				Pol 471-479	CSRNLYVSL / HDSCSRNLY	B*57:01 / B*35:01	nd / nd	nd	
R-9	A*01:01, A*11:01, B*37:02, B*52:01	198	0.33	Core 140-159	LSTLPETTVVRRRGRSPR	Core 141-150	STLPETTVVR	A*11:01	yes						
R-10	A*03:01, A*23:01, B*07:02, B*44:03	none													
R-11	A*02:01, A*11:01, B*15:02, B*18:01	none													
R-12	A*03:01, A*24:02, B*27:05, B*52:01	none													
R-13	A*01:01, A*24:02, B*35:02, B*38:01	none													
R-14	A*03:01, A*68:01,	51	0.51	Polymerase 351-368	IPRTPARVTGGVFLVDKN	Pol 351-363	IPRTPARVTGGVF	B*07:02	yes						

	B*07:02, B*35:03														
R-14	A*03:01, A*68:01, B*07:02, B*35:03	105	0.37	Polymerase 729-746	SGANILGTDNSVVLSRKY	none				Pol 735-744 / Pol 737-745	GTDNSVVLSR / DNSVVLSRK	A*68:01	nd	nd	
R-15	A*01:01, A*24:02, B*07:02, B*37:01	23	0.08	Polymerase 155-172	GILYKRETTHSASFCGSP	none				Pol 160-168 / Pol 158-166	RETTHSASF / YKRETTHSA	B*37:01 / B*07:02	nd / nd	nd	
R-15	A*01:01, A*24:02, B*07:02, B*37:01	66	0.10	Polymerase 456-473	IFNHQHGTMQNLHDSCSR	none				Pol 456-464 / Pol 459-477	IFNHQHGTM / HQHGTMQNL	A*24:02 / B*37:01	yes* / nd	nd	
R-15	A*01:01, A*24:02, B*07:02, B*37:01	166	0.04	Envelope 323-340	KFLWEWASARFSWLSLLV	Env 325-333	LWEWASARF	A*24:02	yes						
R-16	A*03:01, A*24:02, B*07:02, B*35:01	none													
R-17	A*02:01, B*13:02, B*44:03	23	1.44	Polymerase 155-172	GILYKRETTHSASFCGSP	none				Pol 160-168	RETTHSASF	B*44:03	nd	nd	
R-17	A*02:01, B*13:02, B*44:03	111	0.35	Polymerase 771-788	SALNPADDPSRGRLGLSR	none				Pol 772-780	ALNPADDPS / ADDPSRGRL	A*02:01 / B*44:03	nd	nd	

R-17	A*02:01, B*13:02, B*44:03	193	2.17	Core 105-122	ISCLTFGRETVIEYLVSF	Core 107-115	CLTFGRETV	A*02:01	nd						
R-18	A*24:02, A*24:10, B*15:02, B*48:03	none													
R-19	A*11:01, A*24:02, B*13:02, B*52:01	44	0.53	Polymerase 302-319	RSQSERPVFPCWWLQFRN	none				Pol 309-317	VFPCWWLQF	A*24:02	yes	nd	
R-20	A*01:01, A*02:01, B*08:01, B*18:01	23	0.70	Polymerase 155-172	GILYKRETTHSASFCGSP	none				Pol 160-168	RETTHSASF	B*18:01	nd	nd	
R-20	A*01:01, A*02:01, B*08:01, B*18:01	172	0.41	Envelope 365-382	GPSLYSILSPFLPLLPIF	Env 370/371-379	(S)ILSPFLPLL	A*02:01	nd						
R-21	A*68:01, B*15:01, B*35:03	94	1.82	Polymerase 652-669	SKQAFTFSPTYKAFLCKQ	none				Pol 653-662	KQAFTFSPTY	B*15:01	nd	nd	
R-21	A*68:01, B*15:01, B*35:03	157	0.41	Envelope 260-277	LLDYQGMLPVCPLIPGSS	none				Env 263-272	YQGMLPVCPL	B*15:01	nd	nd	
R-21	A*68:01, B*15:01, B*35:03	198	6.05	Core 140-157	LSTLPETTVVRRRGRSPR	Core 141-151	STLPETTVVRR	A*68:01	nd						
R-22	A*02:01, A*30:01,	179	1.32	Core 7-24	KEFGATVELLSFLPSDFF	Core 18-27	FLPSDFFPSV	A*02:01	yes						

	B*07:02, B*45:01														
R-23	A*02:01, B*18:01, B*51:07	none													
R-24	A*02:01, A*03:01, B*07:02, B*15:01	none													
R-25	A*02:05, A*25:01, B*15:01, B*50:01	none													
R-26	A*02:01, A*24:02, B*15:01, B*44:02	100	0.25	Polymerase 694 - 710	GWGLVMGHQRMRGTFLAP	none				Pol 701 - 709	HQRMRGTFL	B*15:01	nd	nd	
R-26	A*02:01, A*24:02, B*15:01, B*44:02	170	0.12	Envelope 188 - 205	PTVWLSVIWMMWYWGPSL	Env 360 - 368	MMWYWGPSL	A*02:01/A*24:02	nd						
R-26	A*02:01, A*24:02, B*15:01, B*44:02	190	0.22	Core 84 - 101	LVVSYVNTNMGLKFRQLL	Core 87 - 95	SYVNTNMGL	A*24:02	nd						
R-27	A*02:01, A*03:01, B*13:02, B*49:01	63	0.19	Polymerase 435 - 452	HLLVGSSGLSRYVARLSS	Pol 455-463	GLSRYVARL	A*02:01	yes						

R-27	A*02:01, A*03:01, B*13:02, B*49:01	180	0.18	Core 14 - 31	ELLSFLPSDFFPSVR DLL	Core 18-27	FLPSDFFP SV	A*02:01	yes						
R-27	A*02:01, A*03:01, B*13:02, B*49:01	198	0.88	Core 140 - 157	LSTLPETTVVRRRG RSPR	Core 141 - 150/1	STLPETTV V(RR)	A*02:01	nd						
A-1	A*01:01, A*02:01, B*15:01, B*51:01	63	2.85	Polymerase 435 - 452	HLLVGSSGLSRYVA RLSS	Pol 455-463	GLSRYVAR L(S)	A*02:01	tested negative						
A-1	A*01:01, A*02:01, B*15:01, B*51:01	70	0.37	Polymerase 484 - 501	QTFGRKLHLYSHPIIL GF	Pol 489-497	KLHLYSHPI	A*02:01	nd						
A-1	A*01:01, A*02:01, B*15:01, B*51:01	143	0.97	Envelope 162-179	LNMENITSGFLGPLL VLQ	none				Env 162 - 171 / Env 171 - 178	LNMENITS GF / FLGPLLVL	B*15:01 / A*02:01	nd	nd	
A-2	A*02:01, A*30:01, B*13:02, B*15:01	167/168	3.97/1.08	Envelope 330-347/ Envelope 337-354	SARFSWL**SLLVPFV** **QWFV**GLSPTVW	Env 335-343	WLSLLVPF V	A*02:01	yes						
A-2	A*02:01, A*30:01, B*13:02, B*15:01	187	0.19	Core 63-80	GELMTLATWVGGNL EDPI	none				Core 63-72	GELMTLAT WV	A*02:01	yes	nd	
A-3	A*01:01, A*02:01,	143	0.61	Envelope 162-179	LNMENITSGFLGPLL VLQ	none				Env 167-175	ITSGFLGPL	B*15:17	yes	nd	

	B*15:17, B*44:03														
A-4	A*01:01, A*32:01, B*40:02, B*49:01	13/14	0.17/ 0.27	Polymerase 87-104/Polymerase 94-111	HQDIIKK**CEQFVGPLTVN**EKRRLQL	none				Pol 92-101	(C)EQFVGPLTV	B*49:01 / B*40:02	nd	nd	
A-4	A*01:01, A*32:01, B*40:02, B*49:01	43/44	4.56/ 1.71	Polymerase 295-312/ Polymerase 302-319	NLPPNSA**RSQSERPVFP**CWWLQFRN	none				Pol 305-312 / Pol 302-310	SERPVFPC / RSQSERPVF	B*40:02 / A*32:01	nd	nd	
A-4	A*01:01, A*32:01, B*40:02, B*49:01	59	0.62	Polymerase 407-422	LLSSNLSWLSLDVSAAFY	none				Pol 416-424	SLDVSAAFY	A*01:01	yes*	nd	
A-4	A*01:01, A*32:01, B*40:02, B*49:01	75	5.73	Polymerase 519-536	TSAICSVVRRAFPHCLAF	none				Pol 528-536	RAFPHCLAF	A32:01	nd	nd	
A-4	A*01:01, A*32:01, B*40:02, B*49:01	178/179	3.62/14.62	Precore 29-Core 17/ Core 7-24	GMDIDPY**KEFGATVELLS**FLPSDFF	none				Core 7-15	KEFGATVEL	B*40:02	nd	nd	
A-4	A*01:01, A*32:01, B*40:02, B*49:01	186	6.15	Core 56-73	RQAILCWGELMTLATWVG	none				Core 63-73	GELMTLATWV	B*40:02 / B*49:01	nd	nd	
A-4	A*01:01, A*32:01,	194	0.59	Core 112-129	RETVIEYLVSFGVWIRTP	Core 112-122	RETVIEYLVSF	B*49:01	nd						

	B*40:02, B*49:01														
A-5	A*02:01, A*33:01, B*07:02, B*14:02	51	1.44	Polymerase 351-368	IPRTPARVTGGVFLVDKN	Pol 357-362 / Pol 354-362/363	RVTGGVFLV / TPARVTGGV(F)	A*02:01 / B*07:02	nd						
A-5	A*02:01, A*33:01, B*07:02, B*14:02	146	1.38	Envelope 183-200	FLLTRILTIPQSLDSWWT	Env 183-191	FLLTRILTI	A*02:01	nd						
A-5	A*02:01, A*33:01, B*07:02, B*14:02	148	1.04	Envelope 197-214	SWWTSLNFLGGTTVCLGQ	Env 199-207	WTSLNFLGG	A*02:01	nd						
A-5	A*02:01, A*33:01, B*07:02, B*14:02	167	6.98	Envelope 330-347	SARFSWLSLLVPFVQWFV	Env 335-343 / Env 338-347	WLSLLVPFV / LLVPFVQWFV	A*02:01	nd						
A-5	A*02:01, A*33:01, B*07:02, B*14:02	180	4.87	Core 14-31	ELLSFLPSDFFPSVRDLL	Core 18-27 / Core 19-27	FLPSDFFPSV / LPSDFFPSV	A*02:01, B*07:02	nd						
A-5	A*02:01, A*33:01, B*07:02, B*14:02	192	0.27	Core 98-115	RQLLWFHISCLTFGRETV	Core 100-108 / Core 104-112 / Core 107-115	LLWFHISCL / HISCLTFGR / CLTFGRETV	A*02:01, A*33:01, A*02:01	nd						

A-5	A*02:01, A*33:01, B*07:02, B*14:02	196/197	11.55/ 4.69	Core 126-143/ Core 133-150	IRTPPAY**RPPNAPILSTL**PETTVVR	Core 133-140 / Core 139-148	RPPNAPIL / (IL)STLPETTV	B*07:02 / A*02:01	nd						
ChR-1	A*02:01, A*24:02, B*15:13	none													
ChR-2	A*03:01, A*68:02, B*38:01, B*41:01	219	0.72	X 106-123	TDLEAYFKDCLFKDWEEL	none				X109-118 / X108-116	EAYFKDCLFK / LEAYFKDCL	A*03:01 / B*41:01	nd	nd	
ChR-3	A*03:01, A*24:02, B*08:01, B*53:01	none													
ChR-4	A*01:01, A*68:02, B*38:01, B*52:01	79 / 80	1.9 / 0.56	Pol 560-584	KSVQHLE**SLFTAVTNFLL**SLGIHLN	none				Pol 556-564	FTAVTNFLL	A*68:02	nd	nd	

Table S3. Epitope list

List of HBV-specific CD8+ T cell epitopes targeted in this study.

**Position Genotype D**	**sequence**	**HLA Restriction**	**previously described vs new**	**minimal epitope experimentally tested/ validated**	**HLA-restriction experimentally validated**
Precore 14-22	CPTVQASKL	B*35:03	new	yes	yes
Core 7-15	KEFGATVEL	B*18:01	new	yes	yes
Core 7-15	KEFGATVEL	B*40:02	new	no	no
Core 18-27	FLPSDFFPSV	A*02:01	previously described	yes	no
Core 19-27	LPSDFFPSV	B*35:01	previously described	yes	yes
Core 19-27	LPSDFFPSV	B*35:03	previously described	yes	yes
Core 19-27	LPSDFFPSV	B*07:02	previously described	no	no
Core 57-66	QAILCWGELM	B*35:01	new	yes	yes
Core 63-72	GELMTLATWV	A*02:01	new	yes	no
Core 63-73	GELMTLATWV	B*40:02 / B*49:01	new	no	no
Core 87 - 95	SYVNTNMGL	A*24:02	previously described	no	no
Core 107-115	CLTFGRETV	A*02:01	previously described	no	no
Core 100-108	LLWFHISCL	A*02:01	previously described	no	no
Core 104-112	HISCLTFGR	A*33:01	previously described	no	no
Core 112-122	RETVIEYLVSF	B*49:01	previously described	no	no
Core 113-122	ETVIEYLVSF	A*26:01	new	yes	yes
Core 114-122	TVIEYLVSF	B*35:01	new	yes	yes
Core 133-140	RPPNAPIL	B*07:02	previously described	no	no
Core 133-142	RPPNAPILST	B*07:02	new	no	no
Core 139-148	(IL)STLPETTV	A*02:01	previously described	no	no
Core 141 - 150/1	STLPETTVV(RR)	A*02:01	previously described	no	no
Core 141-150	STLPETTVVR	A*11:01	previously described	yes	no
Core 141-151	STLPETTVVRR	A*68:01	previously described	no	no
Env 162 - 171	LNMENITSGF	B*15:01	new	no	no
Env 167-175	ITSGFLGPL	B*15:17	new	yes	no

Env 171 - 178	FLGPLLVL	A*02:01	new	no	no
Env 183-191	FLLTRILTI	A*02:01	previously described	no	no
Env 199-207	WTSLNFLGG	A*02:01	previously described	no	no
Env 263-272	YQGMLPVCPL	B*15:01	new	no	no
Env 324-333	FLWEWASA	A*02:01	previously described	no	no
Env 325-333	LWEWASARF	A*24:02	previously described	yes	no
Env 335-343	WLSLLVPFV	A*02:01	previously described	yes	no
Env 338-347	LLVPFVQWFV	A*02:01	previously described	no	no
Env 360 - 368	MMWYWGPSL	A*02:01/A*24:02	previously described	no	no
Env 360-369	MMWYWGPSLY	A*03:01	new	no	no
Env 367-375	SLYSILSPF	A*26:01	new	no	no
Env 370/371-379	(S)ILSPFLPLL	A*02:01	previously described	no	no
Pol 17-25	EAGPLEEEL	B*35	new	no	no
Pol 92-101	(C)EQFVGPLTV	B*49:01 / B*40:02	new	no	no
Pol 111-119	MPARFYPNV	B*51:01	new	yes	yes
Pol 114-123	RFYPNVTKYL	A*24:02	new	yes	no
Pol 115-123	FYPNVTKYL	A*23:01	new	no	no
Pol 132-142	YYPEHLVNHYF	A*24:02	new	yes	yes
Pol 149-159	HTLWKAGILYK	A*02:01	previously described	no	no
Pol 158-166	YKRETTHSA	B*07:02	new	no	no
Pol 160-168	RETTHSASF	B*37:01	new	no	no
Pol 160-168	RETTHSASF	B*44:03	new	no	no
Pol 160-168	RETTHSASF	B*18:01	new	no	no
Pol 171-179	SPYSWEQEL	B*35:01	previously described	yes	yes
Pol 171-179	SPYSWEQEL	B*07:05	new	yes	yes
Pol 183-191	AESFHQQSS	B*44:03	new	no	no
Pol 192-199	GILSRPPV	A*02:01	new	no	no
Pol 302-310	RSQSERPVF	A*32:01	new	no	no
Pol 305-312	SERPVFPC	B*40:02	new	no	no
Pol 309-317	VFPCWWLQF	A*23:01	new	yes	no
Pol 309-317	VFPCWWLQF	A*24:02	new	yes	no
Pol 317-326	FRNSKPCSDY	B*27:05	new	yes	yes
Pol 351-363	IPRTPARVTGGVF	B*07:02	previously described	yes	yes

Pol 354-362/363	TPARVTGGV(F)	B*07:02	previously described	no	no
Pol 357-362	RVTGGVFLV	A*02:01	previously described	no	no
Pol 416-424	SLDVSAAFY	A*01:01	new	yes*	no
Pol 429-436	HPAAMPHL	B*35:03	new	yes	yes
Pol 429-437	HPAAMPHLL	B*07	new	no	no
Pol 455-463	GLSRYVARL	A*02:01	previously described	yes	no
Pol 456-464	IFNHQHGTM	A*24:02	new	yes*	no
Pol 459-477	HQHGTMQNL	B*37:01	new	no	no
Pol 468-476	HDSCSRNLY	B*35:01	new	no	no
Pol 468-476	HDSCSRNLY	B*44:03	new	no	no
Pol 471-479	CSRNLYVSL	B*57:01	new	no	no
Pol 489-497	KLHLYSHPI	A*02:01	previously described	no	no
Pol 491-499	HLYSHPIIL	A*02:01	previously described	yes	yes
Pol 493-501	YSHPIILGF	A*26:01	new	no	no
Pol 504-512	IPMGVGLSP	B*07:02	previously described	yes	yes
Pol 504-513	IPMGVGLSPF	B*35:01	new	yes	yes
Pol 504-515	IPMGVGLSPFL	B*07:02	previously described	yes	no
Pol 525-533	VVRRAFPHC	A*30:01	new	yes	no
Pol 528-536	RAFPHCLAF	A32:01	new	no	no
Pol 536-544	FSYMDDVVL	B*35:02	new	yes	no
Pol 556-564	FTAVTNFLL	A*02:05	new	yes	yes
Pol 701 - 709	HQRMRGTFL	B*15:01	new	no	no
Pol 710-719	APLPIHTAEL	B*07:02	previously described	yes	yes
Pol 712-719	LPIHTAEL	B*35:02	new	yes	yes
Pol 718-724	ELLAACF	B*18:01	new	yes	yes
Pol 735-744	GTDNSVVLSR	A*68:01	new	no	no
Pol 737-745	DNSVVLSRK	A*68:01	new	no	no
Pol 752-760	LLGCAANWI	A*02:01	previously described	yes	yes
Pol 772-780	ALNPADDPS	A*02:01	new	no	no
Pol 776-784	ADDPSRGRL	B*44:03	new	no	no
X10-18	DPARDVLCL	B*35:02	new	yes	yes
X 15-23	VLCLRPVGA	A*02:01	previously described	no	no
X 28-37/38	RPFSGSLGT(L)	B*35:01	new	yes*	no

X 45-53	VSTDHGAHL	B*14:02	new	yes	yes
X 58-66	LPVCAFSSA	B*35:01	new	yes*	no
X 65-73	SAGPCALRF	B*35:01	new	no	no
X 89-98	LPKVLHKRTL	B*35:02	new	yes*	no
X 92-100	(K)VLHKRTLGL	A*02:01	previously described	no	no
X 115-123	CLFKDWEEL	A*02.01	previously described	no	no

*in other patient

Table S4. Primers

List of primers used to determine autologous viral sequences of patients with responses to overlapping peptides. Position indicated for consensus sequence genotype D; pubmed GenBank accession code X02496.1

name	sequence	position
HBV_Pol_1extF	CACCTCTGCCTAATCATCTCTTGT	1828-1851
HBV_Pol_1extR	CTTGAGCAGGAGTCGTGCAGGT	523-544
HBV_Pol_1F	TGACTCTAGCTACCTGGGTG	2099-2118
HBV_Pol_1R	AACGGGCAACATACCTTGAT	455-474
HBV_Pol_2extF	GTGGCTCCAGTTCAGGAACAGTA	65-87
HBV_Pol_2extR	CTACAGCCTCCTAATACAAAGACCT	1764-1788
HBV_Pol_2F	GAACATGGAGAACATCACATCAG	153-175
HBV_Pol_2R	CTCAAGGTCGGTCGTTGACATT	1681-1702

Table S5. HLA-mismatched controls

PBMC of patients with chronic HBV infection were tested after in vitro expansion with all HLA-mismatched epitopes targeted in our study. Epitopes restricted by HLA-types belonging to the same HLA supertype family as patient's HLA type were excluded for possible cross-recognition

Patient	age	Sex	HLA Type	HLA class I restriction	Protein	Sequence	IFN-γ+ of CD8+
**NC1**	**52**	**m**	**A*26:01; A*68:01; B*08:01**	A*02:01	Core 18-27	FLPSDFFPSV	negative
				A*02:01	Core 100-108	LLWFHISCL	negative
				A*02:01	Core 107-115	CLTFGRETV	negative
				A*02:01	Envelope 183-191	FLLTRILTI	negative
				A*02:01	Envelope 204-212	FLGGTTVCL	negative
				A*02:01	Envelope 324-333	FLWEWASA	negative
				A*02:01	Envelope 338-347	LLVPFVQWFV	negative
				A*02:01	Envelope 370-379	SILSPFLPLL	negative
				A*02:01	Envelope 371-379	ILSPFLPLL	negative
				A*02:01	Polymerase 156-166	ILYKRETTHSA	negative
				A*02:01	Polymerase 357-362	RVTGGVFLV	negative
				A*02:01	Polymerase 455	GLSRYVARL	negative
				A*02:01	Polymerase 491-499	HLYSHPIIL	negative
				A*02:01	Polymerase 752-760	LLGCAANWI	negative
				A*02:01	Polymerase 772-780	ALNPADDPS	negative
				A*02:01	Core 63-73	GELMTLATWV	negative
				A*02:01	Envelope 335-343	WLSLLVPFV	negative
				A*02:01	X 15-23	VLCLRPVGA	negative
				A*02:01	X 92-100	VLHKRTLGL	negative
				A*02:01	X 115-123	CLFKDWEEL	negative
				A*02:05	Polymerase 556-564	FTAVTNFLL	negative
				A*23:01	Polymerase 115-123	FYPNVTKYL	negative
				A*23:01	Polymerase 309-317	VFPCWWLQF	negative
				A*24:02	Polymerase 132-142	YYPEHLVNHYF	negative
				A*24:02	Envelope 325-333	LWEWASARF	negative
				A*24:02	Polymerase 114-123	RFYPNVTKYL	negative

**NC1**				A*24:02	Polymerase 456-464	IFNHQHGTM	negative
				B*07:02	Polymerase 351-363	IPRTPARVTGGV F	negative
				B*07:02	Polymerase 354-362	TPARVTGGV	negative
				B*07:02	Polymerase 354-363	TPARVTGGVF	negative
				B*07:02	Polymerase 710-719	APLPIHTAEL	negative
				B*07:02	Core 133-140	RPPNAPIL	negative
				B*07:02	X 146-154	APCNFFTSA	negative
				B*07:05	Polymerase 149-157	HTLWKAGIL	negative
				B*07:05	Polymerase 429-437	HPAAMPHLL	negative
				B*07:05<br>B*07:02	Polymerase 504-514	IPMGVGLSPFL	negative
				B*13:02	Polymerase 526-534	VRRAFPHCL	negative
				B*14:02	X 45-53	VSTDHGAHL	negative
				B*15:01	Envelope 263-272	YQGMLPVCPL	negative
				B*15:01	Polymerase 653-662	KQAFTFSPTY	negative
				B*15:17	Envelope 167-175	ITSGFLGPL	negative
				B*18:01	Core 7-15	KEFGATVEL	negative
				B*18:01	Polymerase 718-724	ELLAACF	negative
				B*18:01	Polymerase 640-647	YPALMPLY	negative
				B*27:05	Polymerase 317-326	FRNSKPCSDY	negative
				B*35:01	Core 57-66	QAILCWGELM	negative
				B*35:01	Core 114-122	TVIEYLVSF	negative
				B*35:01	Polymerase 171-179	SPYSWEQEL	negative
				B*35:01	Polymerase 504-513	IPMGVGLSPF	negative
				B*35:01	X 28-37	RPFSGPLGTL	negative
				B*35:01	X 58-66	LPVCAFSSA	negative
				B*35:02	Polymerase 712-719	LPIHTAEL	negative
				B*35:02	X10-18	DPARDVLCL	negative
				B*35:02	Polymerase 536-544	FSYMDDVVL	negative
				B*35:02	X 89-98	LPKVLHKRTL	negative
				B*35:03	Precore 14-22	CPTVQASKL	negative
				B*35:03	Core 19-27	LPSDFFPSV	negative
				B*35:03	Polymerase 116-123	YPNVTKYL	negative
				B*35:03	Polymerase 429-436	HPAAMPHL	negative
				B*35:03	Polymerase 745-753	KYTSFPWLL	negative

**NC1**				B*37:01<br>B*44:03	Polymerase 160-168	RETTHSASF	negative
				B*40:01	Polymerase 305-312	SERPVFPC	negative
				B*44:02	Polymerase 183-193	AESFHQQSSGI	negative
				B*44:03	Polymerase 468-476	HDSCSRNLY	negative
				B*49:01	Core 112-122	RETVIEYLVSF	negative
				B*49:01	Polymerase 92-101	CEQFVGPLTV	negative
				B*51:01	Polymerase 111-119	MPARFYPNV	negative
				B*57:01	Polymerase 471-479	CSRNLYVSL	negative
**NC2**	**31**	**m**	**A*23:01;A*33:01;**	A*01:01	Polymerase 416-424	SLDVSAAFY	negative
			**B*14:02;B*44:03**	A*02:01	Core 18-27	FLPSDFFPSV	negative
				A*02:01	Core 100-108	LLWFHISCL	negative
				A*02:01	Core 107-115	CLTFGRETV	negative
				A*02:01	Envelope 183-191	FLLTRILTI	negative
				A*02:01	Envelope 204-212	FLGGTTVCL	negative
				A*02:01	Envelope 324-333	FLWEWASA	negative
				A*02:01	Envelope 338-347	LLVPFVQWFV	negative
				A*02:01	Envelope 370-379	SILSPFLPLL	negative
				A*02:01	Envelope 371-379	ILSPFLPLL	negative
				A*02:01	Polymerase 156-166	ILYKRETTHSA	negative
				A*02:01	Polymerase 357-362	RVTGGVFLV	negative
				A*02:01	Polymerase 455	GLSRYVARL	negative
				A*02:01	Polymerase 491-499	HLYSHPIIL	negative
				A*02:01	Polymerase 752-760	LLGCAANWI	negative
				A*02:01	Polymerase 772-780	ALNPADDPS	negative
				A*02:01	Core 63-73	GELMTLATWV	negative
				A*02:01	Envelope 335-343	WLSLLVPFV	negative
				A*02:01	X 15-23	VLCLRPVGA	negative
				A*02:01	X 92-100	VLHKRTLGL	negative
				A*02:01	X 115-123	CLFKDWEEL	negative
				A*02:05	Polymerase 556-564	FTAVTNFLL	negative
				A*03:02	X 130-140	KVFVLGGCRHK	negative
				A*11:01	Core 141-150	STLPETTVVR	negative
				B*07:02	Polymerase 351-363	IPRTPARVTGGV<br>F	negative
				B*07:02	Polymerase 354-362	TPARVTGGV	negative
				B*07:02	Polymerase 354-363	TPARVTGGVF	negative

**NC2**				B*07:02	Polymerase 710-719	APLPIHTAEL	negative
				B*07:02	Core 133-140	RPPNAPIL	negative
				B*07:02	X 146-154	APCNFFTSA	negative
				B*07:05	Polymerase 149-157	HTLWKAGIL	negative
				B*07:05	Polymerase 429-437	HPAAMPHLL	negative
				B*07:05<br>B*07:02	Polymerase 504-514	IPMGVGLSPFL	negative
				B*13:02	Polymerase 526-534	VRRAFPHCL	negative
				B*15:01	Envelope 263-272	YQGMLPVCPL	negative
				B*15:01	Polymerase 653-662	KQAFTFSPTY	negative
				B*15:17	Envelope 167-175	ITSGFLGPL	negative
				B*35:01	Core 57-66	QAILCWGELM	negative
				B*35:01	Core 114-122	TVIEYLVSF	negative
				B*35:01	Polymerase 171-179	SPYSWEQEL	negative
				B*35:01	Polymerase 504-513	IPMGVGLSPF	negative
				B*35:01	X 28-37	RPFSGPLGTL	negative
				B*35:01	X 58-66	LPVCAFSSA	negative
				B*35:02	Polymerase 712-719	LPIHTAEL	negative
				B*35:02	X10-18	DPARDVLCL	negative
				B*35:02	Polymerase 536-544	FSYMDDVVL	negative
				B*35:02	X 89-98	LPKVLHKRTL	negative
				B*35:03	Precore 14-22	CPTVQASKL	negative
				B*35:03	Core 19-27	LPSDFFPSV	negative
				B*35:03	Polymerase 116-123	YPNVTKYL	negative
				B*35:03	Polymerase 429-436	HPAAMPHL	negative
				B*35:03	Polymerase 745-753	KYTSFPWLL	negative
				B*49:01	Core 112-122	RETVIEYLVSF	negative
				B*49:01	Polymerase 92-101	CEQFVGPLTV	negative
				B*51:01	Polymerase 111-119	MPARFYPNV	negative
				B*57:01	Polymerase 471-479	CSRNLYVSL	negative
**NC3**	**47**	**m**	**A*01:01; A*02:01;<br>B*35:03; B*51:01**	A*03:02	X 130-140	KVFVLGGCRHK	negative
				A*11:01	Core 141-150	STLPETTVVR	negative
				A*24:02	Polymerase 132-142	YYPEHLVNHYF	negative
				A*24:02	Envelope 325-333	LWEWASARF	negative
				A*24:02	Polymerase 114-123	RFYPNVTKYL	negative
				A*24:02	Polymerase 456-464	IFNHQHGTM	negative
				B*13:02	Polymerase 526-534	VRRAFPHCL	negative

**NC3**				B*15:01	Envelope 263-272	YQGMLPVCPL	negative
				B*15:01	Polymerase 653-662	KQAFTFSPTY	negative
				B*15:17	Envelope 167-175	ITSGFLGPL	negative
				B*18:01	Core 7-15	KEFGATVEL	0,16%
				B*18:01	Polymerase 718-724	ELLAACF	negative
				B*18:01	Polymerase 640-647	YPALMPLY	negative
				B*27:05	Polymerase 317-326	FRNSKPCSDY	negative
				B*40:01	Polymerase 305-312	SERPVFPC	negative
				B*49:01	Core 112-122	RETVIEYLVSF	negative
				B*49:01	Polymerase 92-101	CEQFVGPLTV	negative
				B*57:01	Polymerase 471-479	CSRNLYVSL	negative
**NC4**	**43**	**m**	**A*02:01; A*230:1;**	A*01:01	Polymerase 416-424	SLDVSAAFY	negative
			**B*35:01; B*51:01**	A*03:02	X 130-140	KVFVLGGCRHK	negative
				A*11:01	Core 141-150	STLPETTVVR	negative
				A*30:01	Polymerase 525-533	VVRRAFPHC	negative
				A*32:01	Polymerase 528-536	RAFPHCLAF	negative
				B*13:02	Polymerase 526-534	VRRAFPHCL	negative
				B*15:01	Envelope 263-272	YQGMLPVCPL	negative
				B*15:01	Polymerase 653-662	KQAFTFSPTY	negative
				B*15:17	Envelope 167-175	ITSGFLGPL	negative
				B*18:01	Core 7-15	KEFGATVEL	negative
				B*18:01	Polymerase 718-724	ELLAACF	negative
				B*18:01	Polymerase 640-647	YPALMPLY	negative
				B*27:05	Polymerase 317-326	FRNSKPCSDY	negative
				B*40:01	Polymerase 305-312	SERPVFPC	negative
				B*49:01	Core 112-122	RETVIEYLVSF	negative
				B*49:01	Polymerase 92-101	CEQFVGPLTV	negative
				B*57:01	Polymerase 471-479	CSRNLYVSL	negative
**NC5**	**37**	**f**	**A*02:01; A*11:01;**	A*01:01	Polymerase 416-424	SLDVSAAFY	negative
			**B*18:01; B*35:01**	A*23:01	Polymerase 115-123	FYPNVTKYL	negative
				A*23:01	Polymerase 309-317	VFPCWWLQF	negative
				A*24:02	Polymerase 132-142	YYPEHLVNHYF	Negative
				A*24:02	Envelope 325-333	LWEWASARF	Negative
				A*24:02	Polymerase 114-123	RFYPNVTKYL	Negative
				A*24:02	Polymerase 456-464	IFNHQHGTM	Negative
				A*26:01	Core 113-122	ETVIEYLVSF	Negative
				B*13:02	Polymerase 526-534	VRRAFPHCL	Negative

**NC5**				B*14:02	X 45-53	VSTDHGAHL	Negative
				B*15:01	Envelope 263-272	YQGMLPVCPL	Negative
				B*15:01	Polymerase 653-662	KQAFTFSPTY	Negative
				B*15:17	Envelope 167-175	ITSGFLGPL	Negative
				B*27:05	Polymerase 317-326	FRNSKPCSDY	Negative
				B*49:01	Core 112-122	RETVIEYLVSF	Negative
				B*49:01	Polymerase 92-101	CEQFVGPLTV	negative
				B*57:01	Polymerase 471-479	CSRNLYVSL	negative
